# Potassium Persulfate Promoted the One-Pot and Selective *Se*-Functionalization of Pyrazoles under Acidic Conditions

**DOI:** 10.1021/acsomega.4c08079

**Published:** 2024-12-17

**Authors:** Thiago J. Peglow, João Pedro S. S. C. Thomaz, Luana S. Gomes, Vanessa Nascimento

**Affiliations:** SupraSelen Laboratory, Department of Chemistry, Universidade Federal Fluminense, Institute of Chemistry, Campus do Valonguinho, 24020-141 Niterói-RJ, Brazil

## Abstract

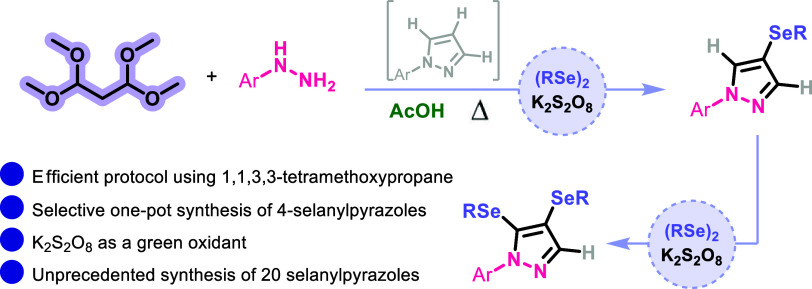

Our research presents selective direct selenylation at the C-4 pyrazole ring using K_2_S_2_O_8_ as an oxidant under simple and mild conditions. This elegant synthesis involves the one-pot method under acidic conditions, thus minimizing reaction steps and waste generation. This innovative method allowed us to create a library of 4-selanylpyrazoles in good to excellent yields. Furthermore, with slight changes in the protocol, we describe the synthesis of the unprecedented 4,5-bis-selanylpyrazole. The selectivity of the new insertion of organoselenium into the pyrazole core was demonstrated by several ^1^H and ^77^Se NMR experiments.

## Introduction

The growing search for ecologically and economically sustainable synthetic protocols for the development of complex molecules, for example, agrochemicals, pharmaceutical drugs, and intermediates, among others, is nothing new. Environmental sustainability combined with efficiency is a central issue in contemporary organic chemistry. When feasible, an effective approach is to synthesize the target compound in a single reaction vessel through sequential transformations. This synthetic strategy is known as a “one-pot” reaction and has been widely used due to its practicality, avoiding several purification processes.^1^ A successful one-pot transformation, therefore, can minimize the waste of chemicals and reaction steps, save time, and provide a significant reduction in the amount of unwanted byproducts.

**Figure 1:**
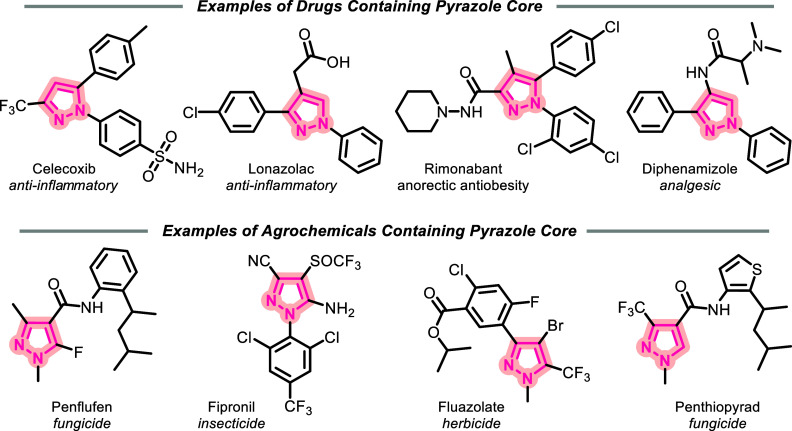
Pyrazole core: drugs and agrochemicals.

In this sense, methods for obtaining *N*-heterocycles through conventional or one-pot procedures have emerged as an eco-friendly alternative in the preparation of added-value molecules.^2^ These compounds play a crucial role across diverse domains within the field of chemistry, biology, and materials science due to their properties and wide range of applications. In particular, pyrazoles constitute a class of highly valuable compounds, characterized by their five-membered heterocyclic structure containing two adjacent nitrogen atoms.^3^ They are part of the fundamental structure of drugs^4^ including celecoxib, crizotinib, diphenamizole, lonazolac, rimonabant, mavacoxib, and razaxaban and agrochemicals such as fluazolate, penthiopyrad, bixafen, fipronil, penflufen, and pyroxasulfone (Figure 1).^5^

Organoselenium compounds represent another key class of structures worth highlighting. Studies related to the medicinal properties of this class of molecules have been growing exponentially in recent decades because of their notable biological and redox-modulating properties.^6^ This has led to extensive investigations into “bioselenium”, from *in vitro* experiments to clinical trials related to cardiovascular, autoimmune, neurodegenerative, endocrine, and psychological systems, among others.^7^

**Scheme 1:**
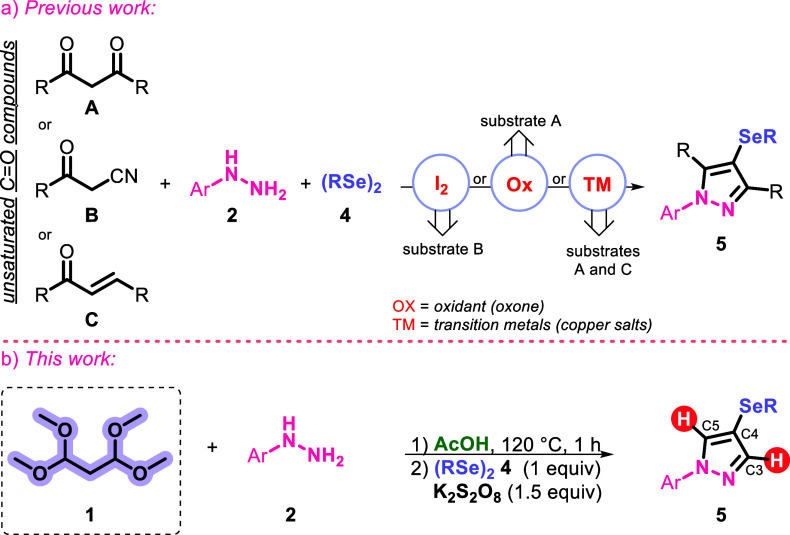
Methods to Access 4-Selanylpyrazoles: Literature vs Our Protocol

Pyrazoles can be substituted in positions 3, 4, and 5, and it is known that position 4 is the most susceptible to electrophilic addition, as described in several known halogenation reactions.^8^ To the best of our knowledge, one-pot methods for the synthesis of 4-selanylpyrazoles using diorganyl diselenides are limited. Among the most classic, the use of hydrazines with carbonyl derivatives (1,3-diketones **A**, benzoylacetonitrile **B**, or chalcones **C**) stands out. The generation of reactive selenium species is normally promoted by transition metals, oxidants, or iodine (Scheme 1a). In this way, Oliveira and co-workers^9^ developed a three-component reaction between aryl hydrazine, 1,3-diketones, and diaryl diselenides in the presence of CuBr/bpy as a catalyst for the preparation of 4-selanylpyrazoles. The same research group also described the synthesis of 4-selanylpyrazoles from chalcones using copper catalysis (CuBr/bpy) in AcOH as a solvent.^10^ Furthermore, using aryl hydrazine, 1,3-diketones, and diaryl diselenides, Fajardo and co-workers^11^ reported the multicomponent synthesis of 4-selanylpyrazoles using Cu^2+^ immobilized on alginate-based microspheres (Alg–Cu^2+^). This three-component reaction was also effectively described by Jacob and co-workers, using Oxone, and AcOH as a solvent.^12^ The same research group also reported the use of molecular iodine as a catalyst in a three-component reaction, using benzoylacetonitrile derivatives.^13^ However, these methods are limited to obtaining functionalized C3 and C5 pyrazole derivatives, not making it possible to obtain these key compounds hydrogenated in the C3, C4, and C5 positions.

An interesting point of the work reported by Jacob is the use of acetic acid (AcOH) as the solvent. Also called ethanoic acid, it is an excellent green solvent/reagent traditionally used in organic synthesis.^14^ The best-known use of AcOH is in the oxidative production of vinyl acetate monomer (VAM), which polymerizes as poly(vinyl acetate) for use in paints and adhesives. Furthermore, diluted AcOH solution of 4–6% (vinegar) is used directly as a flavoring agent for food and as a food preservative.^15^ In addition to its advantages in the chemical industry compared to the use of volatile solvents and high vapor pressure, strategies for recovering or reusing residues of this acid have been described over the years.^16^

Several organic oxidative transformations are described, including the use of organic and inorganic oxidants, such as TBHP, I_2_, Oxone, DDQ, PIDA, and DTBP. However, among all these oxidants, stable persulfate salts, especially potassium persulfate (K_2_S_2_O_8_), have demonstrated broad interest and efficiency compared to traditional oxidants. K_2_S_2_O_8_ is a stable, cheap, odorless inorganic salt with strong oxidizing properties and a low hygroscopic nature (easily manipulated).^17^ In recent decades, K_2_S_2_O_8_ has emerged as a highly effective oxidant in various oxidative transformations, generating reactive species, usually sulfate radical anion (SO_4_^•–^), under thermal or photocatalytic conditions.^17^ Among the best-known reactions, we highlight the C–H activation reactions and the formation of new carbon–carbon/carbon-heteroatom bonds,^17b,18^ and cyclization protocols.^17b,19^

Herein, we report a practical approach for the selective and one-pot synthesis of 4-selanylpyrazoles **5** using 1,1,3,3-tetramethoxypropane **1** as a new and efficient precursor. Thus, our protocol consists of obtaining the precursors 1-aryl-1*H*-pyrazoles **3***in situ* through the reaction between 1,1,3,3-tetramethoxypropane **1** (0.5 mmol) and aryl hydrazines **2** (0.5 mmol) in AcOH at 120 °C. Next, the selective selenylation reaction of the pyrazole intermediate **3** is promoted by diorganyl diselenides **4** and potassium persulfate (Scheme 1b).

## Results and Discussion

Initially, to obtain the pyrazole core, we employed the method of Finar and Hurlock (1957)^20^ with some modifications. For this study’s purpose, the reaction between 1,1,3,3-tetramethoxypropane **1** (0.5 mmol) and aryl hydrazines **2** (0.5 mmol) was conducted in the presence of AcOH (2 mL) as the catalyst/solvent of the reaction at 120 °C for 1 h in a sealed tube. This high temperature was crucial for a more efficient cyclocondensation reaction, leading to the formation of 1-phenyl-1*H*-pyrazole **3a** in quantitative yield without the necessity of a strong acid commonly used as a reaction catalyst.^21^ Considering the importance of one-pot reactions and novel *Se*-functionalized pyrazole structures, our objective was to achieve selective selenylation *in situ* through a mild and easy-to-apply method to synthesize these desired derivatives. Thus, the first reaction step was standardized, and an optimal condition was evaluated for the second reaction step to produce 1-phenyl-4-(phenylselanyl)-1*H*-pyrazole **5a** using diphenyl diselenides **4a**. Initially, we evaluated the application of classical additives for C–Se bond formation, such as iodine, copper, and iron salts (I_2_, CuI, FeCl_3_) (Table 1, entries 1–3). However, with maintaining the temperature at 120 °C, in both cases, the formation of product **5a** was not favored. We then evaluated the use of the oxidant potassium persulfate, commonly applied in the formation of electrophilic selenium species. To our satisfaction, with the addition of 1 equiv of K_2_S_2_O_8_, product **4a** was obtained in an 83% yield after 1.5 h of reaction (Table 1, entry 4). The use of 0.5 and 1.5 equiv of the oxidant of the reaction was also evaluated (Table 1, entries 5–6). In these studies, it has been observed that with excess K_2_S_2_O_8_, intermediate **3a** was completely consumed in 1.5 h and product **5a** was achieved in a 95% yield (Table 1, entry 5). Next, we tested the reaction with excess diphenyl diselenide **4a** (0.3 mmol), and product **5a** was obtained in a 97% yield (Table 1, entry 7). Due to the small increment in the yield compared to entry 5, we continue using 0.25 mmol of **4a**. Finally, the influence of temperature was evaluated in the second reaction stage (100 and 140 °C) (Table 1, entries 8–9). Thus, the reaction at 100 °C required 4 h for the consumption of intermediate **3a**, obtaining product **5a** in an 87% yield (Table 1, entry 8). In contrast, at 140 °C, the reaction was accelerated, obtaining product **5a** in an 84% yield in only 1 h of reaction. However, the formation of the bis-selenylation product **6** was observed in a 4% isolated yield (Table 1, entry 9).

**Table 1:**
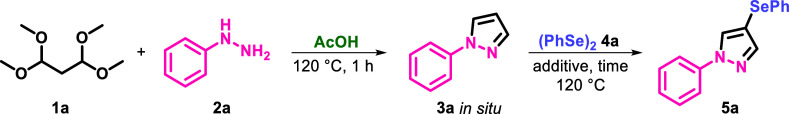
Optimization of Reaction Conditions for the Synthesis of 1-Phenyl-4-(phenylselanyl)-1*H*-pyrazole **4a**^a^

#	(PhSe)_2_ (mmol)	additive (mol %)	time (h)	yield **5a** (%)^b^
1	0.25	I_2_ (0.1)	24	NR
2	0.25	CuI (0.1)	24	NR
3	0.25	FeCl_3_ (1.0)	24	traces
4	0.25	K_2_S_2_O_8_ (1.0)	1.5	83
**5**	**0.25**	**K**_**2**_**S**_**2**_**O**_**8**_**(1.5)**	**1.5**	**95**
6	0.25	K_2_S_2_O_8_ (0.5)	24	71
7	0.30	K_2_S_2_O_8_ (1.5)	1.5	97
8^c^	0.25	K_2_S_2_O_8_ (1.5)	4.0	87
9^d^	0.25	K_2_S_2_O_8_ (1.5)	1.0	84 (4)^e^

a The reaction was carried out in the presence of 1,1,3,3-tetramethoxypropane **1a** (0.5 mmol) and phenylhydrazine **2a** (0.5 mmol) using 2.0 mL of AcOH. The reaction was kept under magnetic stirring for 1 h at 120 °C in a sealed flask, leading to in situ formation of 1-phenyl-1*H*-pyrazole **3a**. In the second reaction step, (PhSe)_2_**4a** and additives were added and, then, the reaction monitoring process was carried out by thin-layer chromatography (TLC).

b Isolated yield.

c The temperature of the second step was reduced to 100 °C.

d The temperature of the second step was increased to 140 °C.

e The formation of the product of bis-selenylation was observed. NR: no reaction.

According to the results summarized in Table 1, we assumed that the optimal one-pot reaction condition to obtain the 1-phenyl-4-(phenylselanyl)-1*H*-pyrazole **5a** consists of two simple steps. In the first step, intermediate **3a** was obtained by the reaction between 1,1,3,3-tetramethoxypropane **1** (0.5 mmol) and phenylhydrazine **2a** (0.5 mmol) in 2 mL of AcOH, after 1 h at 120 °C. In the second step, K_2_S_2_O_8_ (0.75 mmol) and (PhSe)_2_ (0.25 mmol) were added to the system, and the reaction was maintained at 120 °C for an additional time of 1.5 h, giving product **5a** in a 95% yield (Table 1, entry 5).

After establishing the optimal conditions, work was carried out to explore the scope and limitations of substrates and, in this way, analyze the impact of substituents on arylhydrazines **2** and diaryl diselenides **4a**-**i** (Table 2).

**Table 2:**
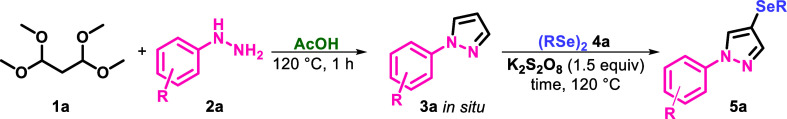

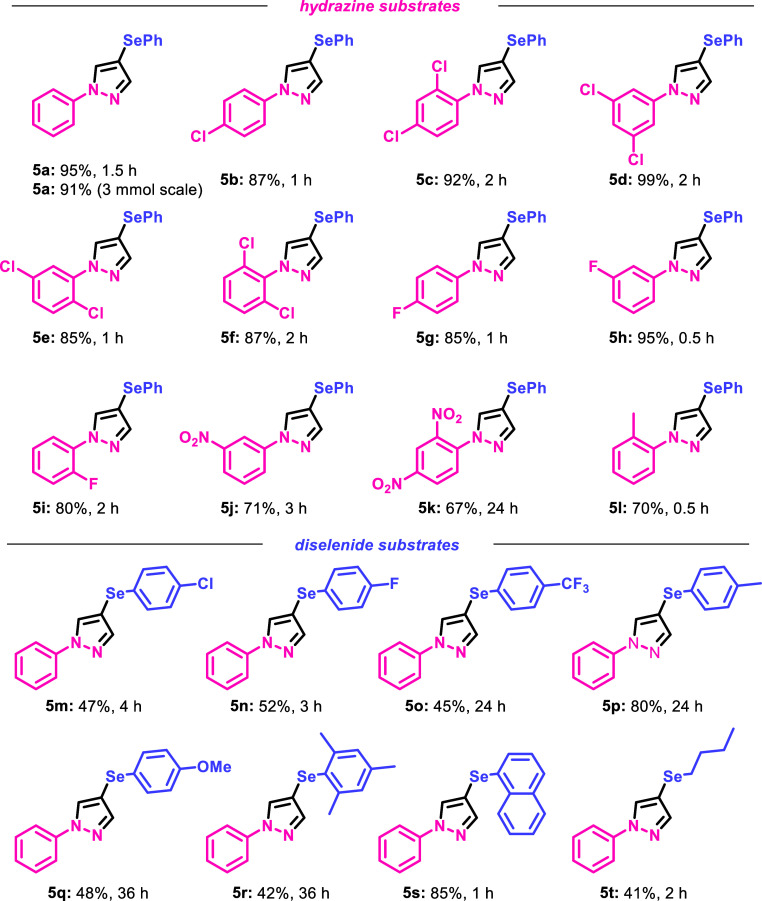
Scope Investigation of 1-Aryl-4-(organylselanyl)-1*H*-pyrazoles **5a**–**t**^a^^,^^b^

a The reaction was carried out in the presence of 1,1,3,3-tetramethoxypropane **1** (0.5 mmol) and arylhydrazines **2** (0.5 mmol) using 2.0 mL of AcOH. The reaction was kept under magnetic stirring for 1 h at 120 °C in a sealed flask, leading to in situ formation of 1-aryl-1*H*-pyrazoles **3**. In the second reaction step, (RSe)_2_**4** (0.25 mmol) and K_2_S_2_O_8_ (1.5 equiv) were added and the reaction was maintained for the indicated time.

b Isolated yields.

Thereby, 1,1,3,3-tetramethoxypropane **1** was reacted with a variety of arylhydrazines **2** mono- or disubstituted, bearing electron-donating (EDG) or -withdrawing (EWG) substituents at the aromatic ring, allowing the preparation of a series of 1-aryl-4-(organylselanyl)-1*H*-pyrazoles **5**, in moderate to excellent yields (Table 2, compounds **5b**–**l**). In general, for most cases, there was no significant change in yields when comparing the electron-donating or electron-withdrawing groups/atoms linked to the aromatic ring of the arylhydrazines **2**. However, in relation to reaction time, electron-withdrawing groups reduce the reaction speed compared to electron-donating ones. When arylhydrazines **2** containing the groups/atoms F, Cl, and NO_2_ were used, products **5a**–**j** were obtained in good to excellent yields (71–99%) with relatively short reaction times (1–3 h). In contrast, when an arylhydrazine disubstituted with the NO_2_ group (strongly electron-withdrawing) was used, the respective product **5k** was obtained after 24 h in a 67% yield. For comparison purposes, we carried out a reaction in the presence of an electron-donating group (Me) linked to arylhydrazine **2**, obtaining product **5l** in a 70% yield after only 30 min.

Finally, a library of diaryl diselenides **4b**–**f** containing EDG or EWG on the aromatic ring were employed. Noteworthily, a slight reduction in yield and an increase in reaction time between the substitutes linked to the aromatic ring of the organoselenium moiety were observed. However, when diaryl diselenides with EWG **4b** (R = 4-ClPh) and **4c** (R = 4-FPh) were used, products **5m** and **5n** were obtained in moderate yields (47 and 52%, respectively) in short reaction times (3–4 h). On the other hand, with a strong EWG **4d** (R = 4-CF_3_Ph), a longer reaction time (24 h) was required and product **5o** was obtained in a 45% yield. Moderate yields were also obtained when diaryl diselenides contained EDG **4e** (R = 4-MePh) and **4f** (R = 4-OMePh). In these cases, products **5p** and **5q** were generated in 80 and 48% yields, respectively, after 24 h of reaction. Next, the sterically hindered dimesityl diselenide **4g** and 2-naphthyl diselenide **4h** were tested which gave the corresponding products **5r** and **5s** in moderate to good yields (42 and 85%, respectively). It was observed that the sterically hindered reaction caused by **4h** was more pronounced in relation to **4g**, obtaining product **5r** (mesityl derivative) after 36 h and product **5s** (naphthyl derivative) after just 1 h. The reaction was also conducted with dibutyl diselenide **4i**, affording product **5t** in a 41% yield after 2 h. Additionally, it is important to highlight that the reaction could be easily scaled up to 3.0 mmol with the model substrates **1**, **2a**, and **4a**, affording **5a** in a 91% yield after 3 h of reaction.

**Scheme 2:**
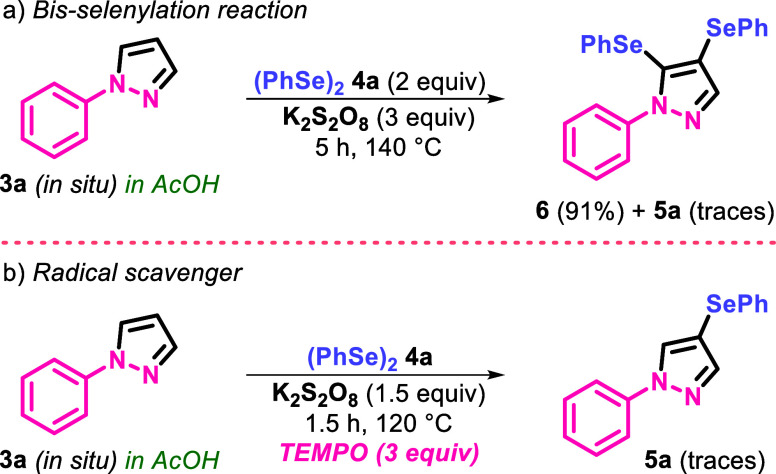
Control Experiments

**Figure 2:**
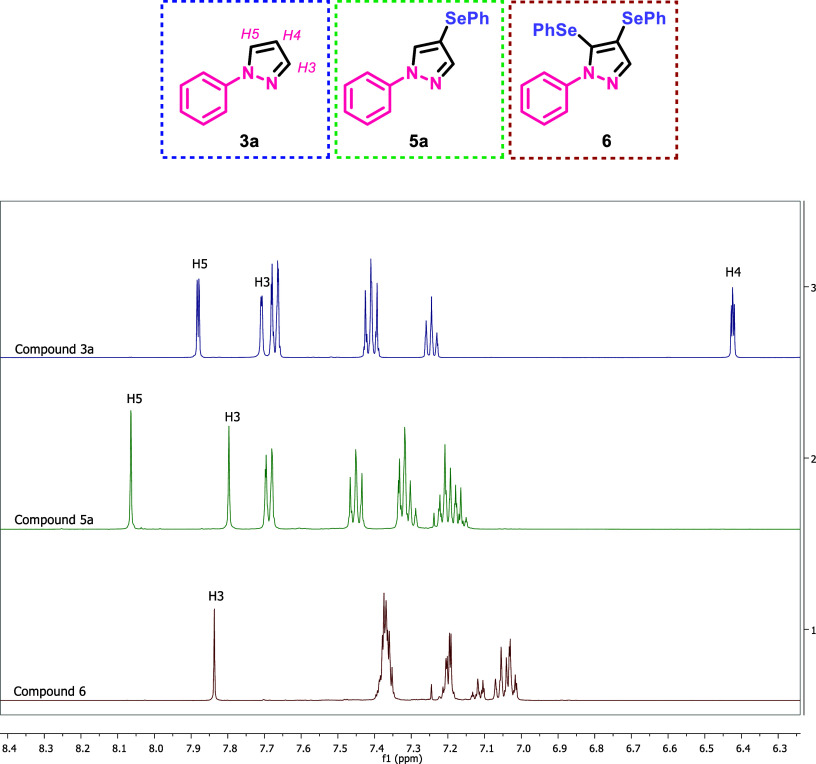
^1^H NMR experiments.

**Figure 3:**
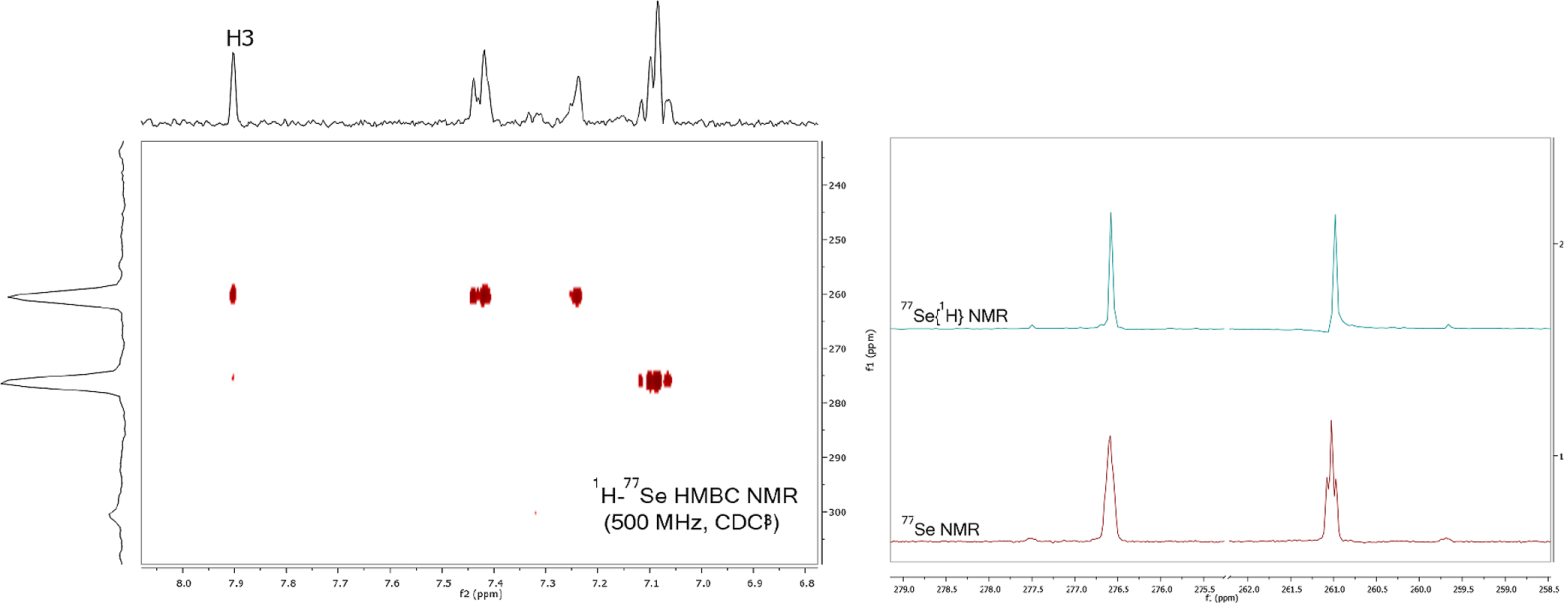
^77^Se NMR experiments of compound **6**.

In order to collect evidence to elucidate the mechanism of the synthesis of 1-aryl-4-(organylselanyl)-1*H*-pyrazoles **5**, we conducted several preliminary controlled experiments (Scheme 2). Initially, our goal was to selectively synthesize compound **6** using 2 equiv of diphenyl diselenide (PhSe)_2_**4a** (0.5 mmol) and 3 equiv of potassium persulfate (K_2_S_2_O_8_) (1.5 mmol) at 140 °C, considering the formation of the bis-selenated product **6** (Table 1, entry 9). To our satisfaction, we were able to obtain product **6** with an excellent yield of 91% after 5 h of reaction, with only traces of product **5a** observed by TLC (Scheme 2a). Notably, compound **6** is unprecedented in the literature. While the selectivity of selenylation at C-4 of the pyrazole core is established, a second insertion could potentially occur at H-3 or H-5. The selectivity for H-5 was proposed based on the chemical shifts found in the ^1^H NMR spectra of precursor **3a** reported in the literature (H-3 δ 7.72 ppm; H-4 δ 6.46 ppm; H-5 δ 7.87 ppm). Furthermore, we observed a shift of the signals referring to adjacent hydrogens due to each new insertion of an organoselenium group (Figure 2). To further support the identification of product **6**, we conducted ^77^Se NMR studies, starting with a two-dimensional ^1^H–^77^Se HMBC experiment (Figure 3). This study revealed a strong correlation (^3^*J*_Se–H3_) between the more shielded signal (δ = 261.0 ppm), corresponding to selenium bound at C-4, and a weaker correlation (^4^*J*_Se–H3_) with the more deshielded signal (δ = 276.6 ppm), which we believe corresponds to selenium bonded at C-5. In the ^77^Se{^1^H} NMR spectrum, a triplet at 261.0 ppm with a coupling constant of 4.8 Hz was detected, indicating a ^3^*J* coupling between selenium and H3 (^3^*J*_Se–H3_).^22^ We initially hypothesized that this multiplicity could also involve a ^3^*J*_Se–Se_ coupling with selenium at the C-5 position. However, this was not observed. Instead, the signal appeared as a singlet, suggesting that the expected Se–Se coupling (^3^*J*_Se–Se_) was too weak to be detected and that the coupling distance was likely too great for interaction with H3.^23^ To confirm this interpretation, we conducted additional 2D NMR analyses (^1^H–^13^C-HSQC and ^1^H–^13^C-HMBC), which supported our findings and clarified that the coupling observed was solely due to Se–H interactions (see Supporting Information—Figures S5, S6, S12, and S13.

Next, the reaction was conducted in the presence of the radical inhibitor 2,2,6,6-tetramethylpiperidin-1-oxyl (TEMPO) under standard conditions. In this case, only traces of product **5a** were observed in the reaction and the starting materials were recovered. These results suggested that the reaction might proceed through a radical pathway (Scheme 2b).

**Scheme 3:**
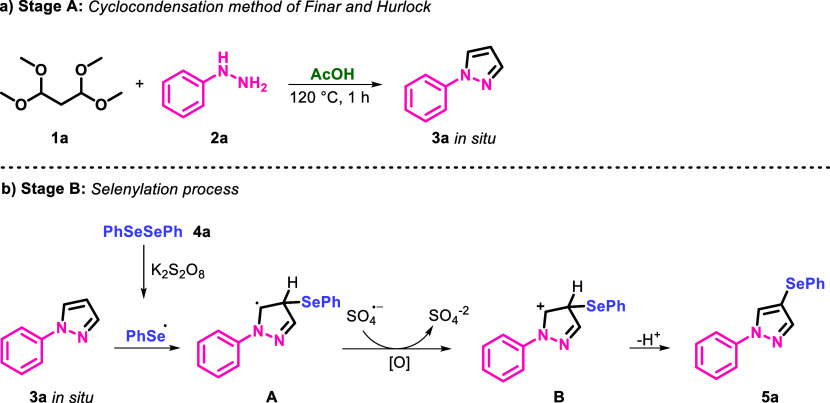
Plausible Reaction Mechanism

Based on the results of our control experiments as well as previous literature,^24^ a plausible mechanism for the formation of 1-aryl-4-(organylselanyl)-1*H*-pyrazoles **5** is described in Scheme 3. Initially, a cyclocondensation reaction occurs between 1,1,3,3-tetramethoxypropane **1** and phenyl hydrazine **2a** in the presence of AcOH at 120 °C, leading to the formation of 1-phenyl-1*H*-pyrazole **3a** (Scheme 3, Stage A). In the second stage, diphenyl diselenide **2a** was oxidized by K_2_S_2_O_8_ to generate the phenyl selenium radical. Then, the addition of a phenyl selenium radical to 1-phenyl-1*H*-pyrazole **3a** afforded pyrazolyl radical **A**. After that, it underwent further oxidation by the sulfate radical anion via a *SET* mechanism to generate the cationic intermediate **B**. Lastly, the final coupled product **5a** was formed via deprotonation (Scheme 3, Stage B). For the synthesis of compound **6**, step B is repeated.

## Conclusions

In summary, we have developed a possibility for the direct and selective selenylation in the C-4 position of unsubstituted pyrazoles using potassium persulfate in AcOH. This strategy using 1,1,3,3-tetramethoxypropane together with arylhydrazines in the preparation of 1-aryl-1*H*-pyrazole precursors is innovative and allows an effective one-pot synthesis of 4-selanylpyrazoles. This protocol allowed the synthesis of a wide range of 1-aryl-4-(organylselanyl)-1*H*-pyrazoles with variations in the hydrazine and diorganoyl diselenide moieties in good to excellent yields. Furthermore, we describe the unprecedented one-pot synthesis of a 4,5-bis-selanylpyrazole derivative with minor changes to the overall procedure and confirm the selectivity at the C-5 pyrazole position through ^77^Se NMR analyses. This approach in a single reaction bottle, without the need for prior purification of intermediates, combined with the use of a cheap and efficient oxidant and a low vapor pressure, benign, and safe solvent, further highlights the environmental sustainability of this method.

## Experimental Section

### General Information

The reagents and solvents used in the synthesis were purchased from Sigma-Aldrich and LabSynth, all of which were used without prior purification. The reactions were monitored by thin-layer chromatography (TLC) using silica gel 60 F_254_ aluminum sheets, and the visualization of the spots was done under UV light (254 nm), stained with iodine, or by the mixture between 5% of vanillin in 10% of H_2_SO_4_ and heat as developing agents. Column chromatography was performed on silica gel (230–365 mesh). ^1^H NMR spectra were obtained on Bruker AVANCE NEO 500 MHz employing a direct broadband probe at 500 MHz. The spectra were recorded in CDCl_3_ solutions. The chemical shifts are reported in parts per million, referenced to tetramethylsilane (TMS) as the internal reference. Coupling constants (*J*) are reported in Hertz. Abbreviations to denote the multiplicity of a particular signal are s (singlet), d (doublet), dd (doublet of doublets), ddd (doublet of doublet of doublets), t (triplet), td (triplet of doublets), tdd (triplet of doublet of doublets), and m (multiplet). ^13^C{^1^H} NMR spectra were obtained on Bruker AVANCE NEO 500 MHz employing a direct broadband probe at 125 MHz. The chemical shifts are reported in parts per million, referenced to the solvent peak of CDCl_3_ (δ 77.0 ppm). ^77^Se NMR spectra were obtained on Bruker AVANCE NEO 500 MHz employing a direct broadband probe at 95 MHz. The chemical shifts are reported in ppm, using as a solvent CDCl_3_ and as an internal standard diphenyl diselenide (δ 463 ppm relative to Me_2_Se δ 0 ppm). The melting points of the substances were determined in a Fisatom apparatus (Mod. 430D) series 209219. High-resolution mass spectrometry (HRMS) was recorded on a Micromass Q-TOF spectrometer, using atmospheric pressure chemical ionization (APCI).

### General Procedure for the Synthesis of 1-Aryl-4-(organylselanyl)-1*H*-pyrazoles (**5a–t**)

Compounds **5a**–**t** were prepared in two steps. In the first step, intermediate **3** was obtained by the reaction between 1,1,3,3-tetramethoxypropane **1** (0.5 mmol) and arylhydrazines **2a**–**l** (0.5 mmol) in 2 mL of AcOH, after 1 h at 120 °C. In the second step, K_2_S_2_O_8_ (1.5 equiv) and (RSe)_2_**4a**–**h** (0.25 mmol) were added to the system and the reaction was maintained at 120 °C for an additional time (Table 2). After that, the reaction was neutralized with a saturated NaHCO_3_ solution and extracted with ethyl acetate (3× 10 mL). The organic phase was separated, dried over Na_2_SO_4_, and filtered, and the solvent was evaporated under reduced pressure. The crude material was further purified by column chromatography (hexane/ethyl acetate) on silica gel.

#### 1-Phenyl-4-(phenylselanyl)-1*H*-pyrazole (**5a**)

(25) Purified by column chromatography (hexane/ethyl acetate = 97:3); yield: 0.143 g (95%); yellowish solid, mp 100–101 °C (lit. 99–101 °C); ^1^H NMR (CDCl_3_, 500 MHz): δ (ppm) 8.06 (s, 1H); 7.80 (s, 1H); 7.70–7.67 (m, 2H); 7.47–7.43 (m, 2H); 7.34–7.30 (m, 3H); 7.24–7.14 (m, 3H). ^13^C{^1^H} NMR (CDCl_3_, 125 MHz): δ (ppm) 146.5, 139.6, 132.7, 132.3, 129.5, 129.2, 126.9, 126.3, 119.1, 103.6. ^77^Se{^1^H} NMR (CDCl_3_, 95 MHz): δ (ppm) 230.8.

#### 1-(4-Chlorophenyl)-4-(phenylselanyl)-1*H*-pyrazole (**5b**)

Purified by column chromatography (hexane/ethyl acetate = 97:3); yield: 0.145 g (87%); yellowish solid, m.p: 85–87 °C; ^1^H NMR (CDCl_3_, 500 MHz): δ (ppm) 7.95 (s, 1H); 7.71 (s, 1H); 7.56 (d, *J* = 8.8 Hz, 2H); 7.35 (d, *J* = 8.8 Hz, 2H); 7.25 (d, *J* = 7.3 Hz, 2H); 7.17–7.09 (m, 3H). ^13^C{^1^H} NMR (CDCl_3_, 125 MHz): δ (ppm) 146.7, 138.1, 132.5, 132.2, 129.7, 129.6, 129.2, 126.5, 120.2, 104.2. HRMS (APCI-QTOF) calcd mass for C_15_H_12_ClN_2_Se [M + H]^+^, 334.9854; found, 334.9846. ^77^Se{^1^H} NMR (CDCl_3_, 95 MHz): δ (ppm) 232.7.

#### 1-(2,4-Dichlorophenyl)-4-(phenylselanyl)-1*H*-pyrazole (**5c**)

Purified by column chromatography (hexane/ethyl acetate = 97:3); yield: 0.169 g (92%); beige solid, mp 48–50 °C; ^1^H NMR (CDCl_3_, 500 MHz): δ (ppm) 7.942–7.941 (m, 1H); 7.72 (s, 1H); 7.47 (d, *J* = 8.6 Hz, 1H); 7.44 (d, *J* = 2.3 Hz, 1H); 7.27 (dd, *J* = 8.6 and 2.3 Hz, 1H); 7.25–7.22 (m, 2H); 7.16–7.07 (m, 3H). ^13^C{^1^H} NMR (CDCl_3_, 125 MHz): δ (ppm) 146.5, 136.6, 136.2, 134.5, 132.5, 130.4, 129.5, 129.1, 128.6, 128.2, 128.0, 126.3, 103.0. HRMS (APCI-QTOF) calcd mass for C_15_H_11_Cl_2_N_2_Se [M + H]^+^, 368.9465; found, 368.9454. ^77^Se{^1^H} NMR (CDCl_3_, 95 MHz): δ (ppm) 242.4.

#### 1-(3,5-Dichlorophenyl)-4-(phenylselanyl)-1*H*-pyrazole (**5d**)

Purified by column chromatography (hexane/ethyl acetate = 97:3); yield: 0.182 g (99%); brown oil; ^1^H NMR (CDCl_3_, 500 MHz): δ (ppm) 7.90 (s, 1H); 7.68 (s, 1H); 7.51 (d, *J* = 1.8 Hz, 2H); 7.24–7.22 (m, 2H); 7.16 (t, 1.8 Hz, 1H); 7.15–7.07 (m, 3H). ^13^C{^1^H} NMR (CDCl_3_, 125 MHz): δ (ppm) 147.0, 140.7, 135.9, 132.02, 131.95, 129.9, 129.2, 126.6, 126.5, 117.3, 105.3. HRMS (APCI-QTOF) calcd mass for C_15_H_11_Cl_2_N_2_Se [M + H]^+^, 368.9465; found, 368.9441. ^77^Se{^1^H} NMR (CDCl_3_, 95 MHz): δ (ppm) 236.7.

#### 1-(2,5-Dichlorophenyl)-4-(phenylselanyl)-1*H*-pyrazole (**5e**)

Purified by column chromatography (hexane/ethyl acetate = 97:3); yield: 0.156 g (85%); yellowish oil; ^1^H NMR (CDCl_3_, 500 MHz): δ (ppm) = 7.99 (s, 1H); 7.71 (s, 1H); 7.57 (d, *J* = 2.5 Hz, 1H); 7.33 (d, *J* = 8.6 Hz, 1H); 7.23–7.21 (m, 2H); 7.19 (dd, *J* = 8.6 and 2.5 Hz, 1H); 7.14–7.06 (m, 3H). ^13^C{^1^H} NMR (CDCl_3_, 125 MHz): δ (ppm) 146.6, 138.1, 136.5, 133.4, 132.4, 131.5, 129.5, 129.14, 129.08, 127.3, 126.4, 125.8, 103.3. HRMS (APCI-QTOF) calcd mass for C_15_H_11_Cl_2_N_2_Se [M + H]^+^, 368.9465; found, 368.9446. ^77^Se{^1^H} NMR (CDCl_3_, 95 MHz): δ (ppm) 232.7.

#### 1-(2,6-Dichlorophenyl)-4-(phenylselanyl)-1*H*-pyrazole (**5f**)

Purified by column chromatography (hexane/ethyl acetate = 97:3); yield: 0.160 g (87%); beige solid, mp 102–104 °C; ^1^H NMR (CDCl_3_, 500 MHz): δ (ppm) 7.77 (s, 1H); 7.60 (s, 1H); 7.34–7.32 (m, 2H); 7.24–7.17 (m, 3H); 7.11–7.03 (m, 3H). ^13^C{^1^H} NMR (CDCl_3_, 125 MHz): δ (ppm) 146.6, 137.3, 135.7, 134.2, 132.8, 130.9, 129.0, 128.7, 128.6, 126.0, 102.2. HRMS (APCI-QTOF) calcd mass for C_15_H_11_Cl_2_N_2_Se [M + H]^+^, 368.9465; found, 368.9450. ^77^Se{^1^H} NMR (CDCl_3_, 95 MHz): δ (ppm) 227.5.

#### 1-(4-Fluorophenyl)-4-(phenylselanyl)-1*H*-pyrazole (**5g**)

Purified by column chromatography (hexane/ethyl acetate = 97:3); yield: 0.135 g (85%); yellowish solid, mp 80–81 °C; ^1^H NMR (CDCl_3_, 500 MHz): δ (ppm) 8.00 (s, 1H); 7.78 (s, 1H); 7.67–7.63 (m, 2H); 7.34–7.31 (m, 2H); 7.25–7.12 (m, 6H). ^13^C{^1^H} NMR (CDCl_3_, 125 MHz): δ (ppm) 161.3 (d, *J* = 246.9 Hz), 146.6, 135.9 (d, *J* = 2.8 Hz), 132.6, 132.4, 129.6, 129.2, 126.4, 120.9 (d, *J* = 8.3 Hz), 116.3 (d, *J* = 23.1 Hz), 103.8. HRMS (APCI-QTOF) calcd mass for C_15_H_12_FN_2_Se [M + H]^+^, 319.0150; found, 319.0141. ^77^Se{^1^H} NMR (CDCl_3_, 95 MHz): δ (ppm) 230.5.

#### 1-(3-Fluorophenyl)-4-(phenylselanyl)-1*H*-pyrazole (**5h**)

Purified by column chromatography (hexane/ethyl acetate = 97:3); yield: 0.151 g (95%); white solid, mp 53–55 °C; ^1^H NMR (CDCl_3_, 500 MHz): δ (ppm) 8.09 (d, *J* = 2.6 Hz, 1H); 7.83 (td, *J* = 7.8 and 2.1 Hz, 1H); 7.72 (s, 1H); 7.25–7.23 (m, 2H); 7.21–7.06 (m, 6H). ^13^C{^1^H} NMR (CDCl_3_, 125 MHz): δ (ppm) 154.3 (d, *J* = 249.2 Hz), 146.2, 136.3 (d, *J* = 10.8 Hz), 132.6, 129.5, 129.1, 128.1 (d, *J* = 7.8 Hz), 127.8 (d, *J* = 9.2 Hz), 126.3, 125.0 (d, *J* = 3.6 Hz), 124.0, 116.8 (d, *J* = 20.3 Hz), 103.5. HRMS (APCI-QTOF) calcd mass for C_15_H_12_FN_2_Se [M + H]^+^, 319.0150; found, 319.0150. ^77^Se{^1^H} NMR (CDCl_3_, 95 MHz): δ (ppm) 231.6.

#### 1-(2-Fluorophenyl)-4-(phenylselanyl)-1*H*-pyrazole (**5i**)

Purified by column chromatography (hexane/ethyl acetate = 97:3); yield: 0.127 g (80%); white solid, mp 84–85 °C; ^1^H NMR (CDCl_3_, 500 MHz): δ (ppm) 7.96 (s, 1H); 7.70 (s, 1H); 7.41–7.29 (m, 3H); 7.25–7.23 (m, 2H); 7.16–7.08 (m, 3H); 6.91 (tdd, *J* = 8.1, 2.4, and 1.0 Hz, 1H). ^13^C{^1^H} NMR (CDCl_3_, 125 MHz): δ (ppm) 163.2 (d, *J* = 247.2 Hz), 146.7, 140.8 (d, *J* = 10.3 Hz), 132.4, 132.2, 130.8 (d, *J* = 9.0 Hz), 129.7, 129.2, 126.5, 114.1 (d, *J* = 2.9 Hz), 113.6 (d, *J* = 21.2 Hz), 106.8 (d, *J* = 26.5 Hz), 104.3. HRMS (APCI-QTOF) calcd mass for C_15_H_12_FN_2_Se [M + H]^+^, 319.0150; found, 319.0141. ^77^Se{^1^H} NMR (CDCl_3_, 95 MHz): δ (ppm) 233.3.

#### 1-(3-Nitrophenyl)-4-(phenylselanyl)-1*H*-pyrazole (**5j**)

Purified by column chromatography (hexane/ethyl acetate = 90:10); yield: 0.122 g (71%); yellowish solid, m.p: 80–81 °C; ^1^H NMR (CDCl_3_, 500 MHz): δ (ppm) 8.46 (t, *J* = 2.2 Hz, 1H); 8.07 (s, 1H); 8.05 (ddd, *J* = 8.2, 2.1, and 0.8 Hz, 1H); 7.98 (ddd, *J* = 8.2, 2.1, and 0.8 Hz, 1H); 7.73 (s, 1H); 7.55 (t, *J* = 8.2 Hz, 1H); 7.27–7.25 (m, 2H); 7.17–7.09 (m, 3H). ^13^C{^1^H} NMR (CDCl_3_, 125 MHz): δ (ppm) 148.9, 147.2, 140.2, 132.0, 131.9, 130.5, 130.0, 129.2, 126.7, 124.2, 121.1, 113.7, 105.5. HRMS (APCI-QTOF) calcd mass for C_15_H_12_N_3_O_2_Se [M + H]^+^, 346.0095; found, 346.0086. ^77^Se{^1^H} NMR (CDCl_3_, 95 MHz): δ (ppm) 237.1.

#### 1-(2,4-Dinitrophenyl)-4-(phenylselanyl)-1*H*-pyrazole (**5k**)

Purified by column chromatography (hexane/ethyl acetate = 90:10); yield: 0.131 g (67%); orange solid, mp 123–125 °C; ^1^H NMR (CDCl_3_, 500 MHz): δ (ppm) 8.63 (d, *J* = 2.4 Hz, 1H); 8.45 (dd, *J* = 8.8 and 2.4 Hz, 1H); 7.83 (s, 1H); 7.77–7.75 (m, 2H); 7.30–7.27 (m, 2H); 7.20–7.14 (m, 3H). ^13^C{^1^H} NMR (CDCl_3_, 125 MHz): δ (ppm) 148.5, 145.8, 143.2, 136.7, 134.2, 131.2, 130.4, 129.4, 127.6, 127.0, 125.9, 121.2, 107.4. HRMS (APCI-QTOF) calcd mass for C_15_H_11_N_4_O_4_Se [M + H]^+^, 390.9946; found, 390.9924. ^77^Se{^1^H} NMR (CDCl_3_, 95 MHz): δ (ppm) 231.7.

#### 4-(Phenylselanyl)-1-(*o*-tolyl)-1*H*-pyrazole (**5l**)

Purified by column chromatography (hexane/ethyl acetate = 97:3); yield: 0.110 g (70%); colorless oil; ^1^H NMR (CDCl_3_, 500 MHz): δ (ppm) 7.73 (s, 1H); 7.68 (s, 1H); 7.28–7.19 (m, 6H); 7.17–7.08 (m, 3H); 2.19 (s, 3H). ^13^C{^1^H} NMR (CDCl_3_, 125 MHz): δ (ppm) 145.9, 139.4, 136.2, 133.5, 133.0, 131.4, 129.2, 129.1, 128.7, 126.6, 126.2, 126.0, 101.9, 18.0. HRMS (APCI-QTOF) calcd mass for C_16_H_15_N_2_Se [M + H]^+^, 315.0400; found, 315.0388. ^77^Se{^1^H} NMR (CDCl_3_, 95 MHz): δ (ppm) 226.8.

#### 4-((4-Chlorophenyl)selanyl)-1-phenyl-1*H*-pyrazole (**5m**)

Purified by column chromatography (hexane/ethyl acetate = 97:3); yield: 0.078 g (47%); white solid, mp 107–109 °C; ^1^H NMR (CDCl_3_, 500 MHz): δ (ppm) 8.00 (s, 1H); 7.72 (s, 1H); 7.62 (d, *J* = 7.8 Hz, 2H); 7.40 (t, *J* = 7.8 Hz, 2H); 7.26 (t, *J* = 7.8 Hz, 1H); 7.17 (d, *J* = 8.7 Hz, 2H); 7.11 (d, *J* = 8.7 Hz, 2H). ^13^C{^1^H} NMR (CDCl_3_, 125 MHz): δ (ppm) 146.4, 139.5, 132.5, 132.4, 131.0, 130.8, 129.6, 129.3, 127.1, 119.2, 103.3. HRMS (APCI-QTOF) calcd mass for C_15_H_12_ClN_2_Se [M + H]^+^, 334.9854; found, 334.9846. ^77^Se{^1^H} NMR (CDCl_3_, 95 MHz): δ (ppm) 231.7.

#### 4-((4-Fluorophenyl)selanyl)-1-phenyl-1*H*-pyrazole (**5n**)

Purified by column chromatography (hexane/ethyl acetate = 97:3); yield: 0.083 g (52%), orange solid, mp 92–94 °C; ^1^H NMR (CDCl_3_, 500 MHz): δ (ppm) 7.96 (s, 1H); 7.69 (s, 1H); 7.59 (d, *J* = 7.8 Hz, 2H); 7.36 (t, *J* = 7.8 Hz, 2H), 7.25–7.20 (m, 3H); 6.84 (t, *J* = 8.7 Hz, 2H). ^13^C{^1^H} NMR (CDCl_3_, 125 MHz): δ (ppm) 161.9 (d, *J* = 246.0 Hz), 146.2, 139.5, 132.0, 131.9 (d, *J* = 7.5 Hz), 129.5, 127.0, 126.9 (d, *J* = 3.5 Hz), 119.1, 116.3 (d, *J* = 21.5 Hz), 104.2. HRMS (APCI-QTOF) calcd mass for C1_5_H_12_FN_2_Se [M + H]^+^, 319.0150; found, 319.0146. ^77^Se{^1^H} NMR (CDCl_3_, 95 MHz): δ (ppm) 229.8.

#### 1-Phenyl-4-((4-(trifluoromethyl)phenyl)selanyl)-1*H*-pyrazole (**5o**)

Purified by column chromatography (hexane/ethyl acetate = 97:3); yield: 0.083 g (45%), yellow solid, mp 107–109 °C; ^1^H NMR (CDCl_3_, 500 MHz): δ (ppm) 8.04 (s, 1H); 7.75 (s, 1H); 7.64 (d, *J* = 8.7 Hz, 2H); 7.42–7.34 (m, 4H); 7.30–7.22 (m, 3H). ^13^C{^1^H} NMR (CDCl_3_, 125 MHz): δ (ppm) 146.7, 139.5, 138.5, 132.8, 129.6, 128.7, 128.3 (q, *J* = 32.6 Hz), 127.2, 125.8 (d, *J* = 3.4 Hz), 124.1 (q, *J* = 270.2 Hz), 123.1 (d, *J* = 3.4 Hz), 119.2, 102.1. HRMS (APCI-QTOF) calcd mass for C_16_H_12_F_3_N_2_Se [M + H]^+^, 369.0118; found, 369.0108. ^77^Se{^1^H} NMR (CDCl_3_, 95 MHz): δ (ppm) 240.6.

#### 1-Phenyl-4-(*p*-tolylselanyl)-1*H*-pyrazole (**5p**)

Purified by column chromatography (hexane/ethyl acetate = 97:3); yield: 0.126 g (80%); orange solid, mp 98–99 °C; ^1^H NMR (CDCl_3_, 500 MHz): δ (ppm) 7.97 (s, 1H); 7.71 (s, 1H); 7.61 (d, *J* = 7.7 Hz, 2H); 7.38 (t, *J* = 7.7 Hz, 2H); 7.23 (t, *J* = 7.7, 1H); 7.19 (d, *J* = 8.0 Hz, 2H); 6.97 (d, *J* = 8.0 Hz, 2H); 2.21 (s, 3H). ^13^C{^1^H} NMR (CDCl_3_, 125 MHz): δ (ppm) 146.3, 139.6, 136.5, 132.0, 130.2, 130.0, 129.5, 128.7, 126.9, 119.1, 104.3, 21.0. HRMS (APCI-QTOF) calcd mass for C_16_H_15_N_2_Se [M + H]^+^, 315.0400; found, 315.0392. ^77^Se{^1^H} NMR (CDCl_3_, 95 MHz): δ (ppm) 225.9.

#### 4-((4-Methoxyphenyl)selanyl)-1-phenyl-1*H*-pyrazole (**5q**)

Purified by column chromatography (hexane/ethyl acetate = 90:10); yield: 0.079 g (48%); yellowish solid, mp 110–111 °C; ^1^H NMR (CDCl_3_, 500 MHz): δ (ppm) 7.94 (s, 1H); 7.68 (s, 1H); 7.60–7.58 (m, 2H); 7.38–7.35 (m, 2H); 7.28 (d, *J* = 8.8 Hz, 2H); 7.23–7.20 (m, 1H); 6.71 (d, *J* = 8.8 Hz, 2H); 3.68 (s, 3H). ^13^C{^1^H} NMR (CDCl_3_, 125 MHz): δ (ppm) 159.0, 145.9, 139.6, 132.7, 131.4, 129.5, 126.8, 122.1, 119.1, 114.9, 105.4, 55.3. HRMS (APCI-QTOF) calcd mass for C_16_H_15_N_2_OSe [M + H]^+^, 331.0350; found, 331.0344. ^77^Se{^1^H} NMR (CDCl_3_, 95 MHz): δ (ppm) 224.4.

#### 4-(Mesitylselanyl)-1-phenyl-1*H*-pyrazole (**5r**)

Purified by column chromatography (hexane/ethyl acetate = 97:3); yield: 0.072 g (42%); white solid, mp 122–124 °C; ^1^H NMR (CDCl_3_, 500 MHz): δ (ppm) 7.67 (s, 1H); 7.51 (d, *J* = 7.9 Hz, 2H); 7.47 (s, 1H); 7.32 (t, *J* = 7.9 Hz, 1H); 7.18–7.15 (m, 1H); 7.19 (d, *J* = 8.0 Hz, 2H); 6.85 (s, 2H); 2.48 (s, 6H); 2.17 (s, 3H). ^13^C{^1^H} NMR (CDCl_3_, 125 MHz): δ (ppm) 144.1, 142.6, 139.7, 138.8, 129.4, 129.0, 128.8, 128.1, 126.5, 118.9, 106.8, 24.4, 20.9. HRMS (APCI-QTOF) calcd mass for C_18_H_19_N_2_Se [M + H]^+^, 343.0713; found, 343.0708. ^77^Se{^1^H} NMR (CDCl_3_, 95 MHz): δ (ppm) 227.5.

#### 4-(Naphthalen-1-ylselanyl)-1-phenyl-1*H*-pyrazole (**5s**)

Purified by column chromatography (hexane/ethyl acetate = 97:3); yield: 0.149 g (85%), yellow solid, mp 87–88 °C; ^1^H NMR (CDCl_3_, 500 MHz): δ (ppm) 8.21–8.19 (m, 1H); 7.975–7.974 (m, 1H); 7.75–7.73 (m, 1H); 7.73 (s, 1H); 7.63 (d, *J* = 8.2 Hz, 1H); 7.60–7.58 (m, 2H); 7.50–7.47 (m, 1H); 7.44–7.41 (m, 1H); 7.37–7.33 (m, 3H); 7.22–7.18 (m, 2H). ^13^C{^1^H} NMR (CDCl_3_, 125 MHz): δ (ppm) 146.4, 139.6, 133.9, 132.5, 132.2, 131.5, 129.5, 128.7, 128.6, 127.4, 126.9, 126.5, 126.3, 125.99, 125.97, 119.1, 103.5. HRMS (APCI-QTOF) calculated mass for C_19_H_15_N_2_Se [M + H]^+^, 351.0400; found, 351.0397. ^77^Se{^1^H} NMR (CDCl_3_, 95 MHz): δ (ppm) 188.3.

#### 4-(Butylselanyl)-1-phenyl-1*H*-pyrazole (**5t**)

Purified by column chromatography (hexane/ethyl acetate = 97:3); yield: 0.057 g (41%); yellowish solid, mp 42–44C; ^1^H NMR (CDCl_3_, 500 MHz): δ (ppm) 7.88 (s, 1H); 7.64 (s, 1H); 7.61–7.59 (m, 2H); 7.39–7.36 (m, 2H); 7.23–7.20 (m, 1H); 2.63 (t, *J* = 7.5 Hz, 2H); 1.57 (quint, *J* = 7.4 Hz, 2H); 1.33 (sext, *J* = 7.4 Hz, 2H); 0.82 (t, *J* = 7.4 Hz, 3H). ^13^C{^1^H} NMR (CDCl_3_, 125 MHz): δ (ppm) 146.1, 139.7, 131.4, 129.4, 126.7, 119.0, 104.0, 32.4, 29,4, 22.6, 13.5. HRMS (APCI-QTOF) calcd mass for C_13_H_17_N_2_Se [M + H]^+^, 281.0557; found, 281.0541. ^77^Se{^1^H} NMR (CDCl_3_, 95 MHz): δ (ppm) 227.5.

### General Procedure for the Synthesis of 1-Phenyl-4,5-bis(phenylselanyl)-1*H*-pyrazole (**6**)

Compound **6** was prepared in two steps. In the first step, intermediate **3a** was obtained by the reaction between 1,1,3,3-tetramethoxypropane **1** (0.5 mmol) and phenylhydrazine **2a** (0.5 mmol) in 2 mL of AcOH, after 1 h at 120 °C. In the second step, K_2_S_2_O_8_ (3 equiv) and (PhSe)_2_**4a** (0.5 mmol) were added to the system and the reaction was maintained at 140 °C for 5 additional hours. After that, the reaction was neutralized with a saturated NaHCO_3_ solution and extracted with ethyl acetate (3× 10 mL). The organic phase was separated, dried over Na_2_SO_4_, and filtered, and the solvent was evaporated under reduced pressure. The crude material was further purified by column chromatography (hexane/ethyl acetate) on silica gel.

#### 1-Phenyl-4,5-bis(phenylselanyl)-1*H*-pyrazole (**6**)

Purified by column chromatography (hexane/ethyl acetate = 97:3); yield: 0.207 g (91%); yellowish solid, mp 82–84 °C; ^1^H NMR (CDCl_3_, 500 MHz): δ (ppm) 7.84 (s, 1H); 7.40–7.35 (m, 7H); 7.22–7.18 (m, 3H); 7.14–7.09 (m, 1H); 7.07–7.01 (m, 4H). ^13^C{^1^H} NMR (CDCl_3_, 125 MHz): δ (ppm) 145.8, 140.2, 133.1, 131.8, 131.2, 130.9, 130.4, 129.20, 129.15, 128.6, 128.4, 127.2, 126.7, 126.0, 114.7. HRMS (APCI-QTOF) calcd mass for C_21_H_17_N_2_Se_2_ [M + H]^+^, 456.9722; found, 456.9722. ^77^Se{^1^H} NMR (CDCl_3_, 95 MHz): δ (ppm) 276.6, 261.0. ^77^Se NMR (CDCl_3_, 95 MHz): δ (ppm) 276.6, 261.0 (t, *J* = 4.8 Hz).

## Data Availability

The data underlying this study are available in the published article and its online Supporting Information.

## Supporting Information Available

Copies of ^1^H and ^13^C{^1^H} spectra of all the synthesized products and detailed experimental procedures (PDF)
