# Supplementary material for: Potassium Persulfate Promoted the One-Pot and Selective Se-Functionalization of Pyrazoles under Acidic Conditions
Source: ACS Omega. 2024 Dec 17;9(52):51295–305. doi: 10.1021/acsomega.4c08079 (PMC11696416; doi:10.1021/acsomega.4c08079)
Supplement: Supplementary file 1 — ao4c08079_si_001.pdf [file ao4c08079_si_001.pdf]

SUPPORTING INFORMATION

Potassium Persulfate Promoted the One-Pot and Selective *Se*-Functionalization of Pyrazoles under Acidic Conditions

Thiago J. Peglow,*[a] João Pedro S. S. C. Thomaz,[a] Luana S. Gomes,[a] Vanessa Nascimento*[a]

[a] SupraSelen Laboratory, Department of Chemistry, Universidade Federal Fluminense, Institute of Chemistry, Campus do Valonguinho, 24020-141, Niterói-RJ, Brazil.

Corresponding Authors: nascimentovanessa@id.uff.br (V.N.); thiago09_tjp@hotmail.com (T.J.P.)

	Page
**Copies of (^{1}H, ^{13}C, ^{77}Se, HSQC and HMBC) NMR spectra**	**S2**

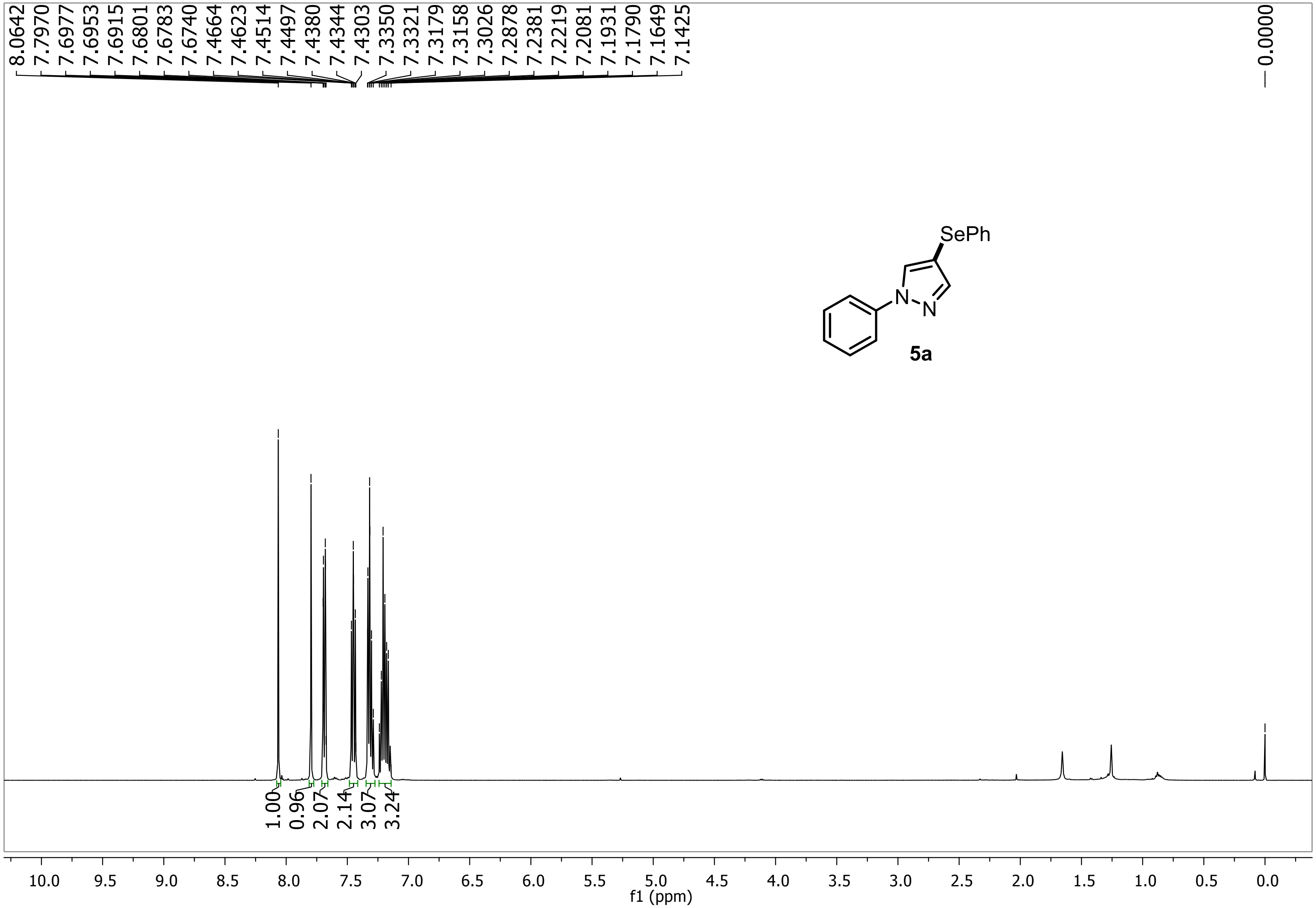

Figure S1: ^{1}H NMR (500 MHz, $CDCl_3$) spectrum of compound **5a**.

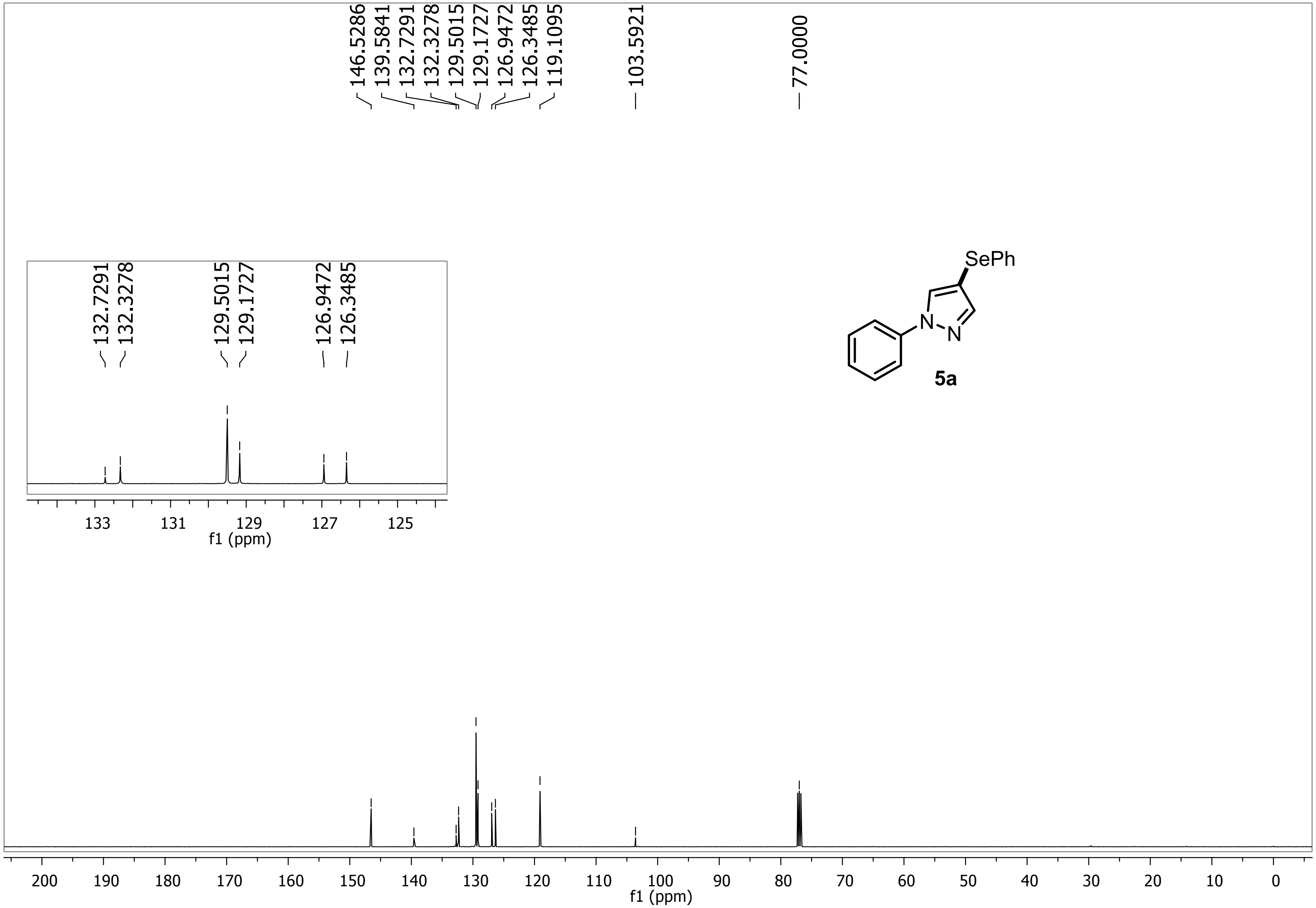

5a

Figure S2: $^{13}C\{^{1}H\}$ NMR (125 MHz, $CDCl_3$) spectrum of compound **5a**.

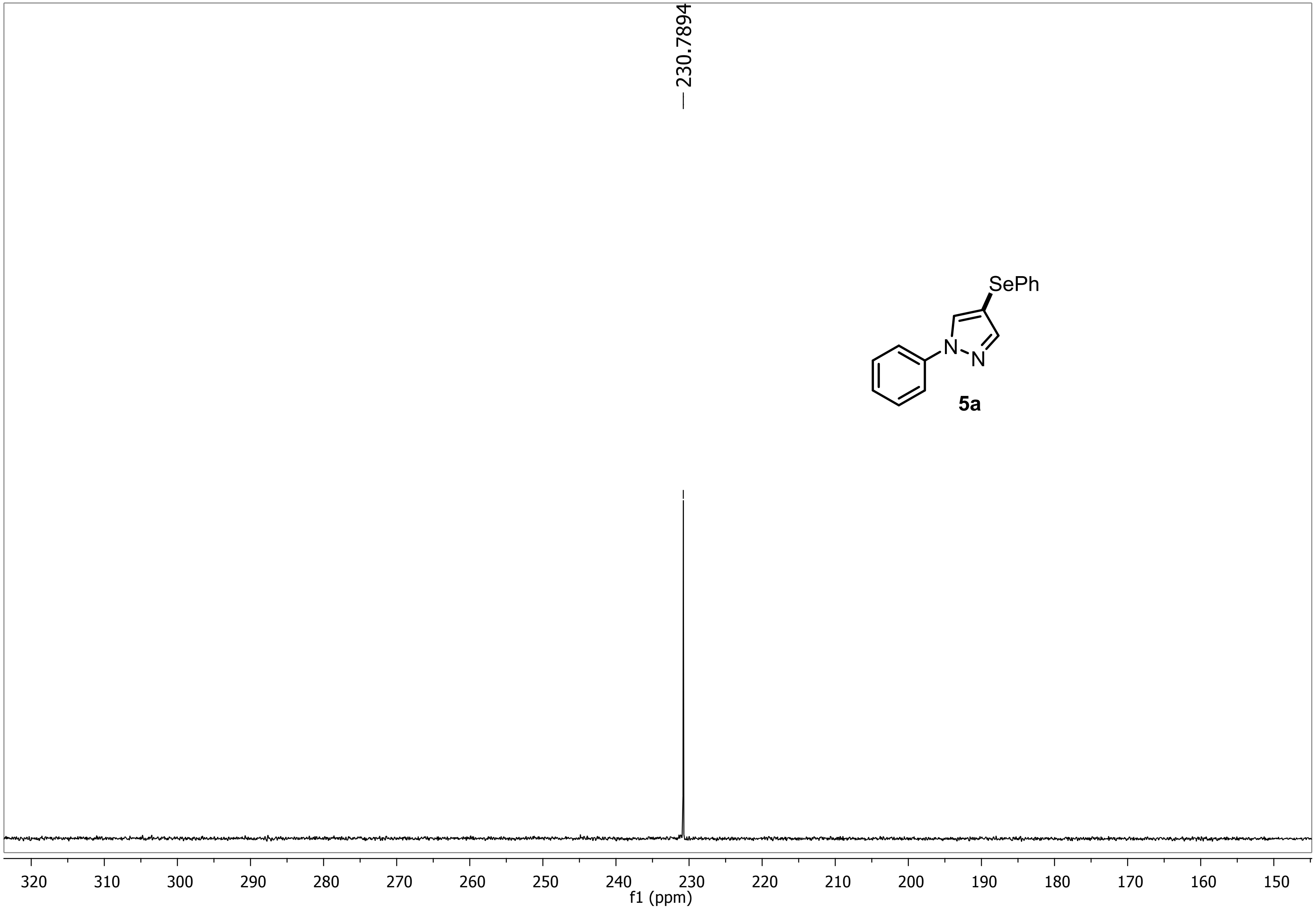

Figure S3: $^{77}Se\{^{1}H\}$ NMR (95 MHz, $CDCl_3$) spectrum of compound **5a**.

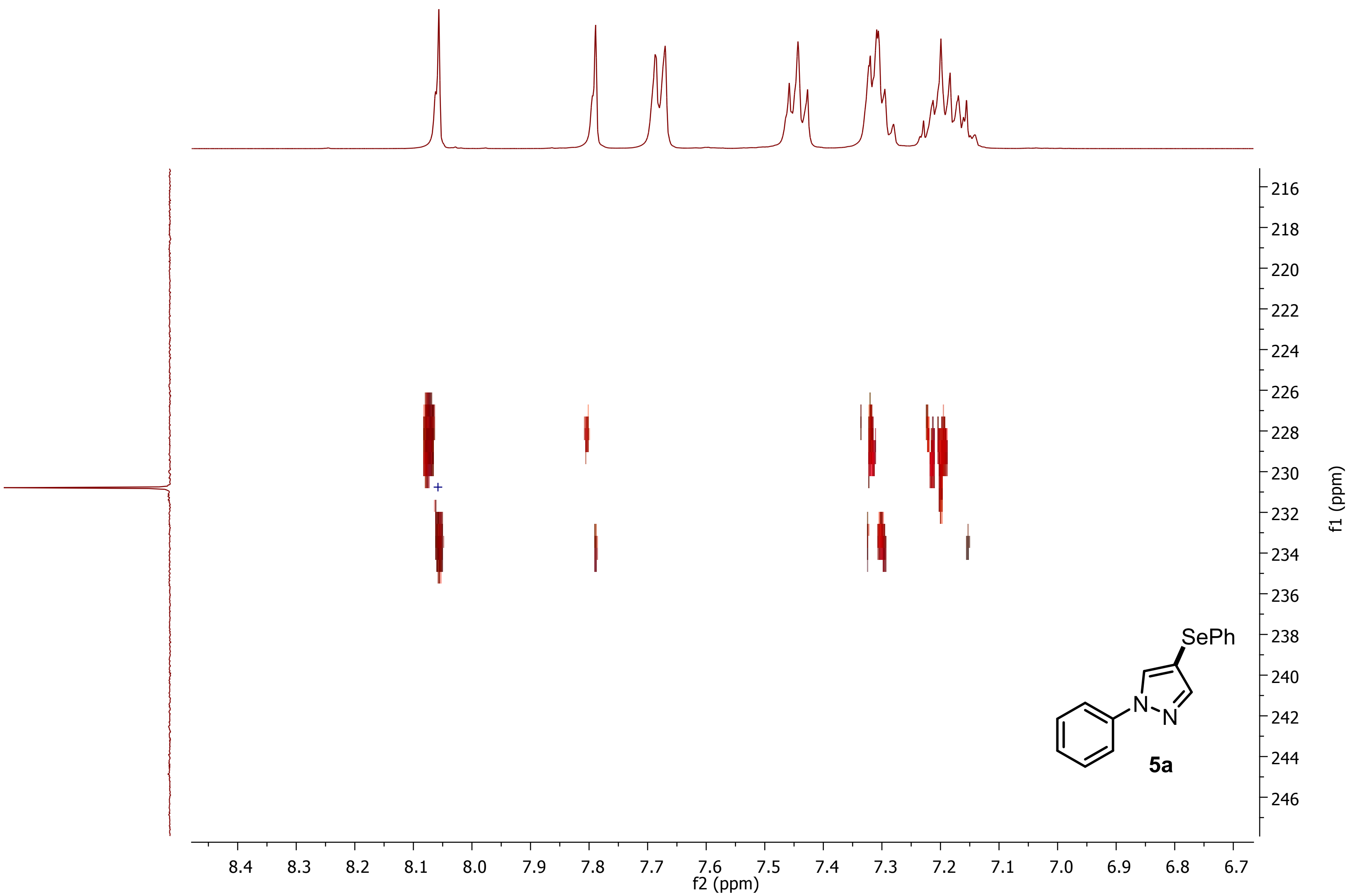

Figure S4: ^{1}H-^{77}Se HMBC NMR (500 MHz, $CDCl_3$) spectrum of compound **5a**.

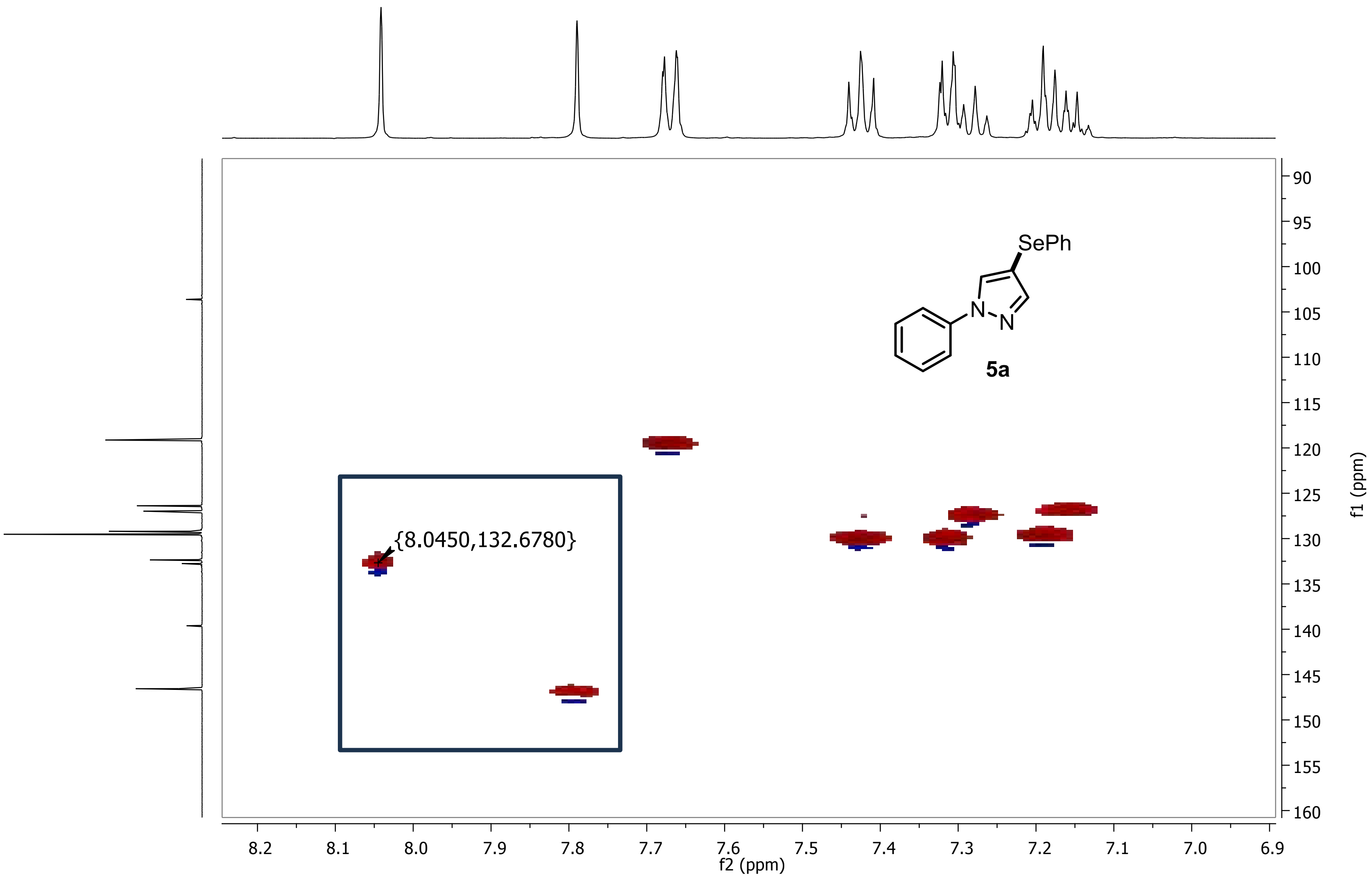

Figure S5: ^{1}H-^{13}C HSQC NMR (500 MHz, $CDCl_3$) spectrum of compound **5a**.

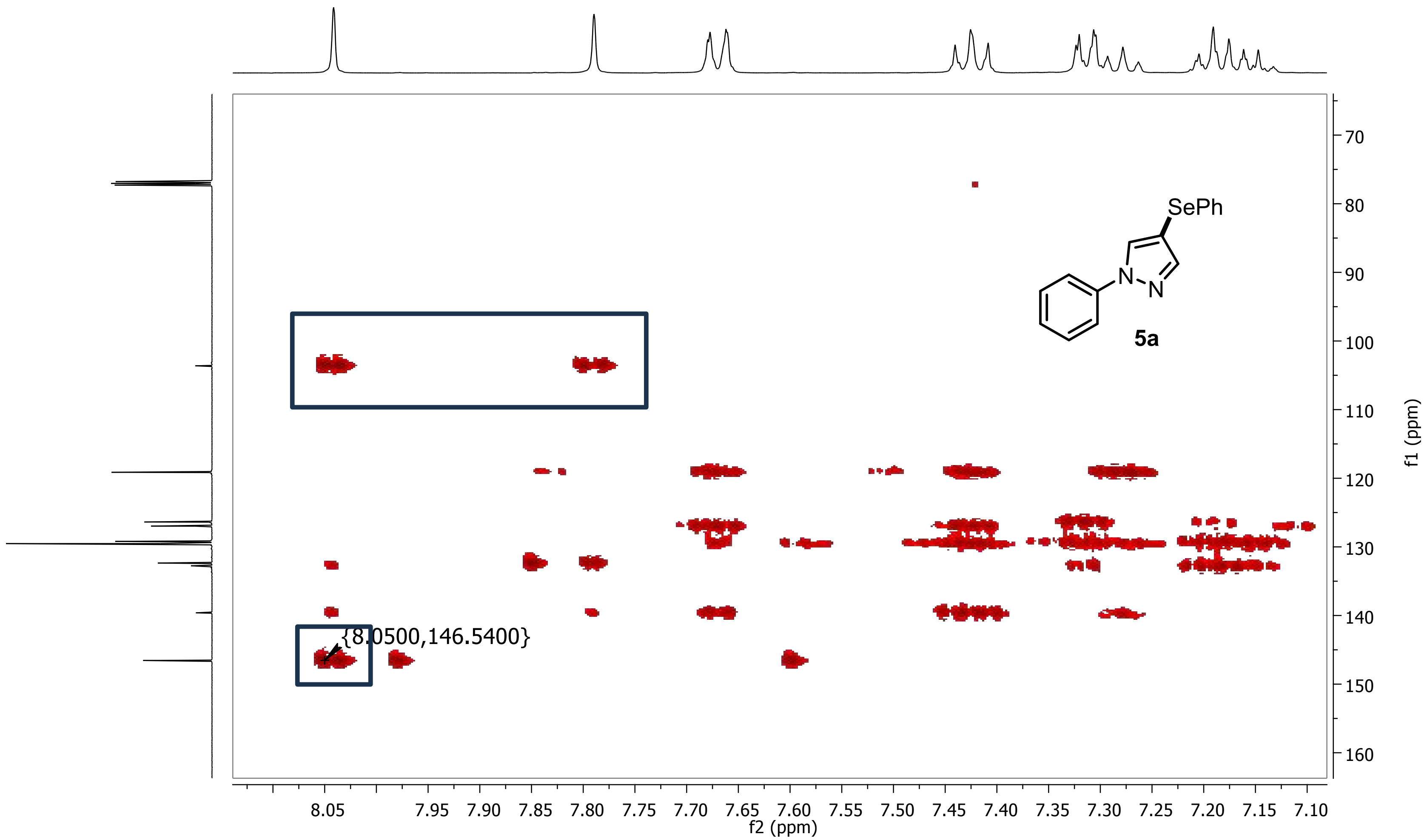

Figure S6: ^{1}H-^{13}C HMBC NMR (500 MHz, $CDCl_3$) spectrum of compound **5a**.

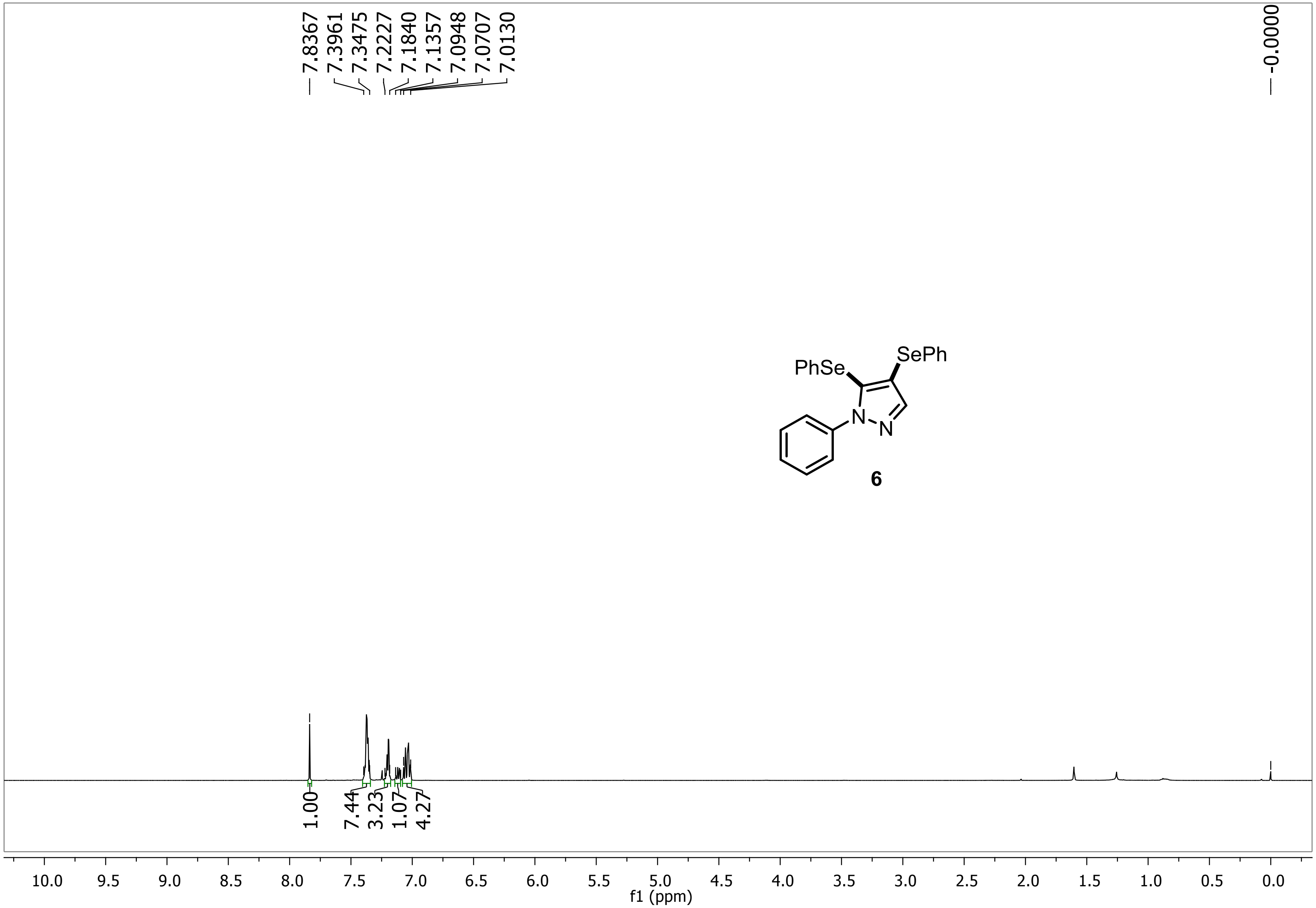

Figure S7: ^{1}H NMR (500 MHz, $CDCl_3$) spectrum of compound **6**.

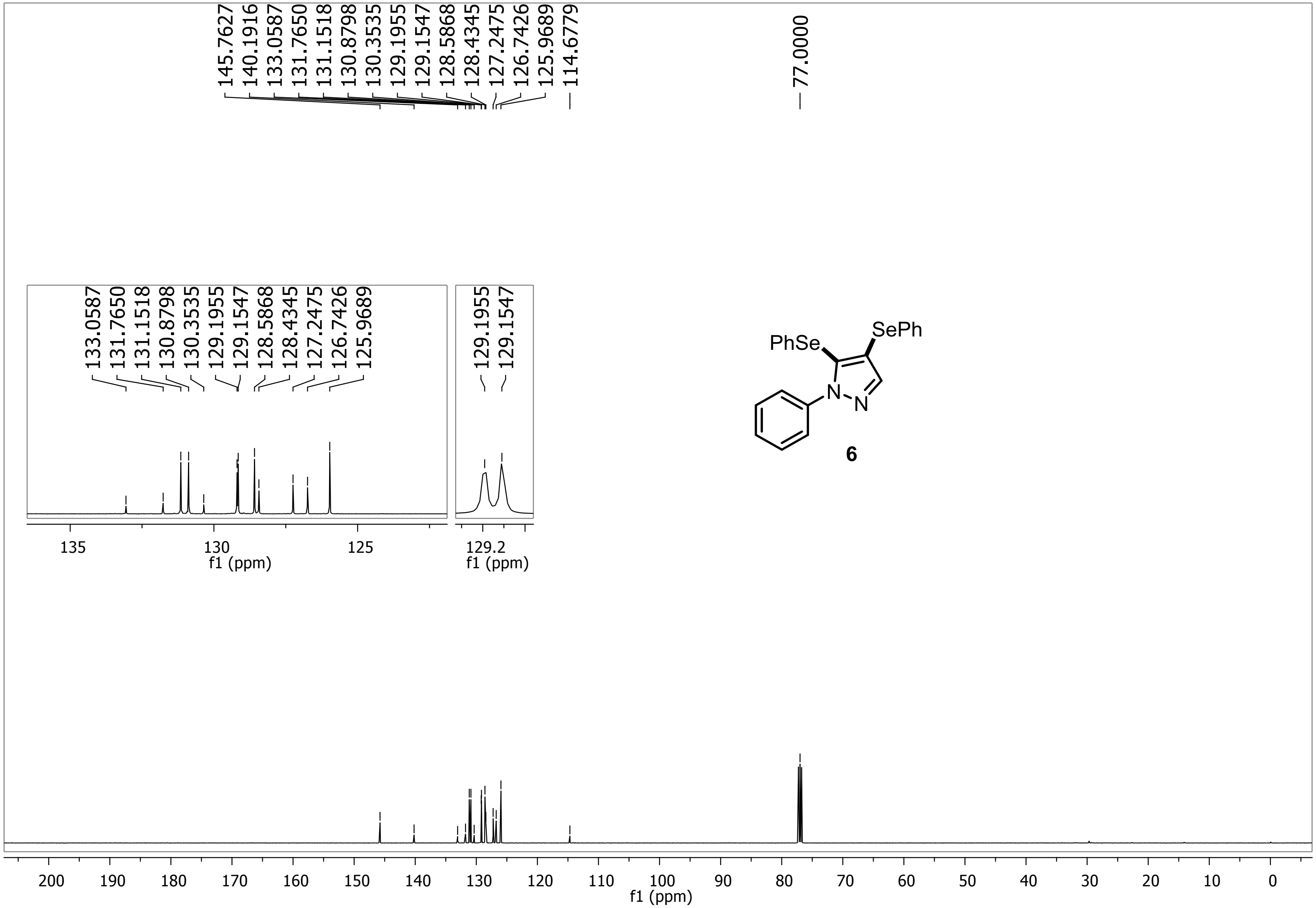

Figure S8: $^{13}C\{^{1}H\}$ NMR (125 MHz, $CDCl_3$) spectrum of compound **6**.

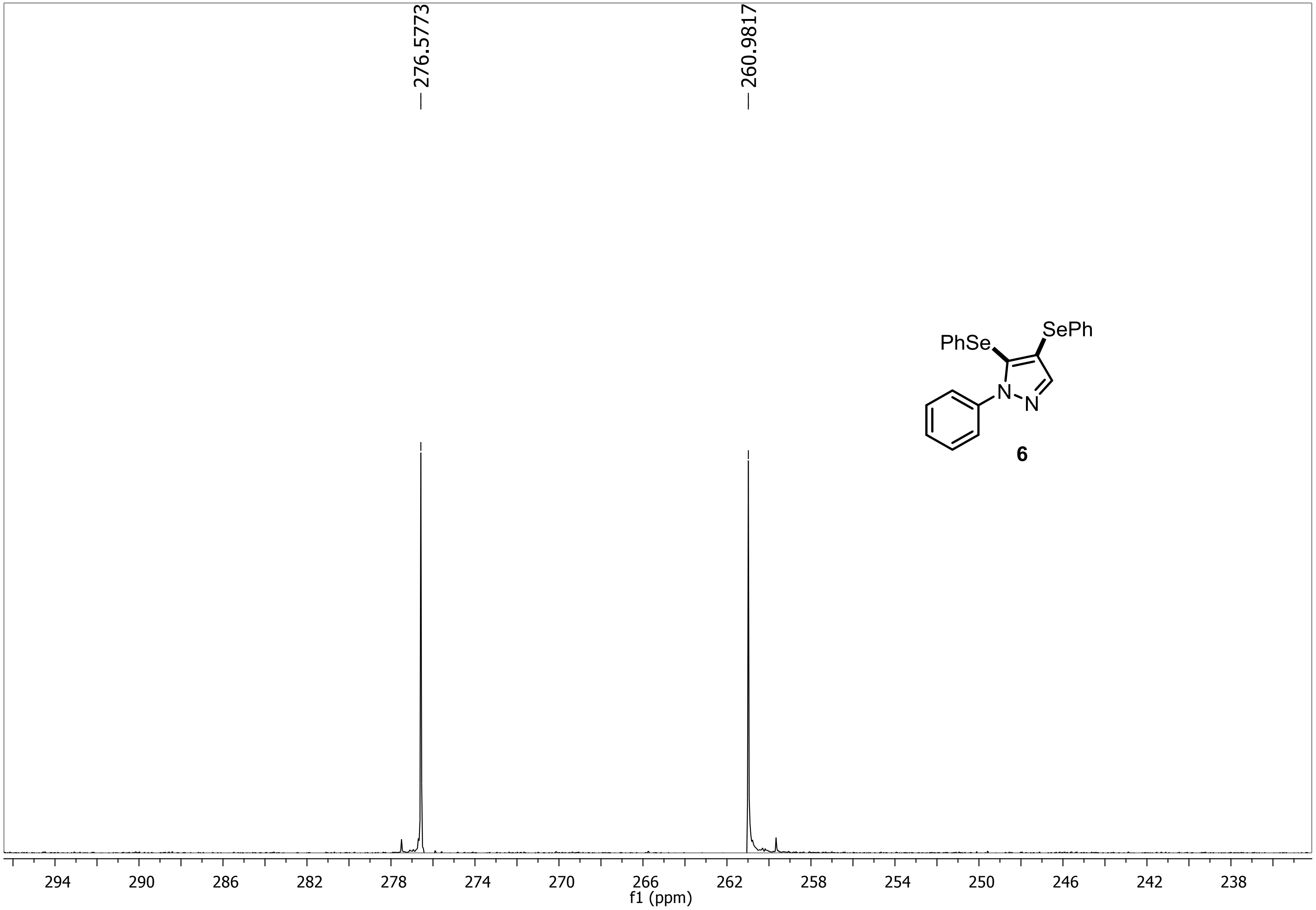

Figure S9: $^{77}Se\{^1H\}$ NMR (95 MHz, $CDCl_3$) spectrum of compound **6**.

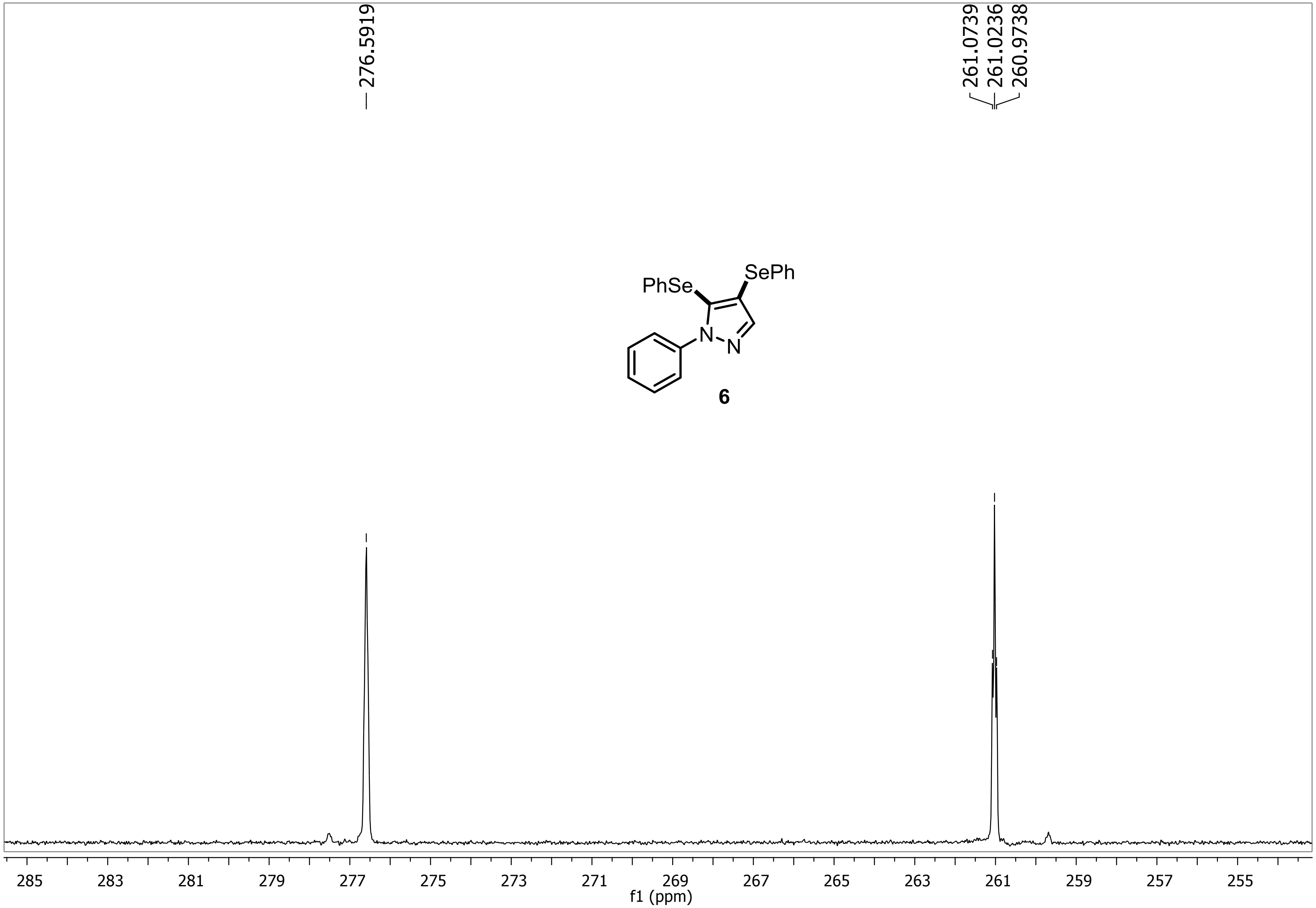

Figure S10: ^{77}Se NMR (95 MHz, $CDCl_3$) spectrum of compound **6**.

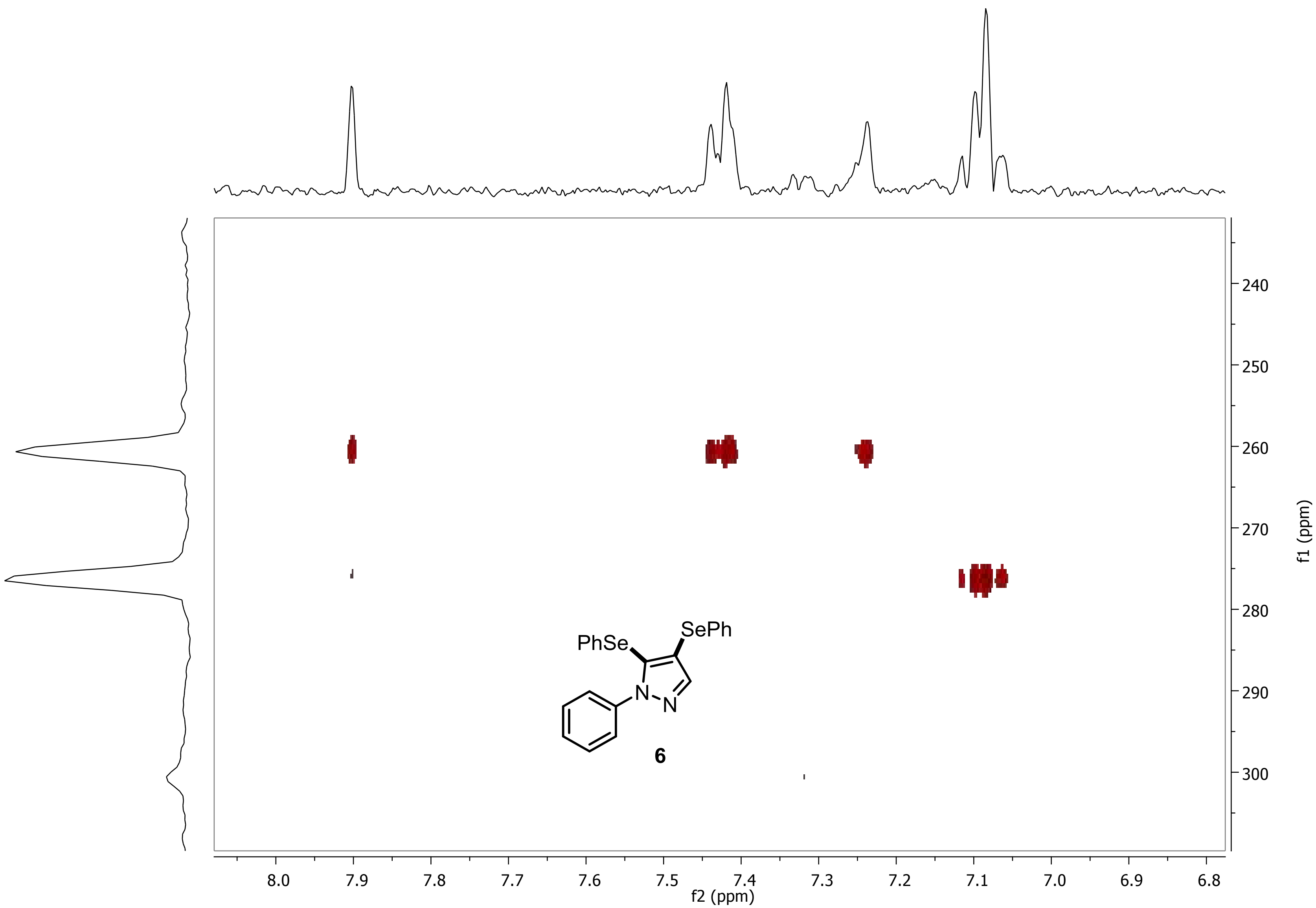

Figure S11: ^{1}H-^{77}Se HMBC NMR (500 MHz, $CDCl_3$) spectrum of compound **6**.

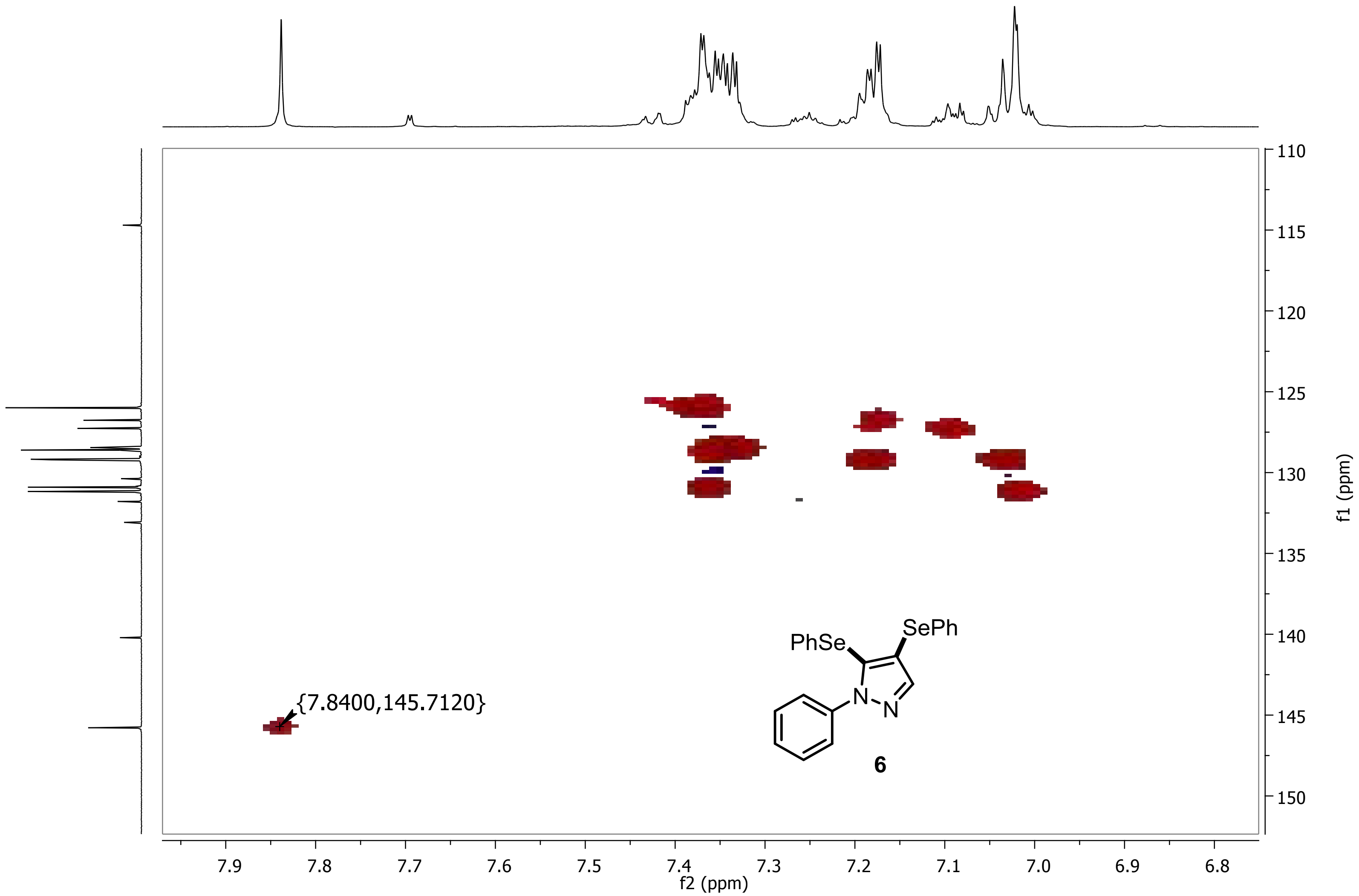

Figure S12: ^{1}H-^{13}C HSQC NMR (500 MHz, $CDCl_3$) spectrum of compound **6**.

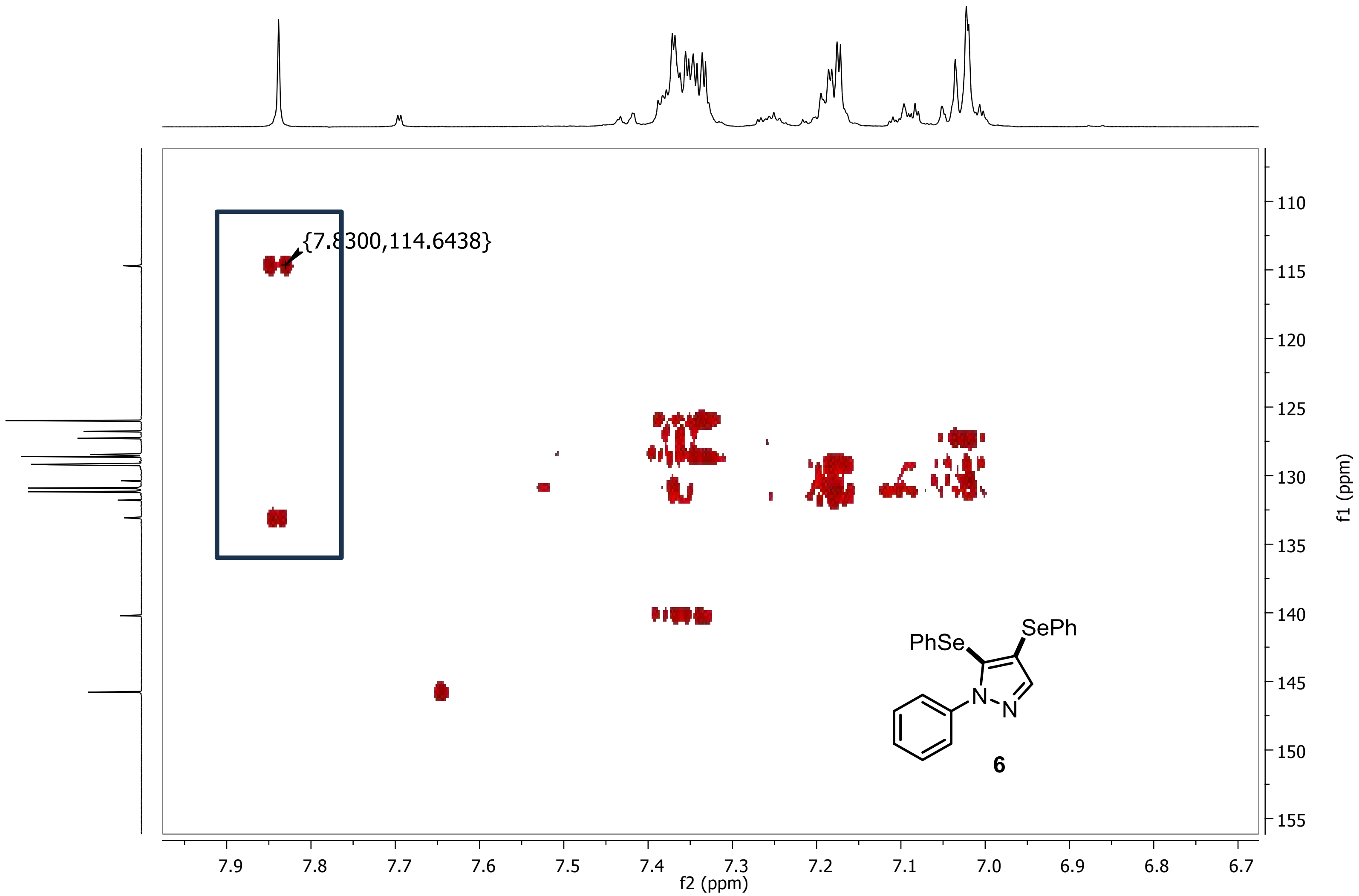

Figure S13: ^{1}H-^{13}C HMBC NMR (500 MHz, $CDCl_3$) spectrum of compound **6**.

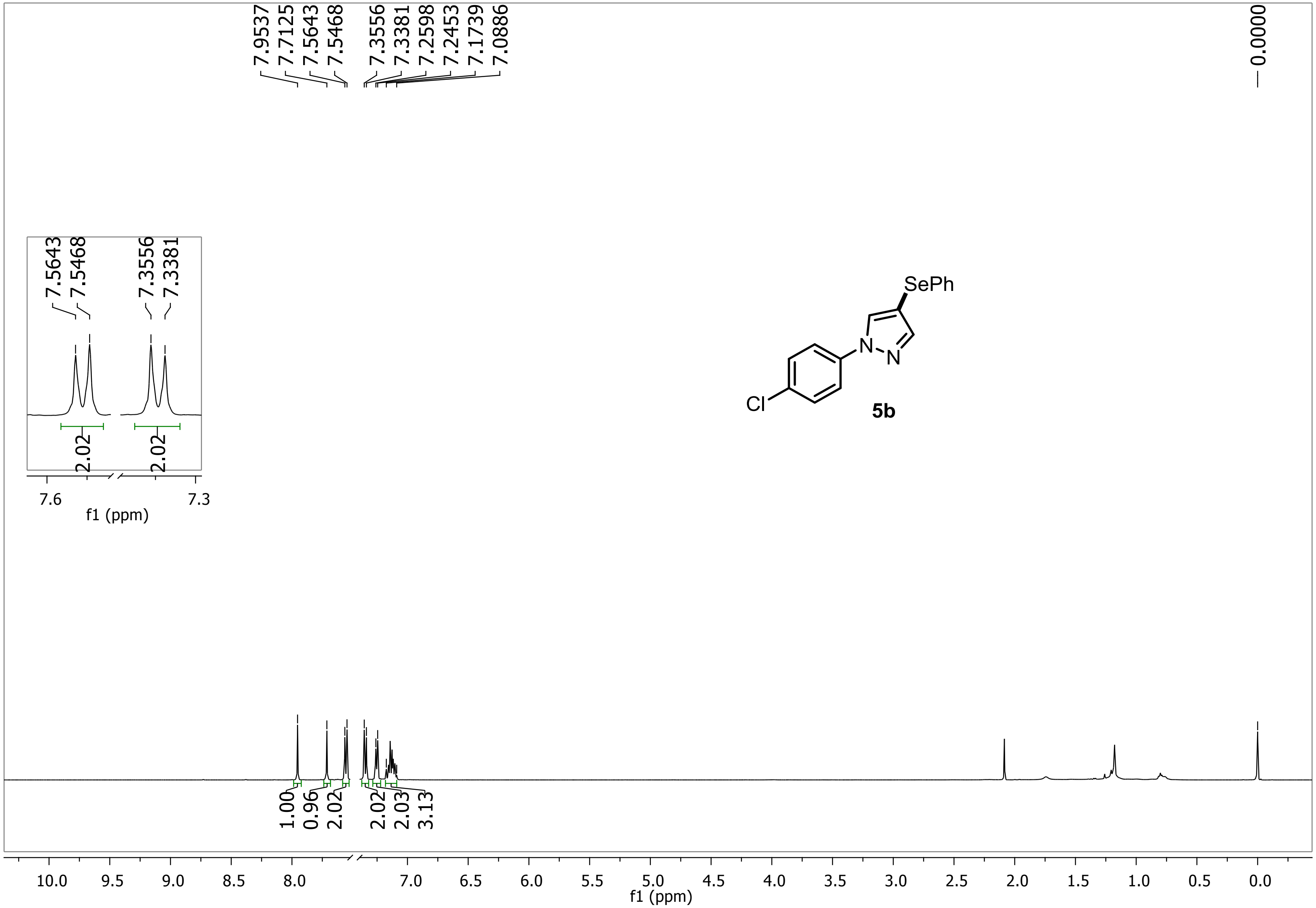

Figure S14: ^{1}H NMR (500 MHz, $CDCl_3$) spectrum of compound **5b**.

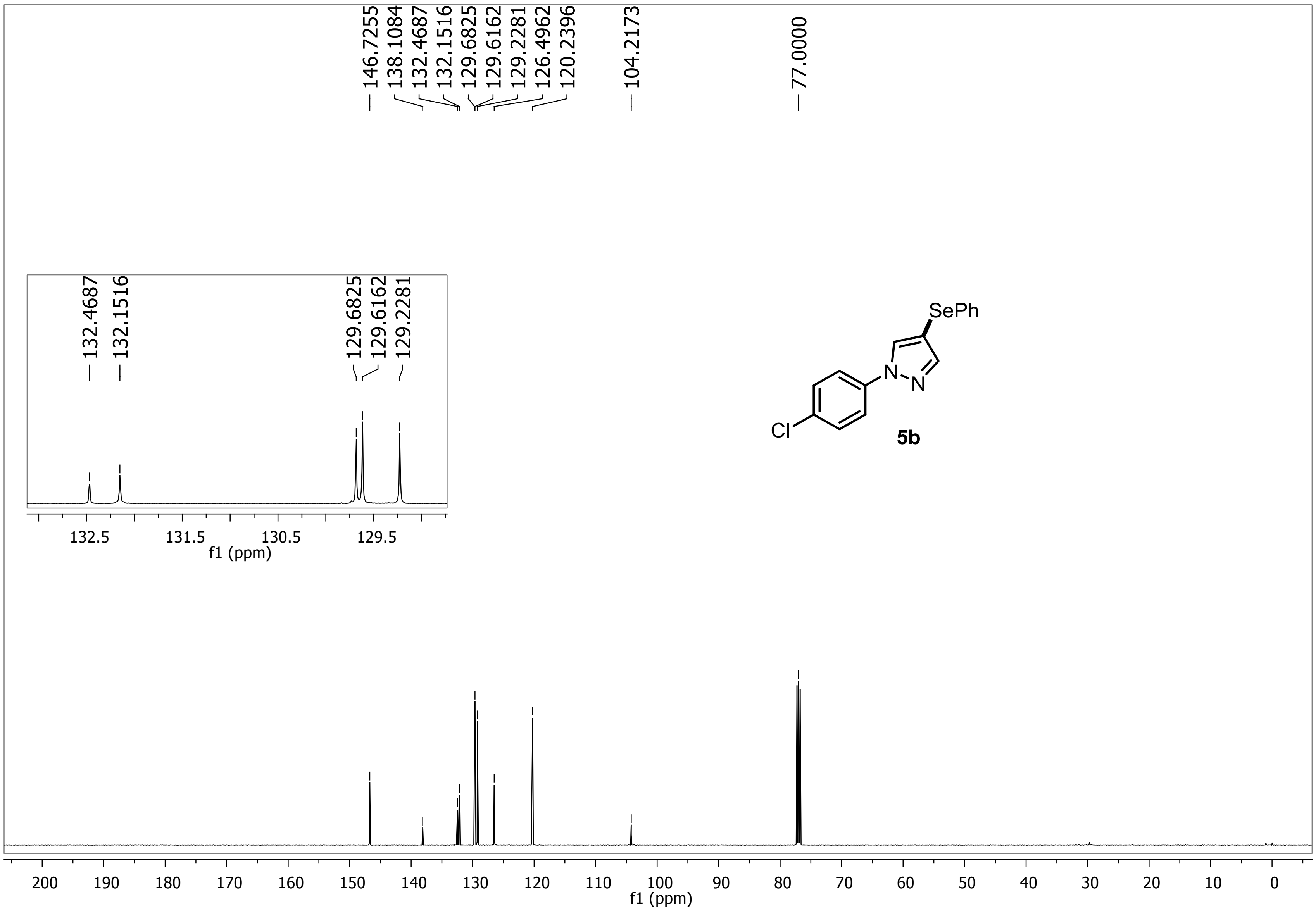

Figure S15: $^{13}C\{^{1}H\}$ NMR (125 MHz, $CDCl_3$) spectrum of compound **5b**.

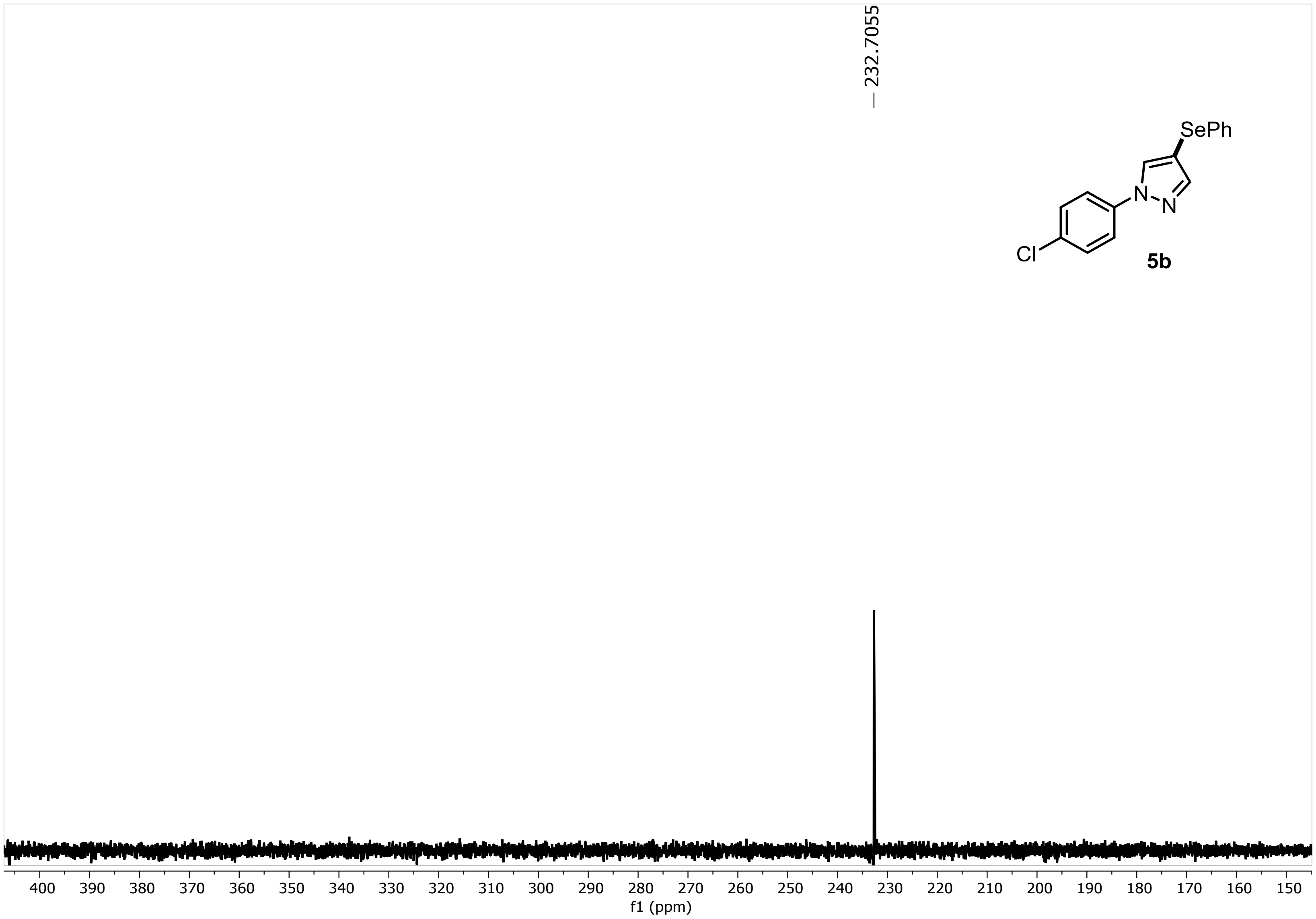

Figure S16: ^{77}Se NMR (95 MHz, $CDCl_3$) spectrum of compound **5b**.

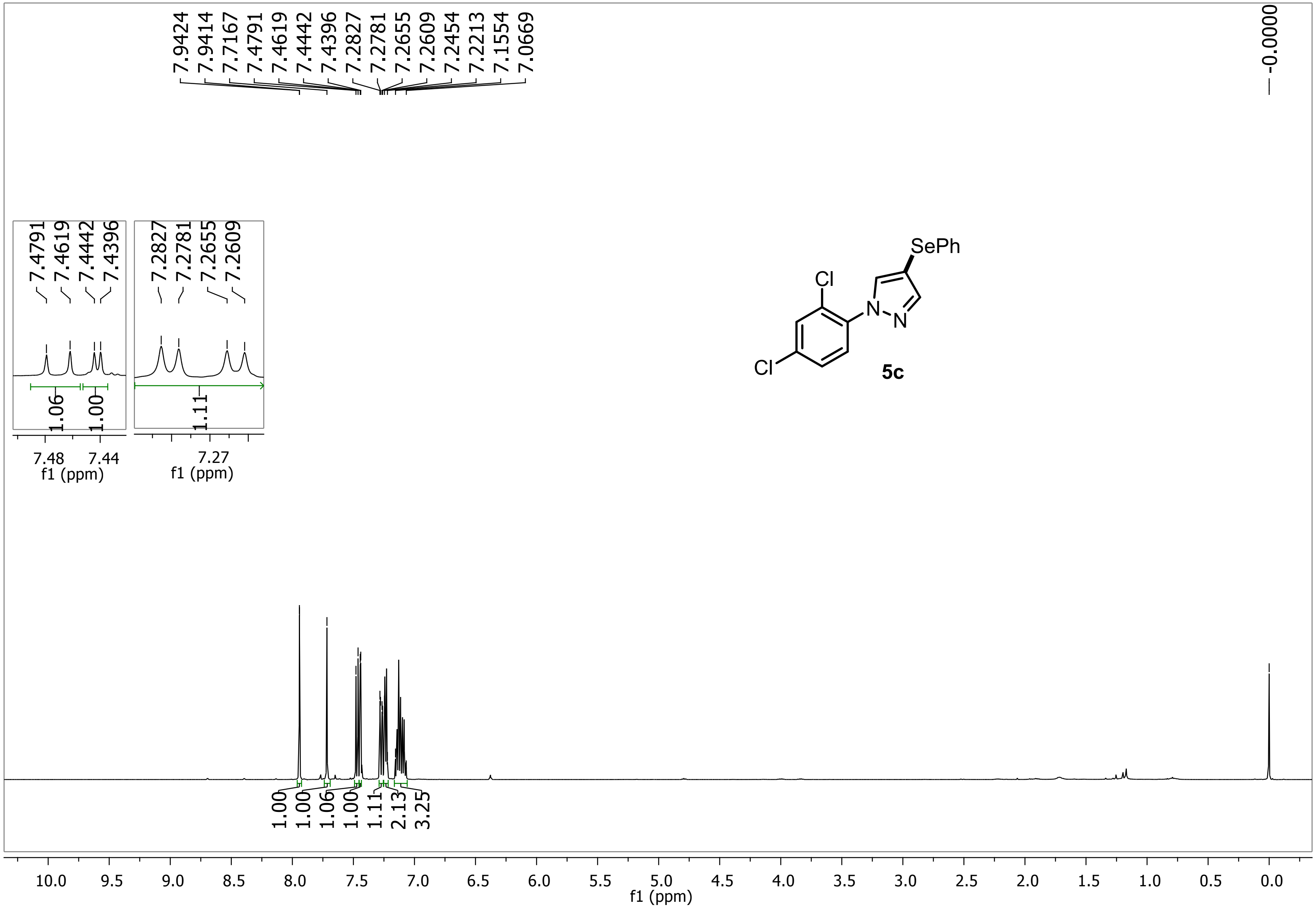

Figure S17: ^{1}H NMR (500 MHz, $CDCl_3$) spectrum of compound **5c**.

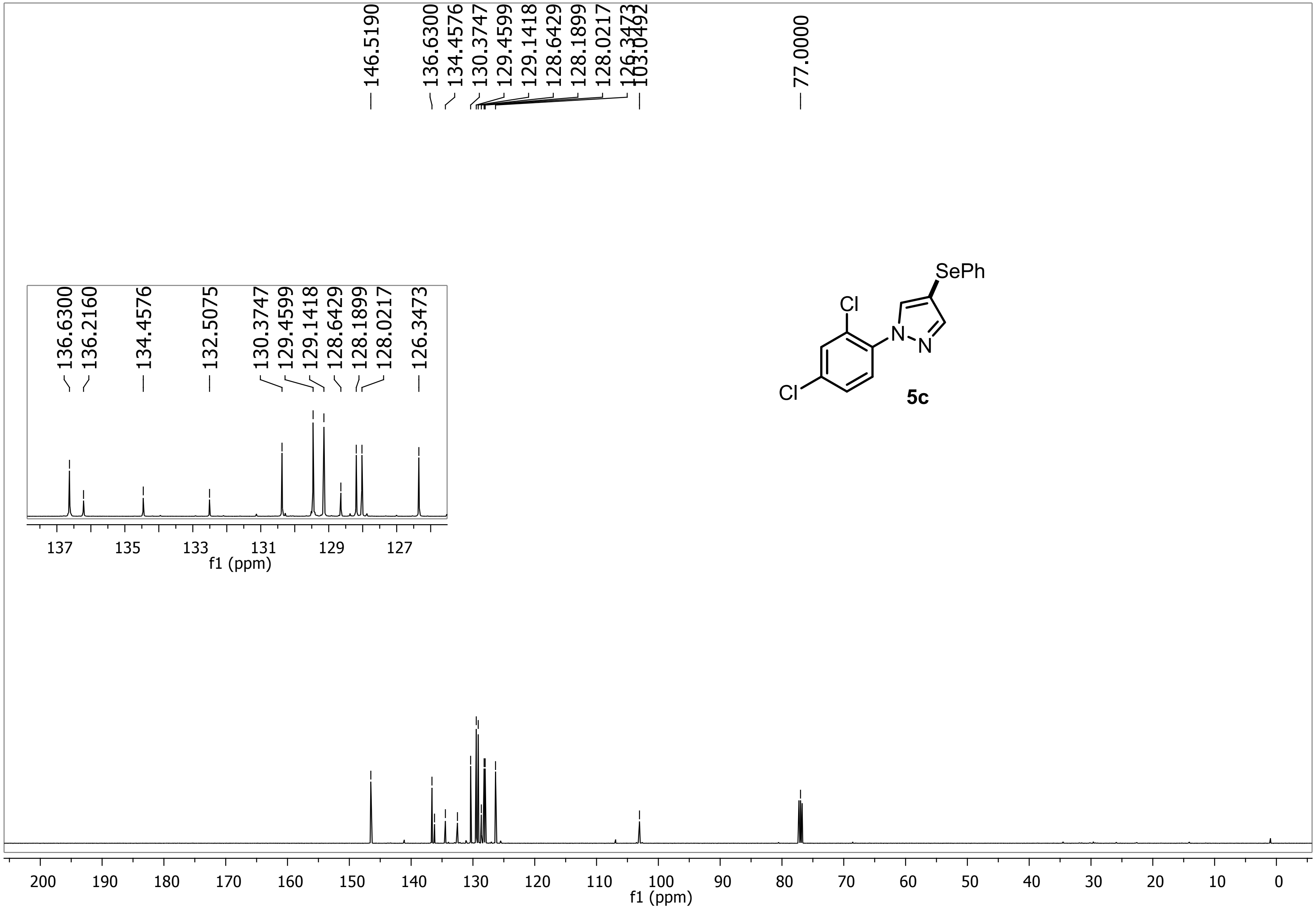

5c

Figure S18: $^{13}C\{^{1}H\}$ NMR (125 MHz, $CDCl_3$) spectrum of compound **5c**.

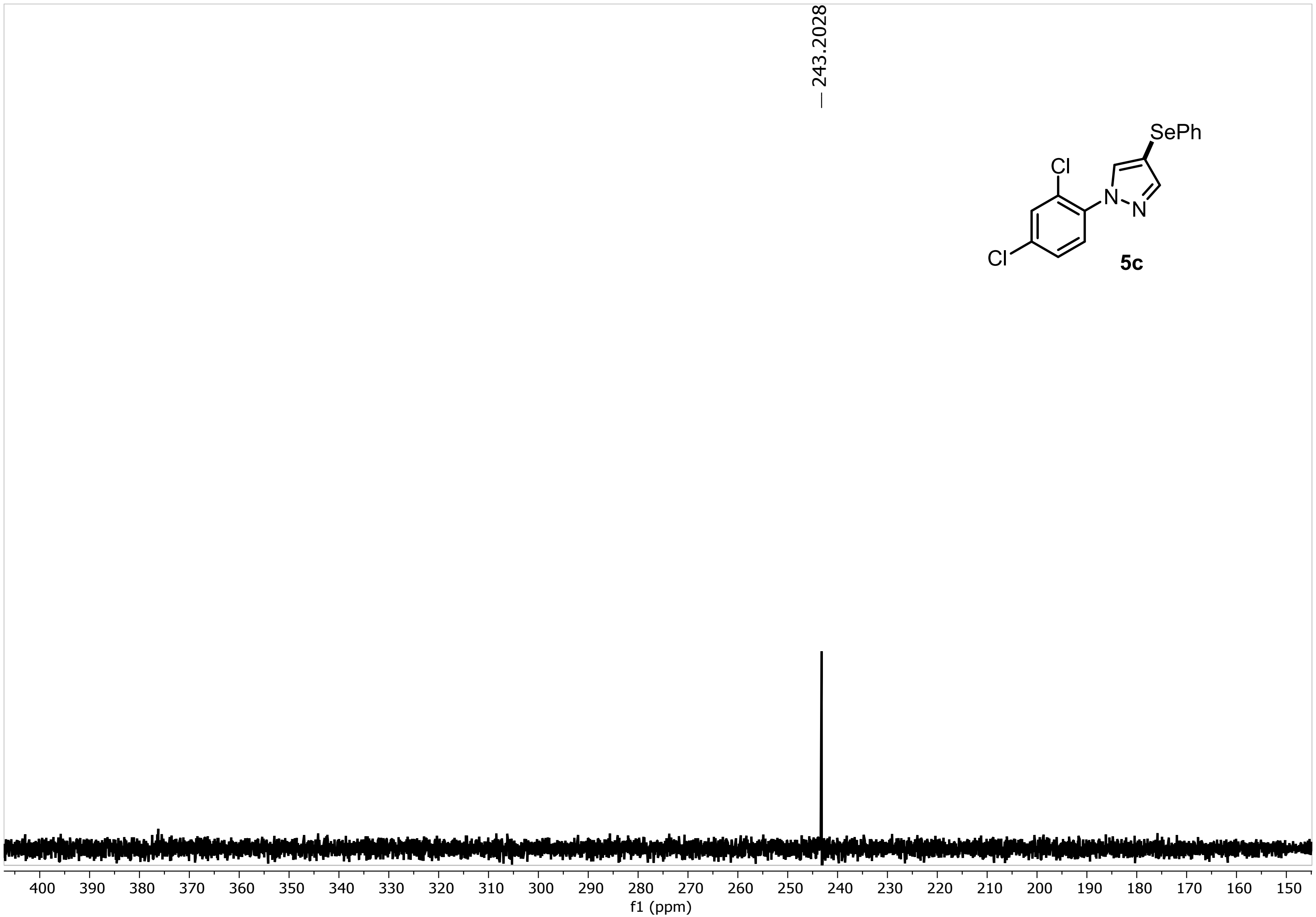

5c

Figure S19: ^{77}Se NMR (95 MHz, $CDCl_3$) spectrum of compound **5c**.

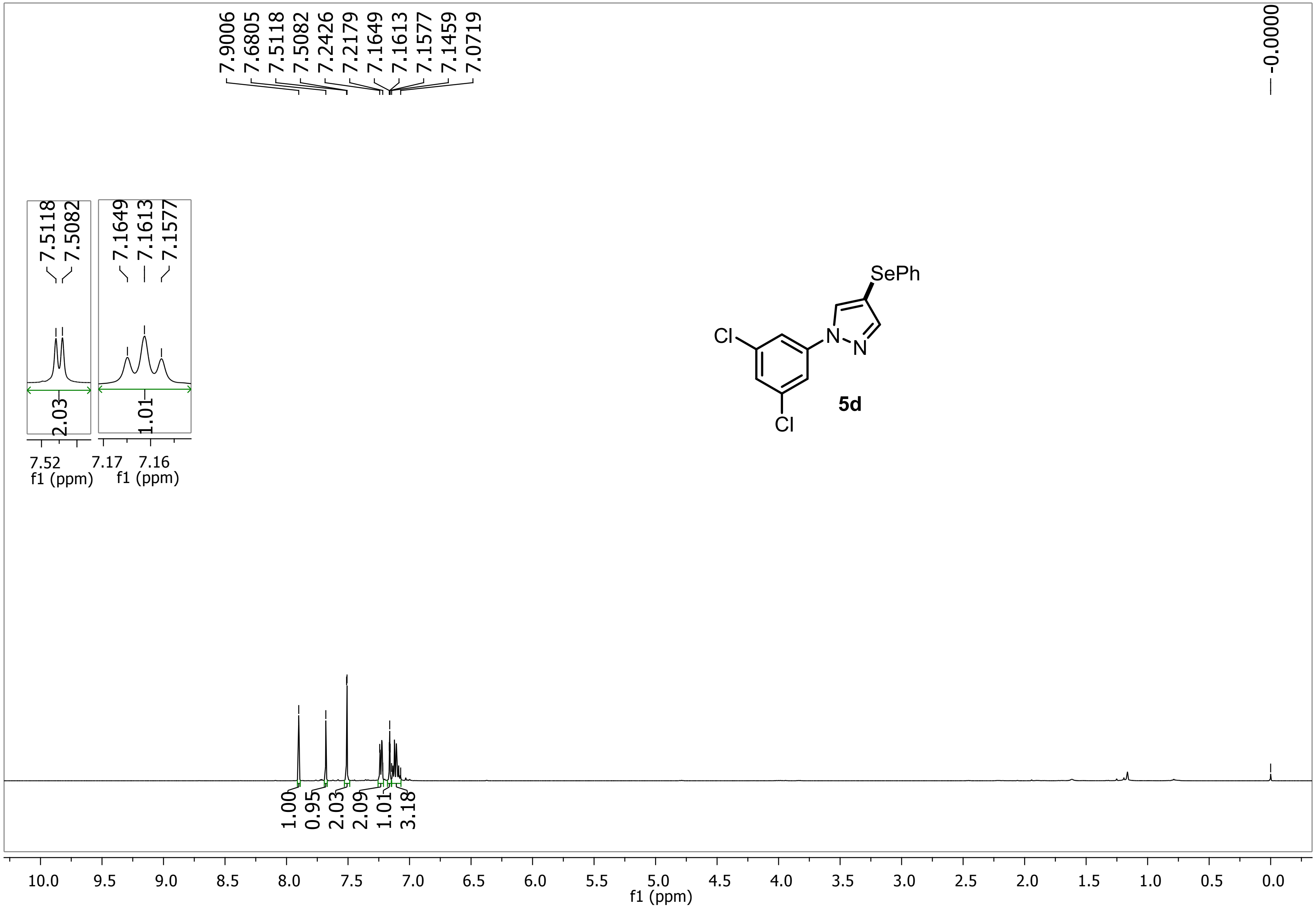

Figure S20: ^{1}H NMR (500 MHz, $CDCl_3$) spectrum of compound **5d**.

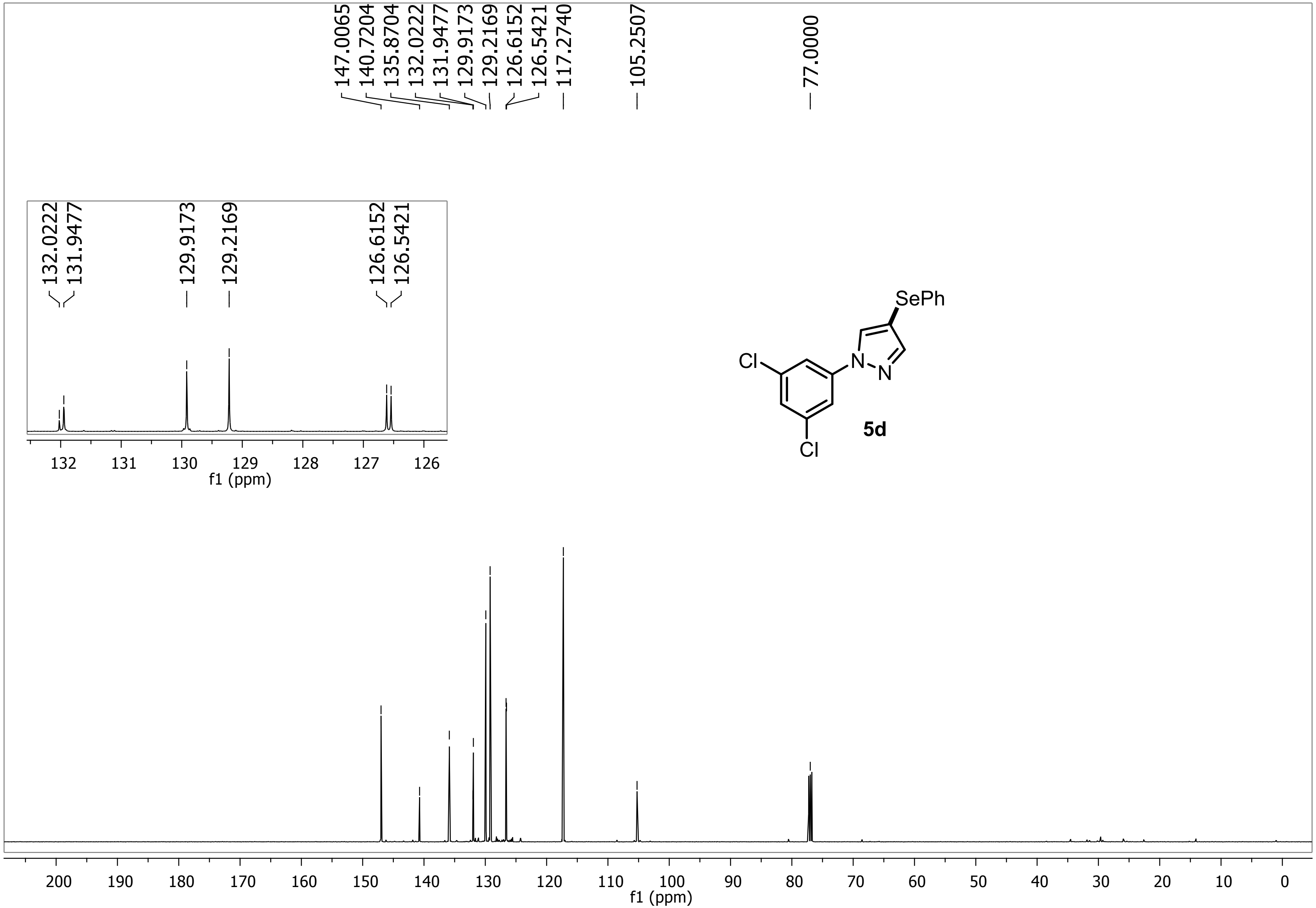

Figure S21: $^{13}C\{^1H\}$ NMR (125 MHz, $CDCl_3$) spectrum of compound **5d**.

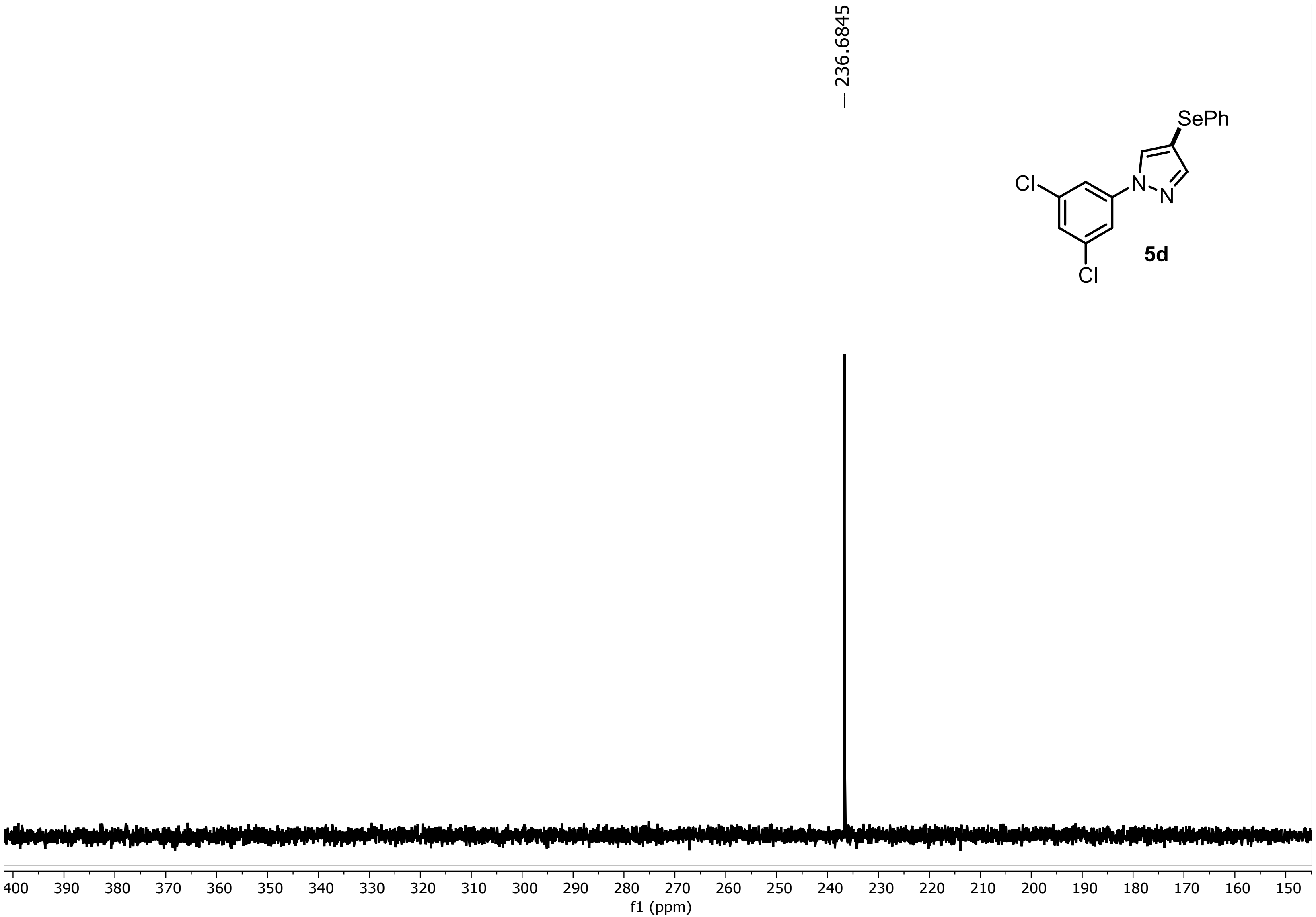

Figure S22: ^{77}Se NMR (95 MHz, $CDCl_3$) spectrum of compound **5d**.

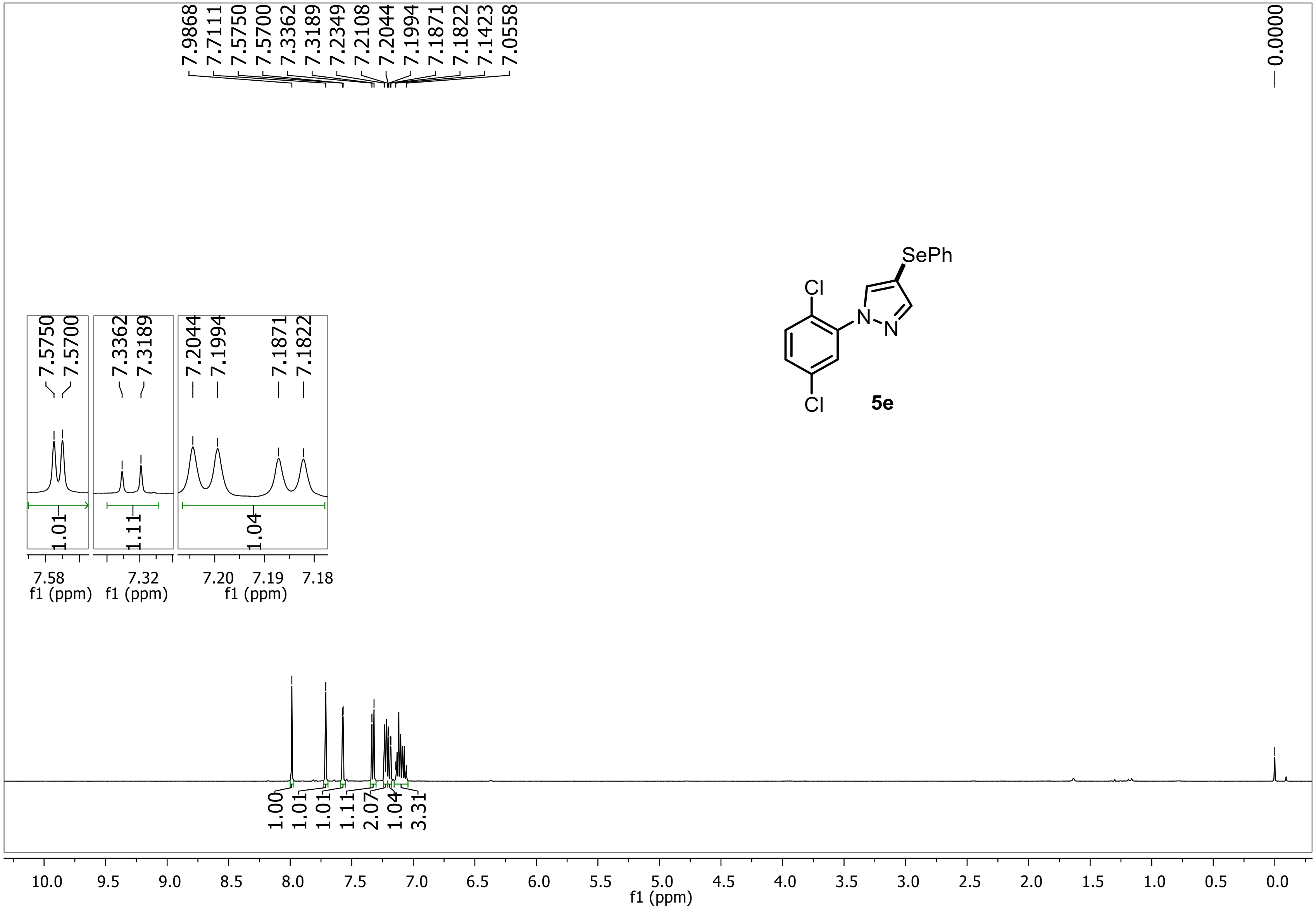

Figure S23: ^{1}H NMR (500 MHz, $CDCl_3$) spectrum of compound **5e**.

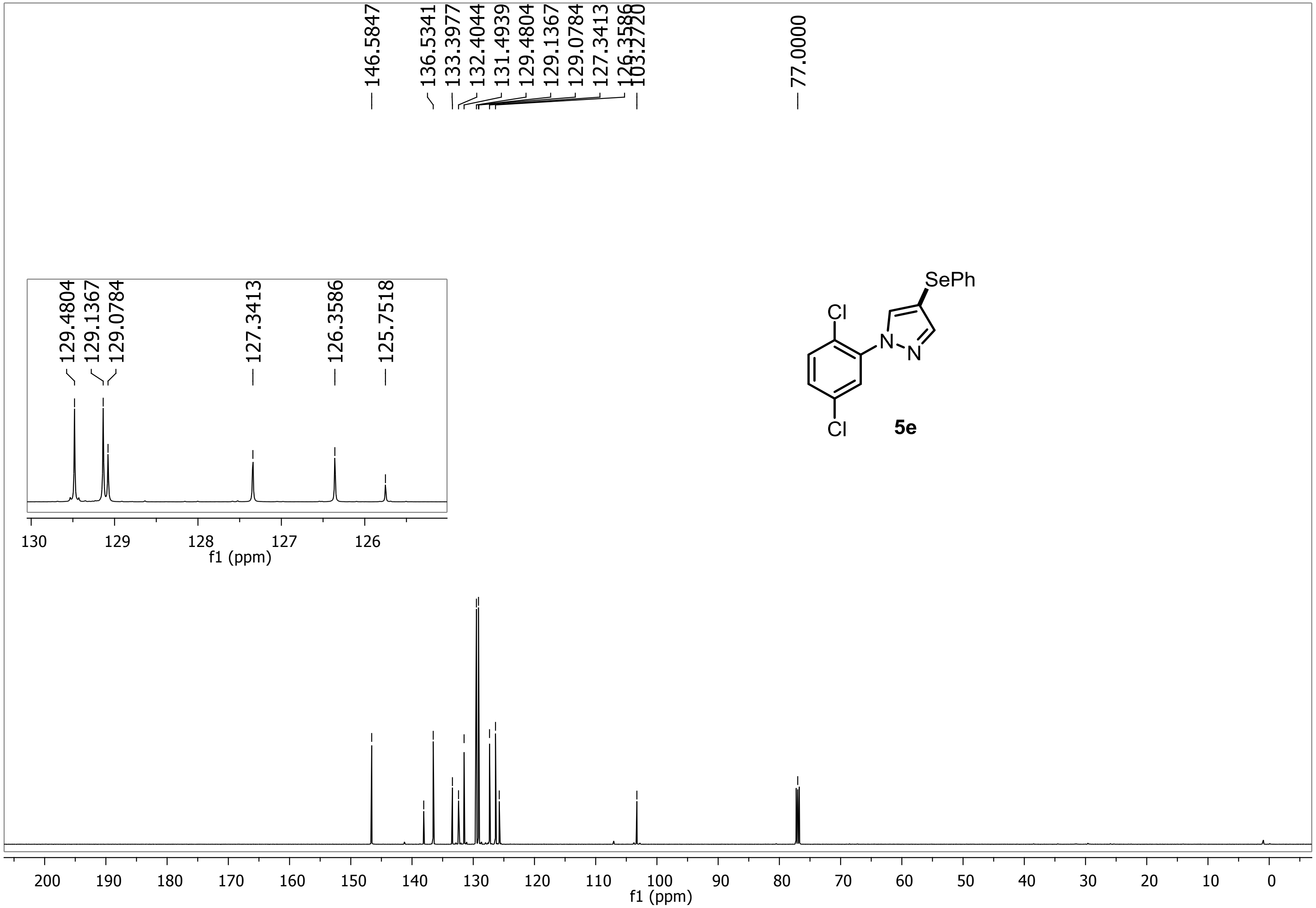

Figure S24: $^{13}C\{^{1}H\}$ NMR (125 MHz, $CDCl_3$) spectrum of compound **5e**.

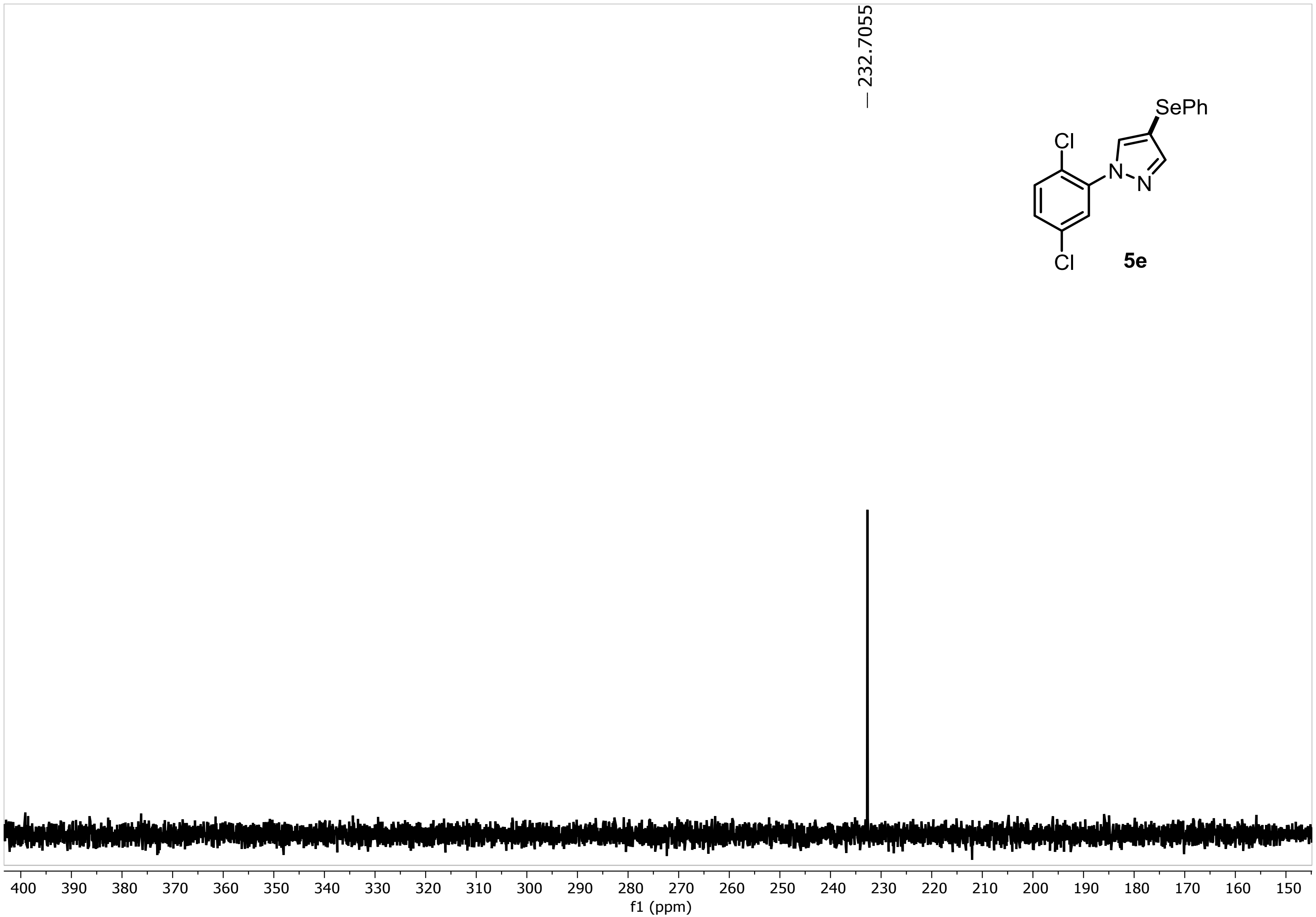

Figure S25: ^{77}Se NMR (95 MHz, $CDCl_3$) spectrum of compound **5e**.

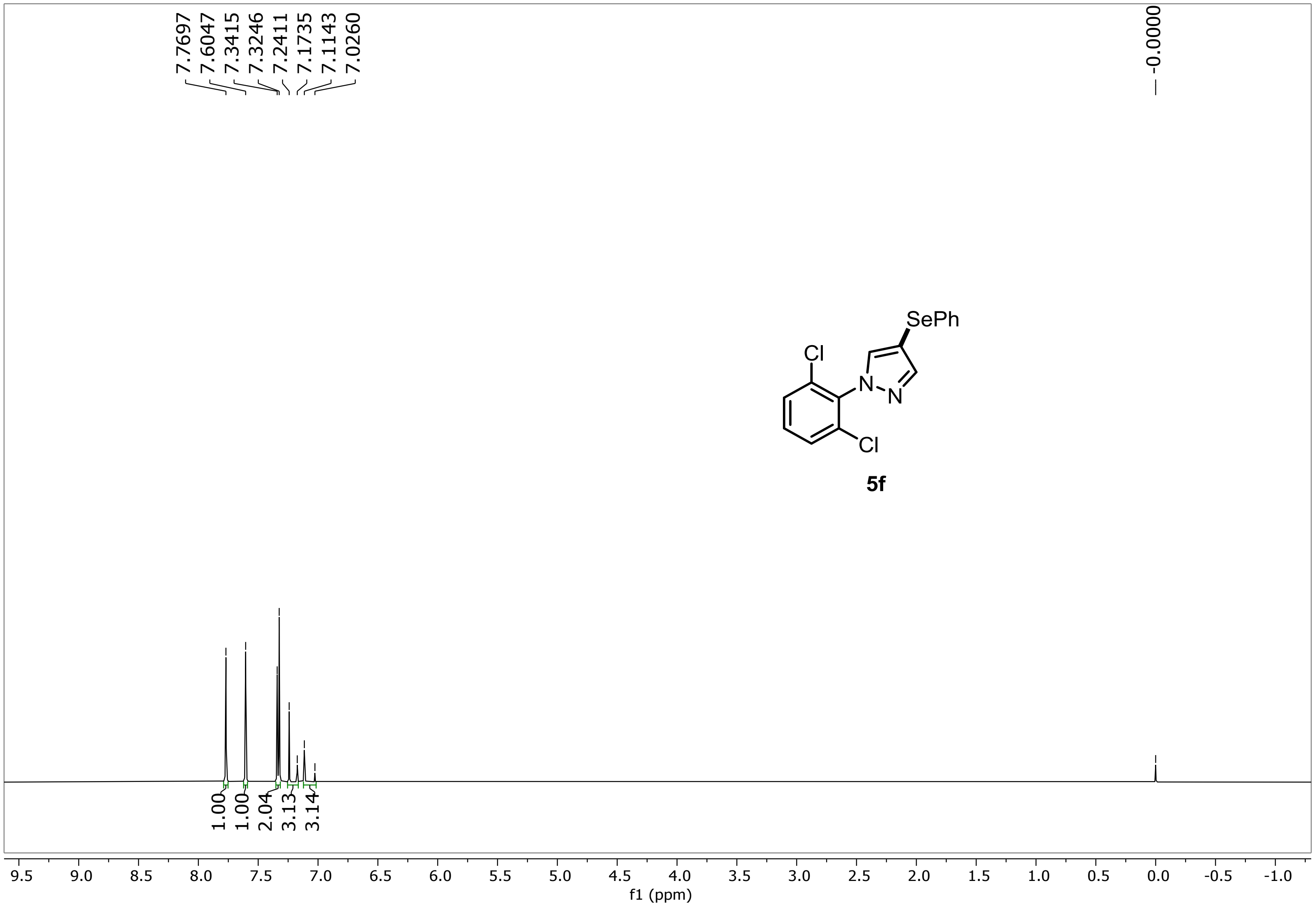

Figure S26: ^{1}H NMR (500 MHz, $CDCl_3$) spectrum of compound **5f**.

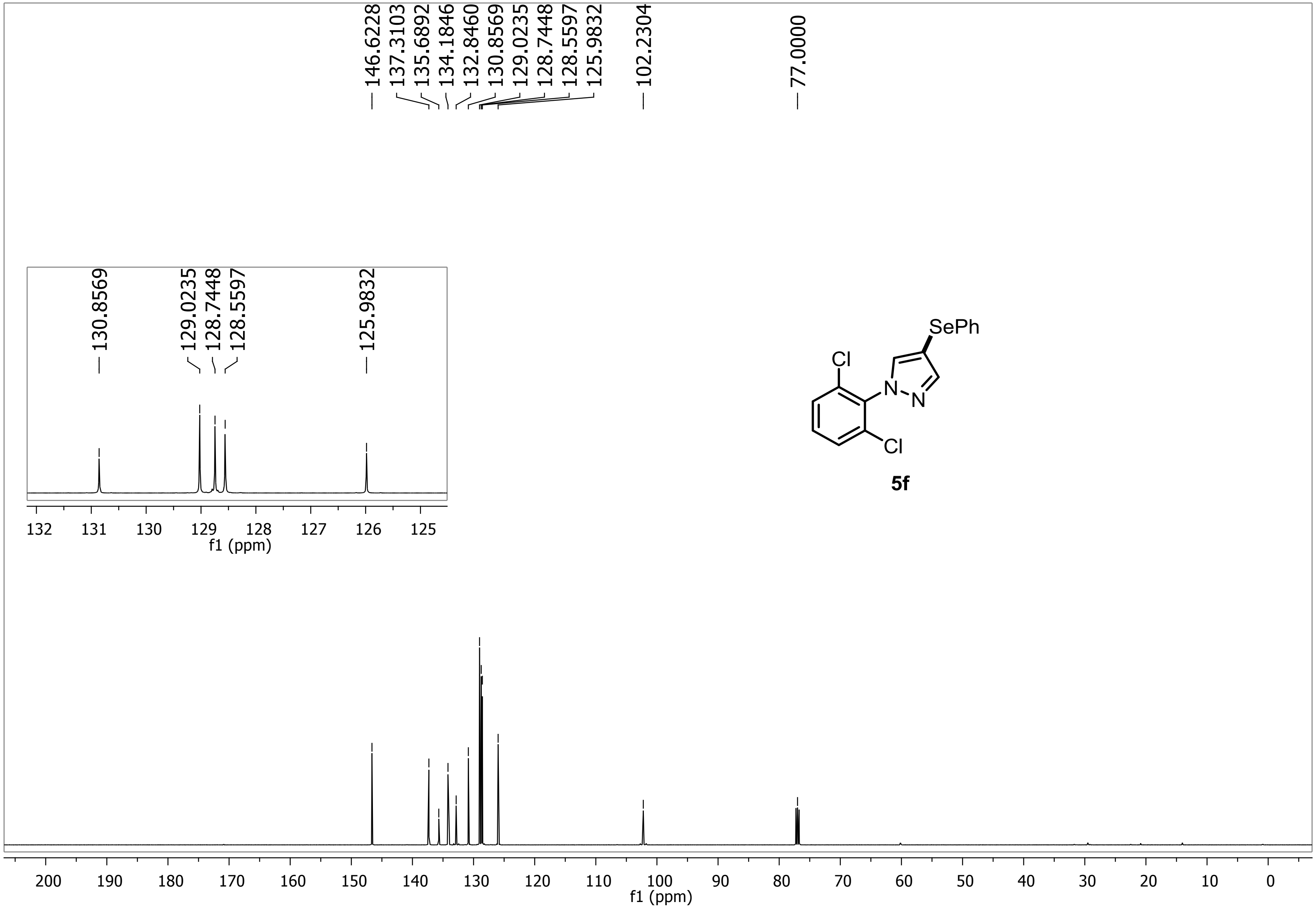

Figure S27: $^{13}C\{^1H\}$ NMR (125 MHz, $CDCl_3$) spectrum of compound **5f**.

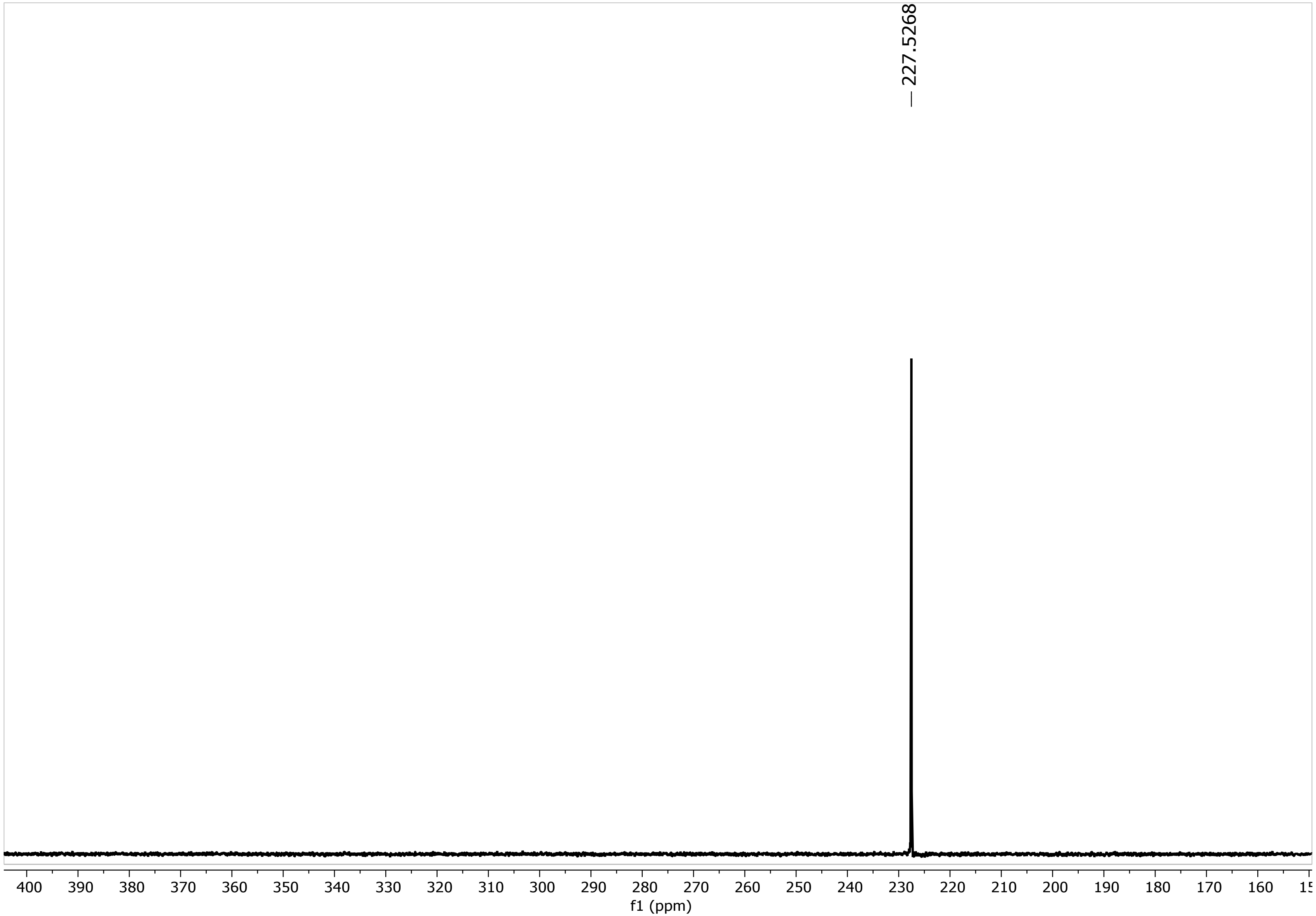

Figure S28: ^{77}Se NMR (95 MHz, $CDCl_3$) spectrum of compound **5f**.

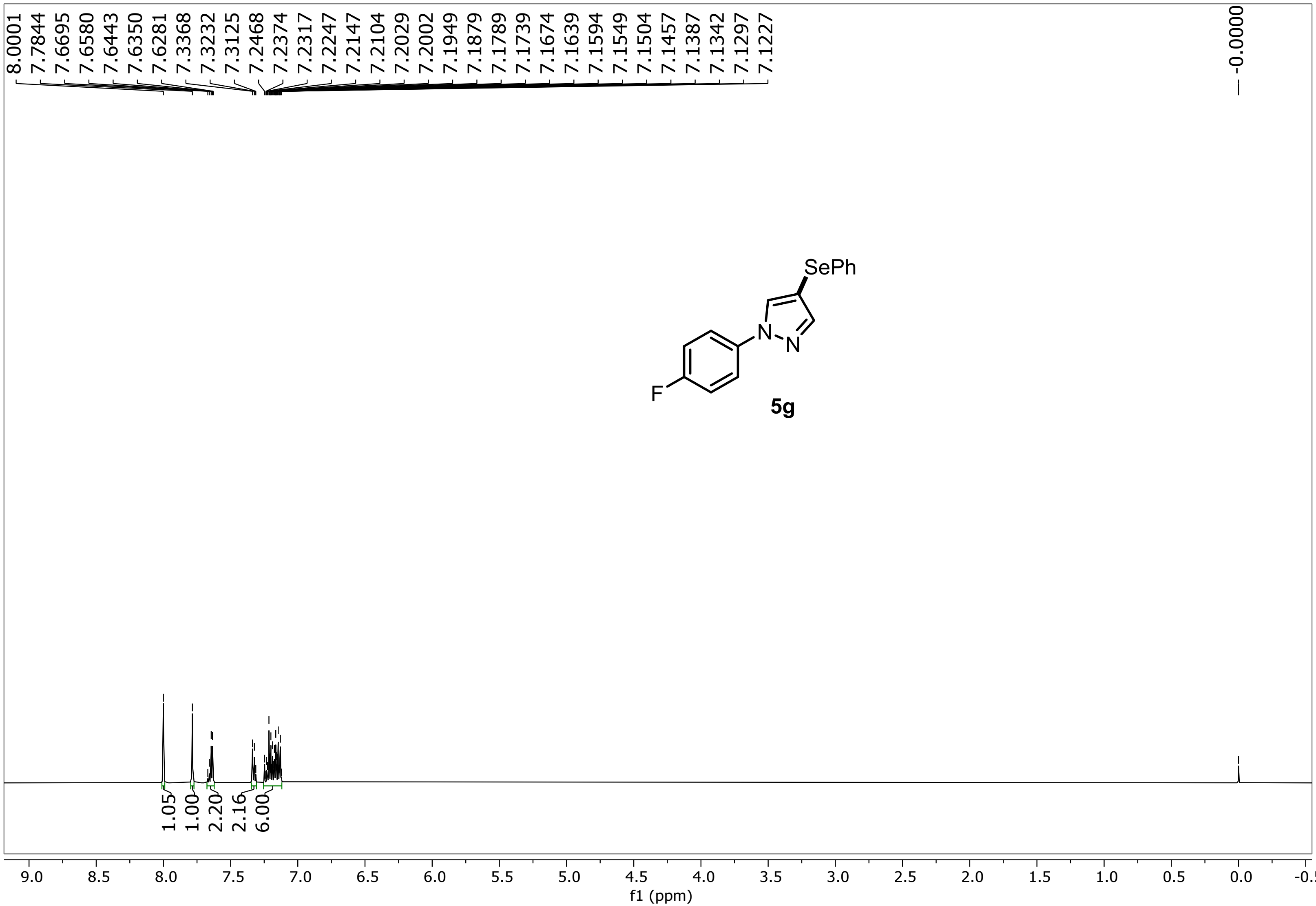

Figure S29: ^{1}H NMR (500 MHz, $CDCl_3$) spectrum of compound **5g**.

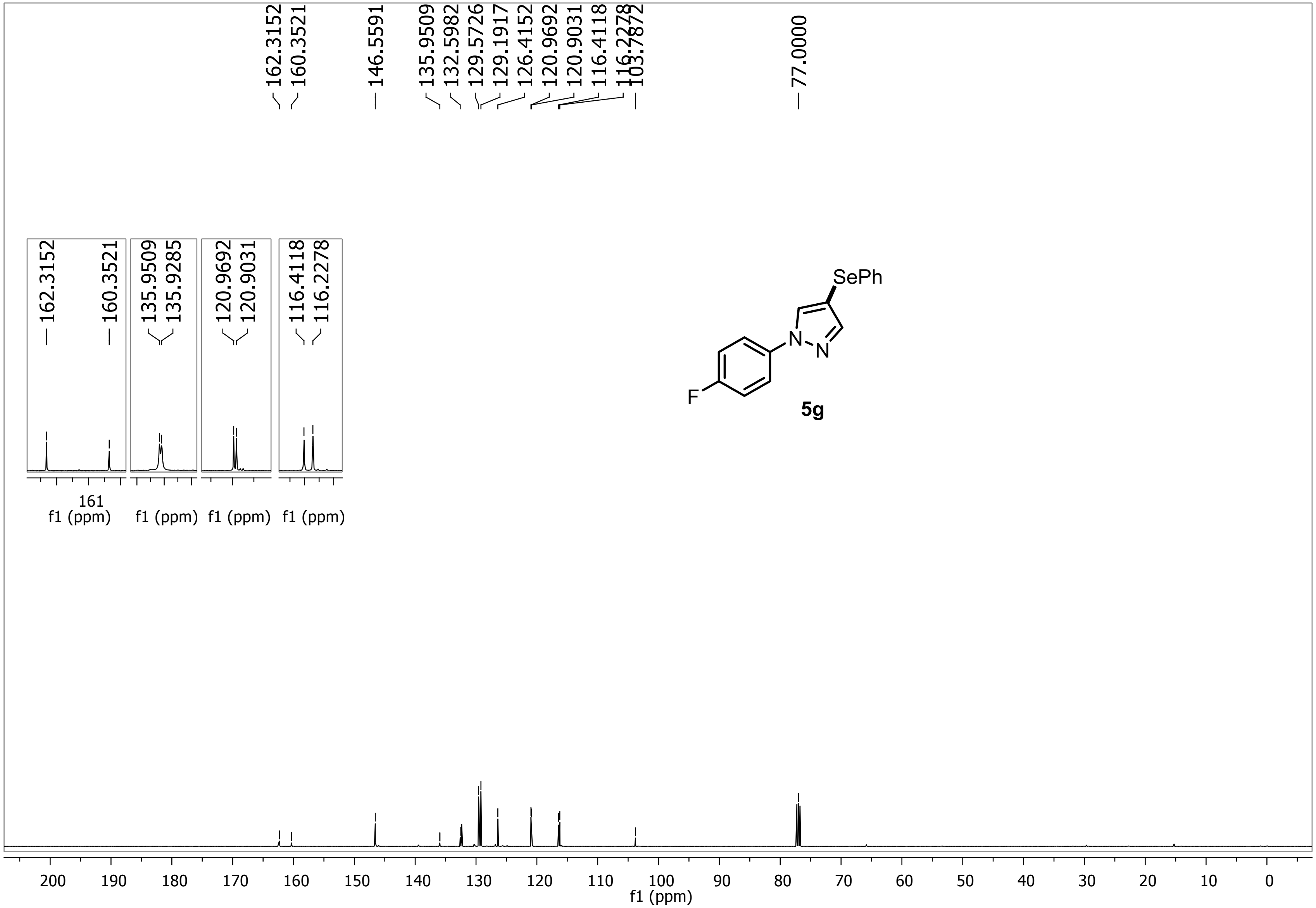

Figure S30: $^{13}C\{^{1}H\}$ NMR (125 MHz, $CDCl_3$) spectrum of compound **5g**.

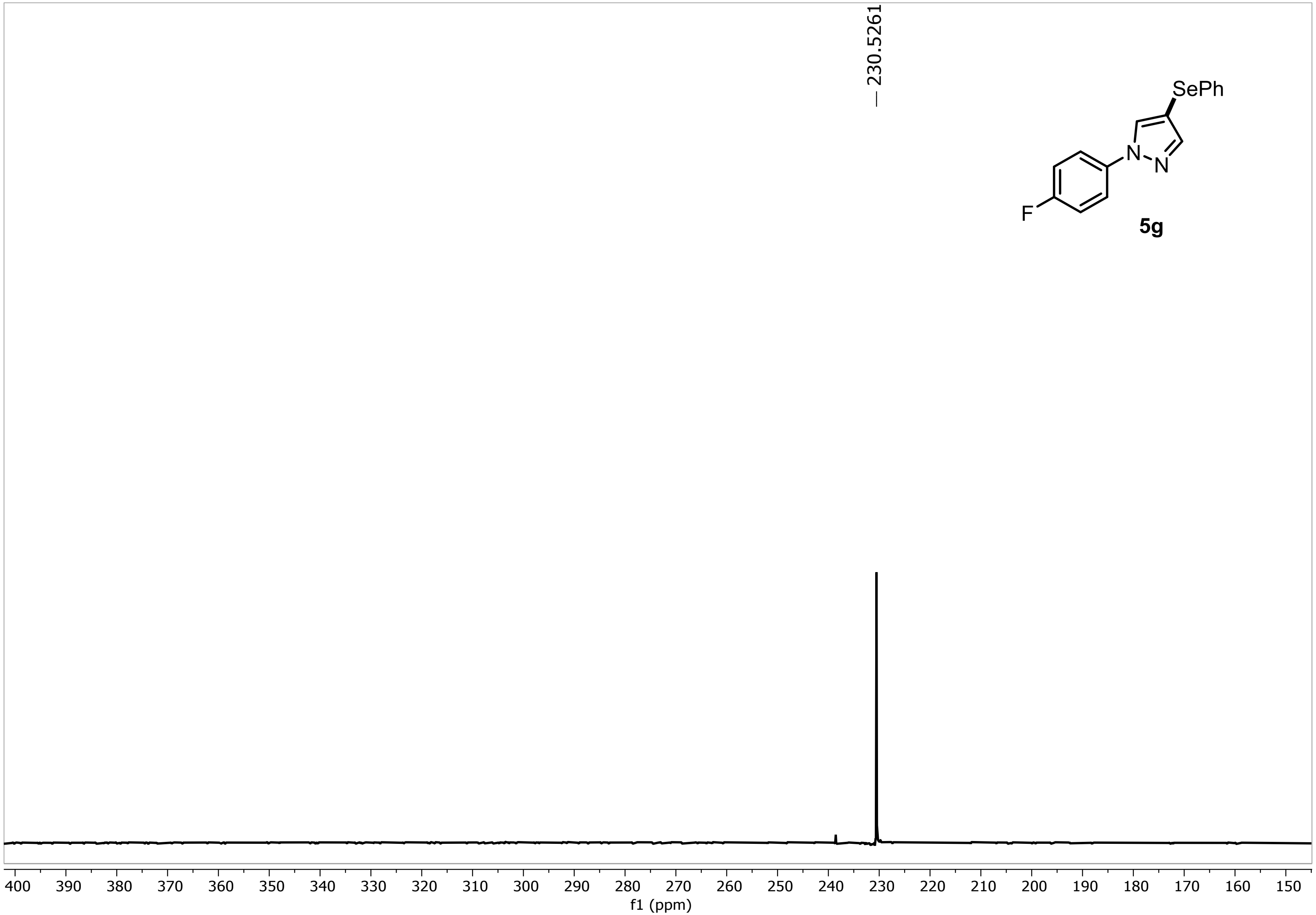

5g

Figure S31: ^{77}Se NMR (95 MHz, $CDCl_3$) spectrum of compound **5g**.

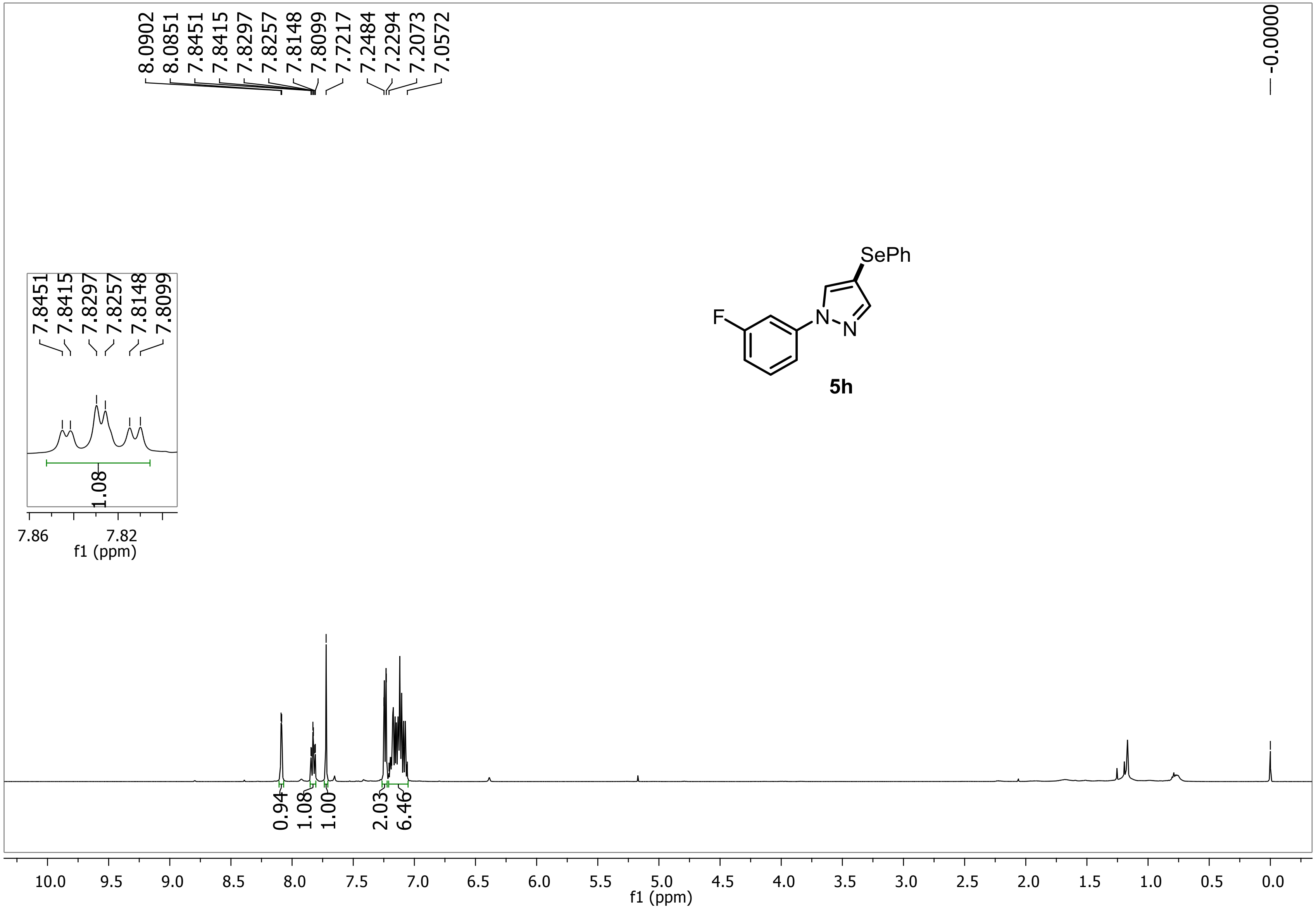

Figure S32: ^{1}H NMR (500 MHz, $CDCl_3$) spectrum of compound **5h**.

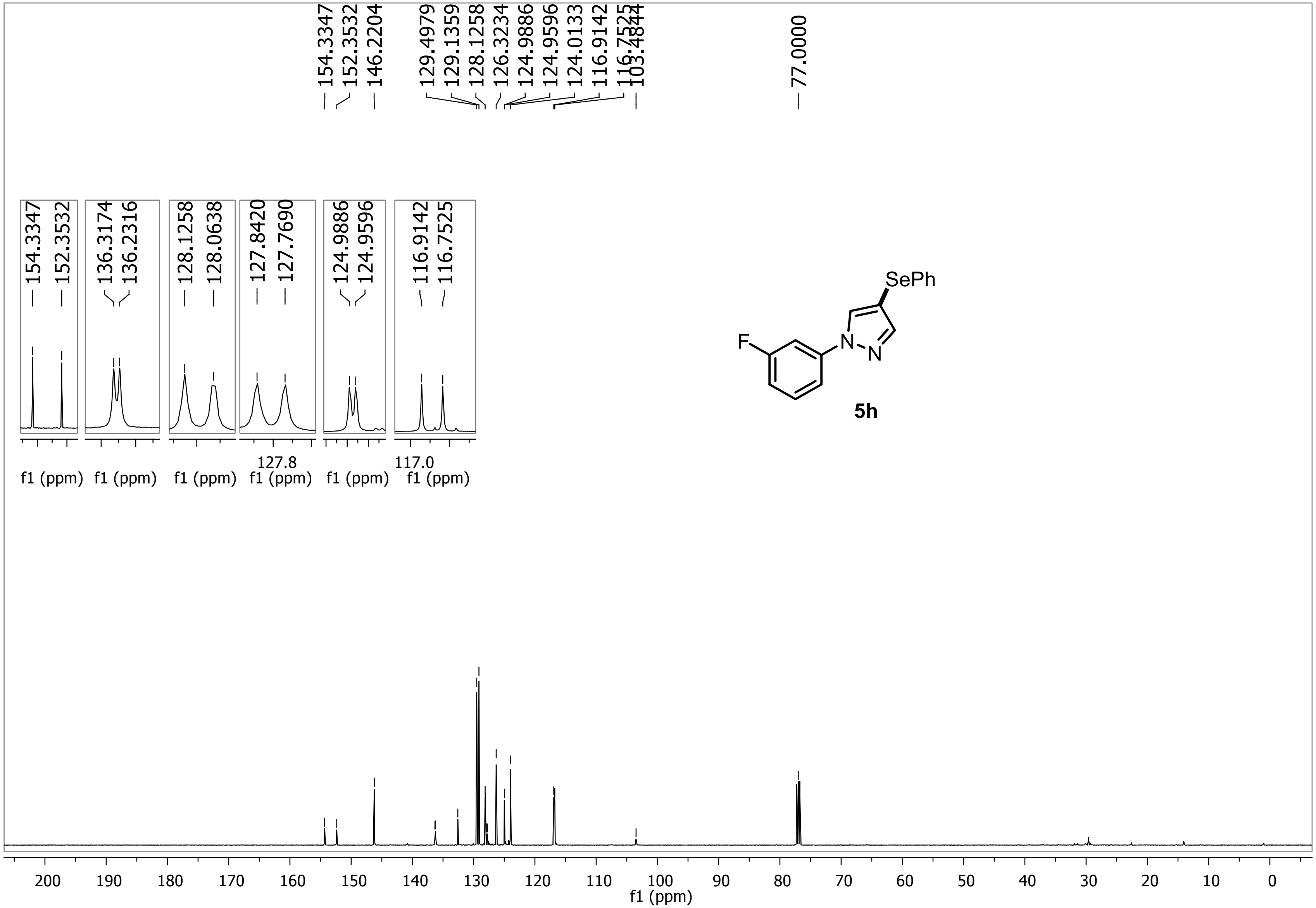

Figure S33: $^{13}C\{^{1}H\}$ NMR (125 MHz, $CDCl_3$) spectrum of compound **5h**.

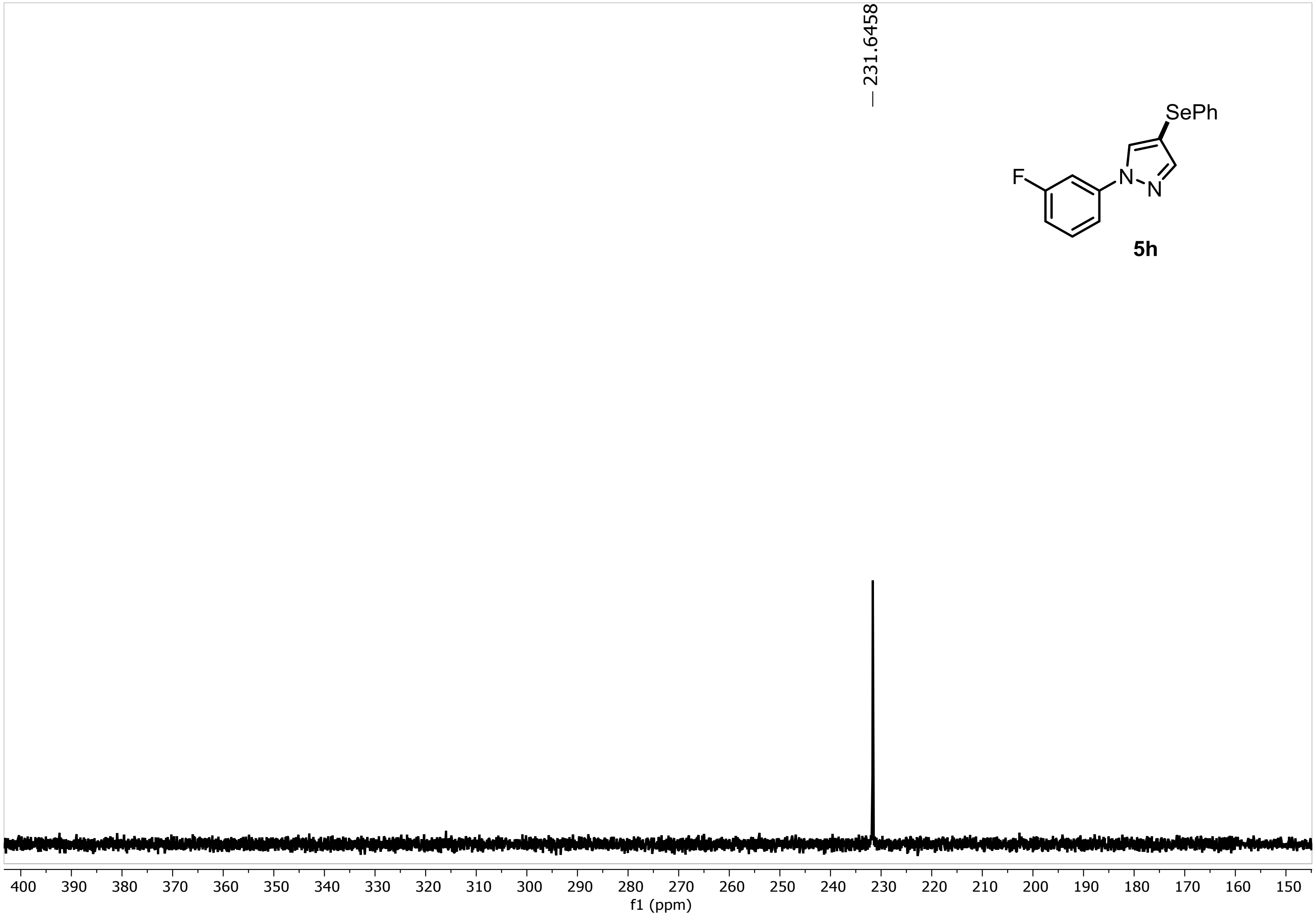

Figure S34: ^{77}Se NMR (95 MHz, $CDCl_3$) spectrum of compound **5h**.

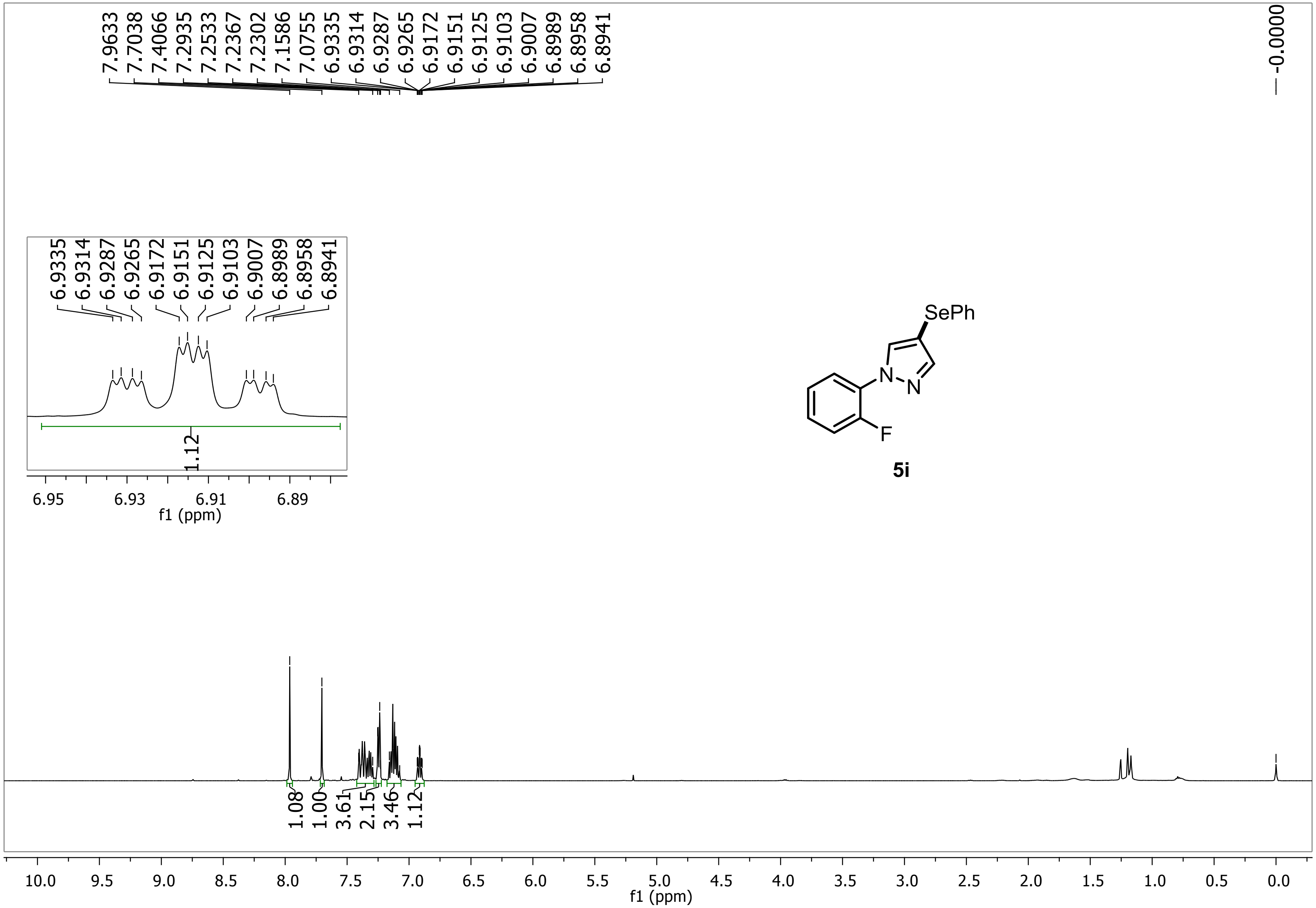

Figure S35: ^{1}H NMR (500 MHz, $CDCl_3$) spectrum of compound **5i**.

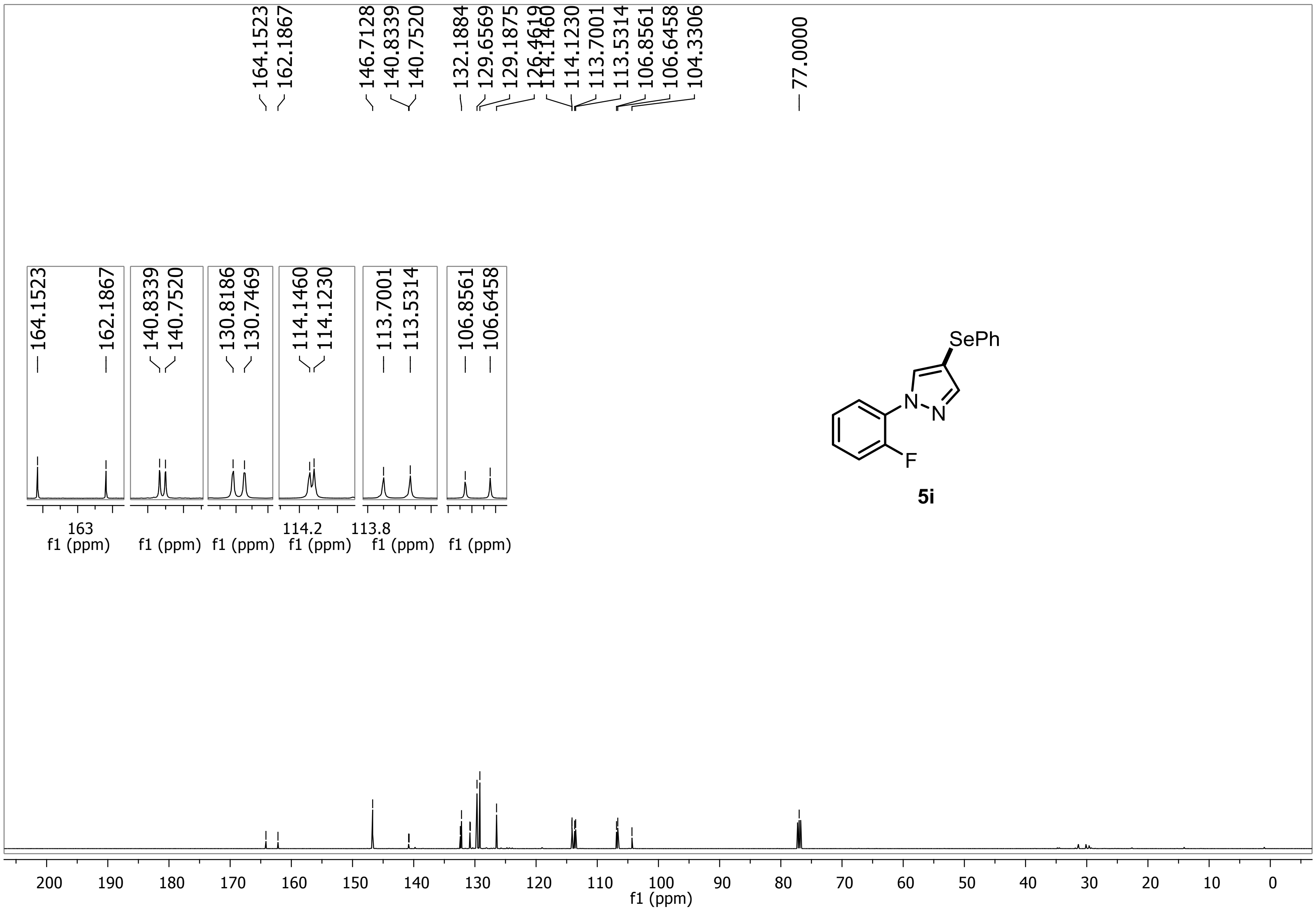

Figure S36: $^{13}C\{^{1}H\}$ NMR (125 MHz, $CDCl_3$) spectrum of compound **5i**.

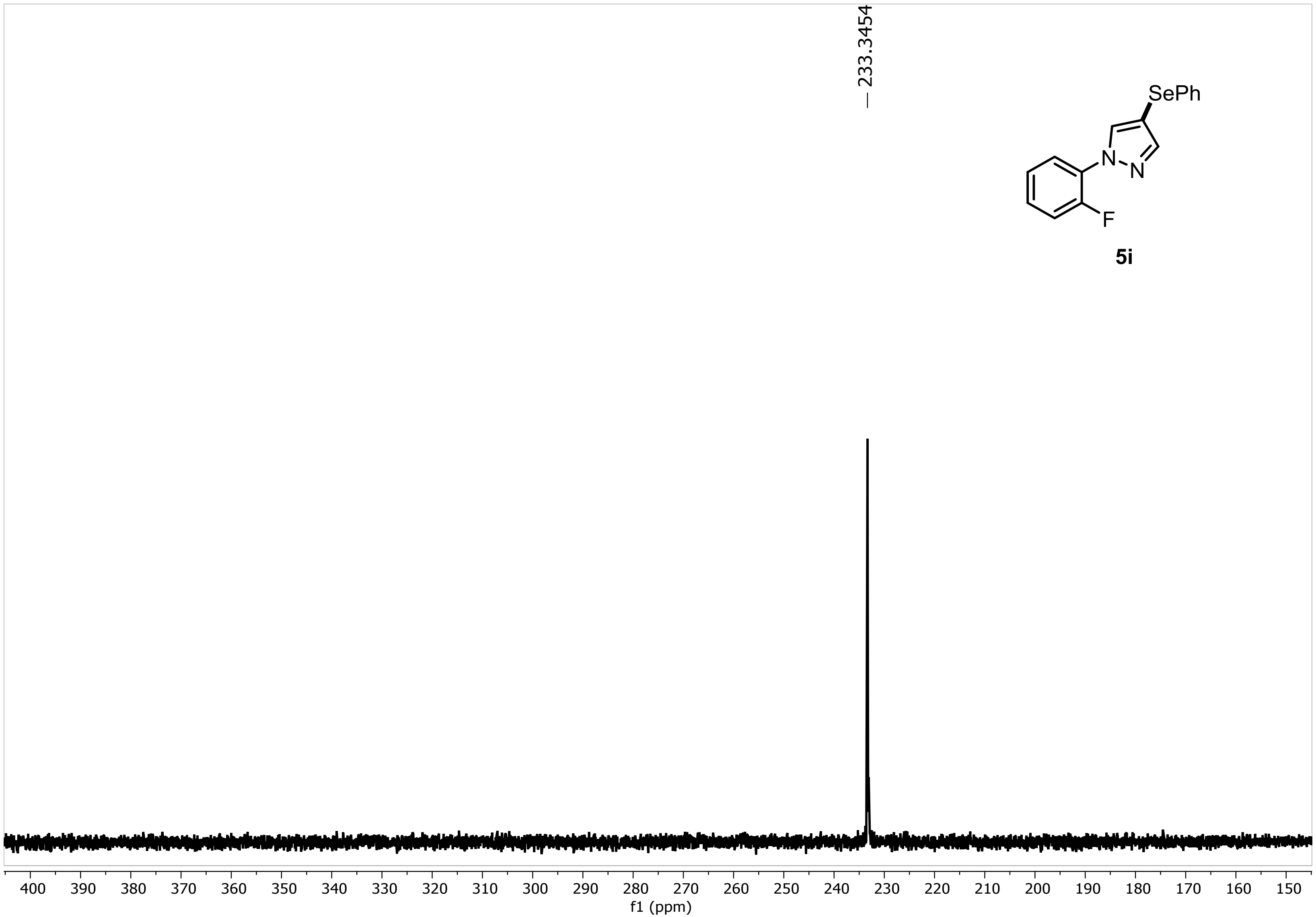

Figure S37: ^{77}Se NMR (95 MHz, $CDCl_3$) spectrum of compound **5i**.

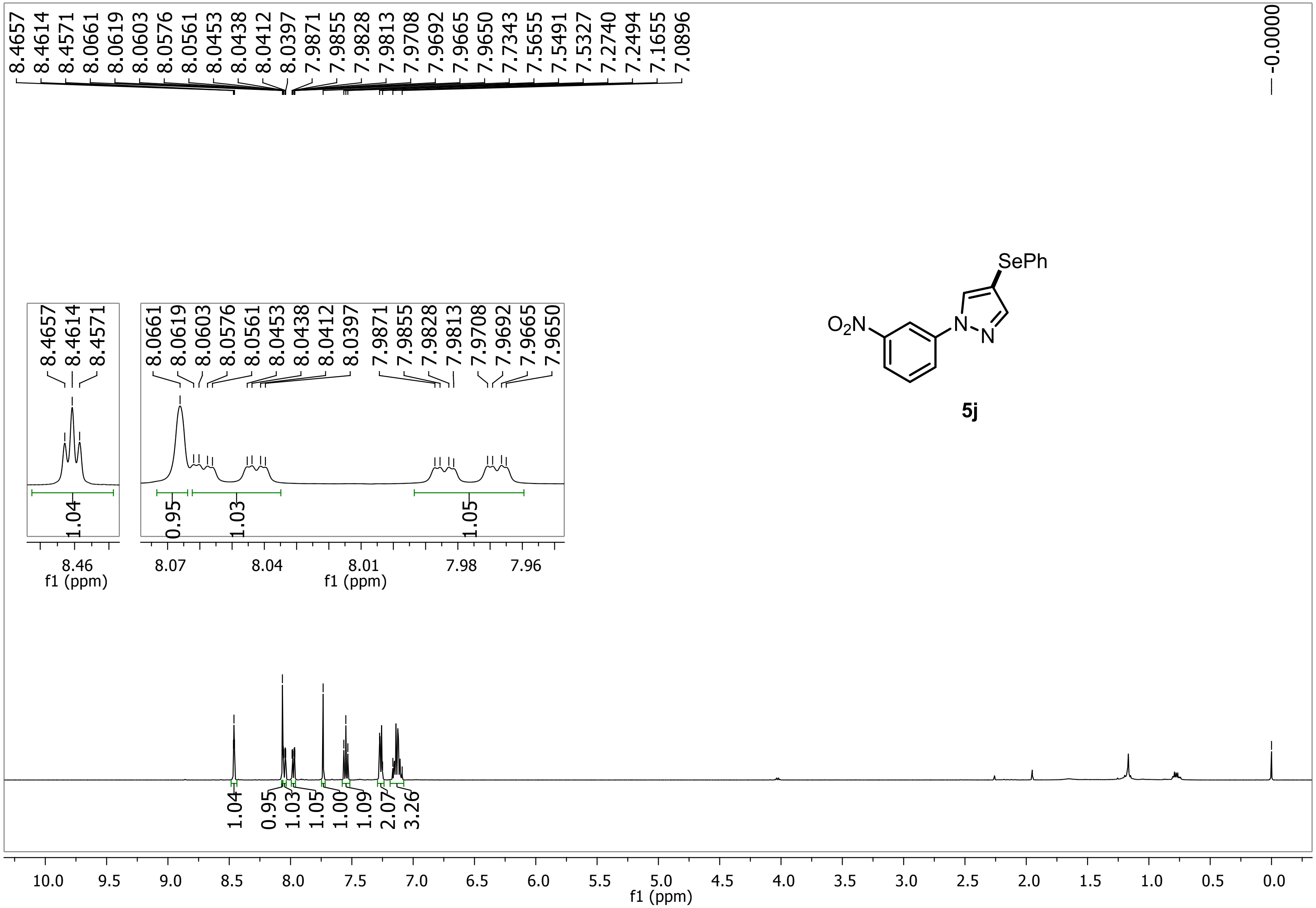

Figure S38: ^1H NMR (500 MHz, $CDCl_3$) spectrum of compound **5j**.

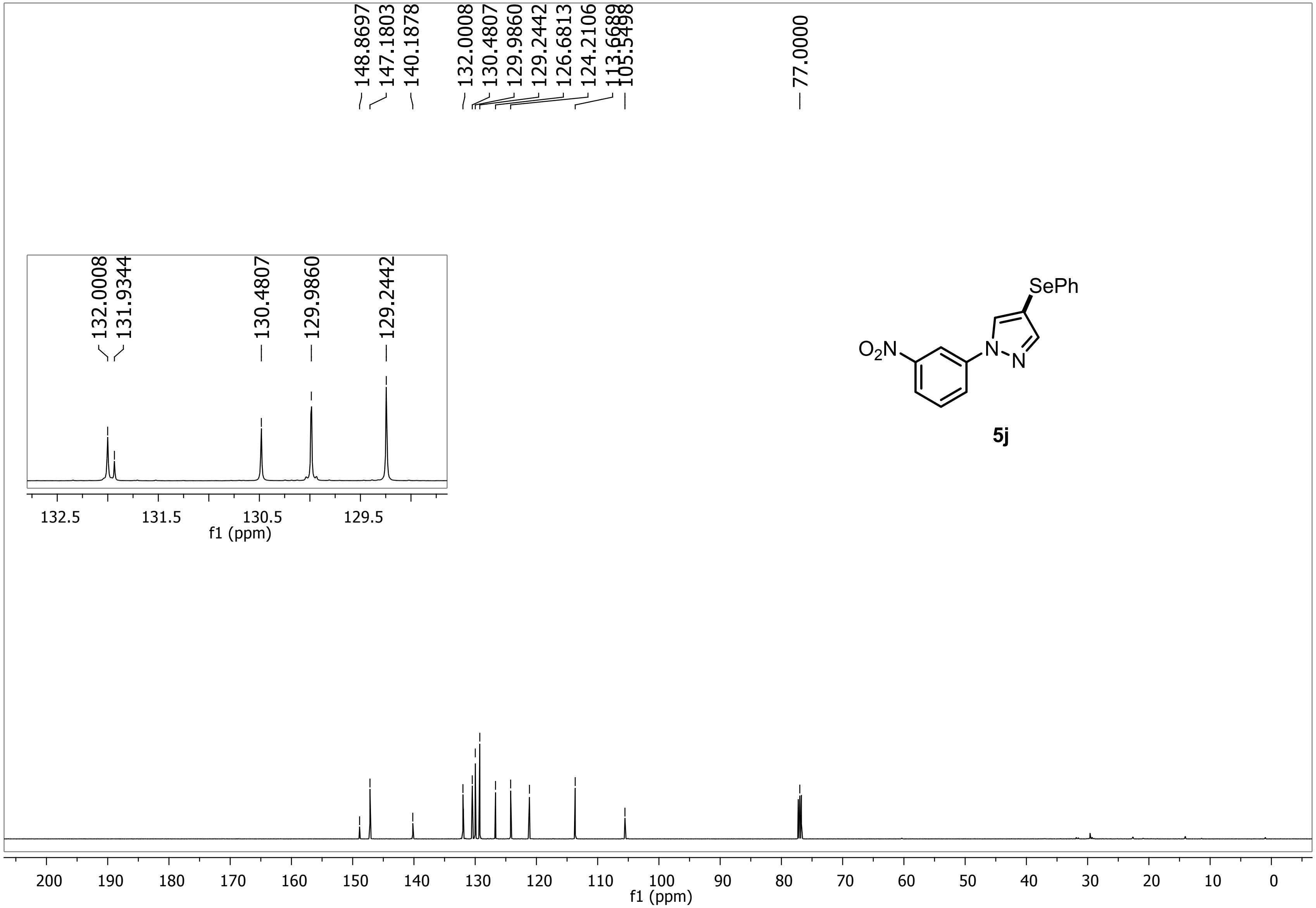

Figure S39: $^{13}C\{^1H\}$ NMR (125 MHz, $CDCl_3$) spectrum of compound **5j**.

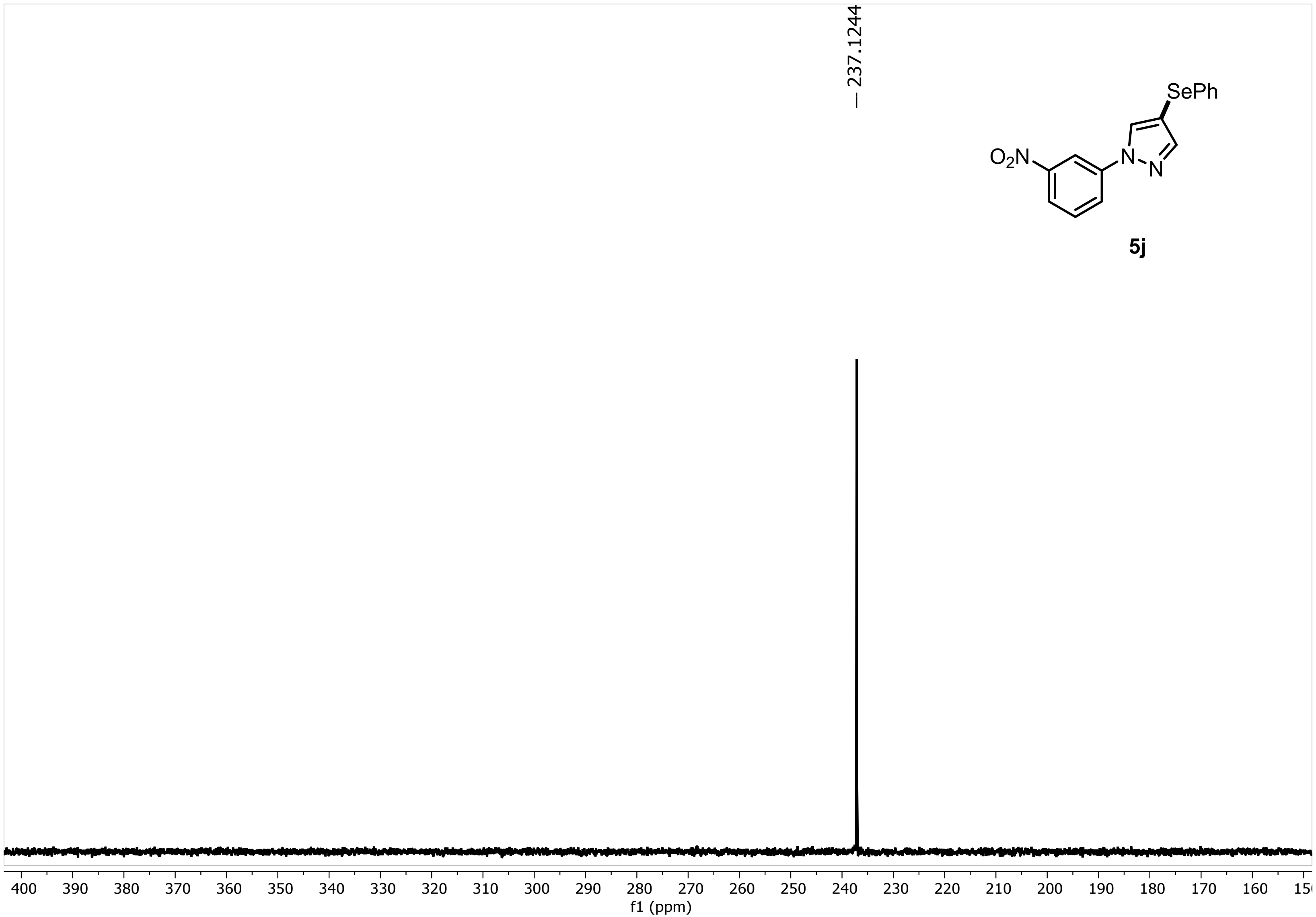

Figure S40: ^{77}Se NMR (95 MHz, $CDCl_3$) spectrum of compound **5j**.

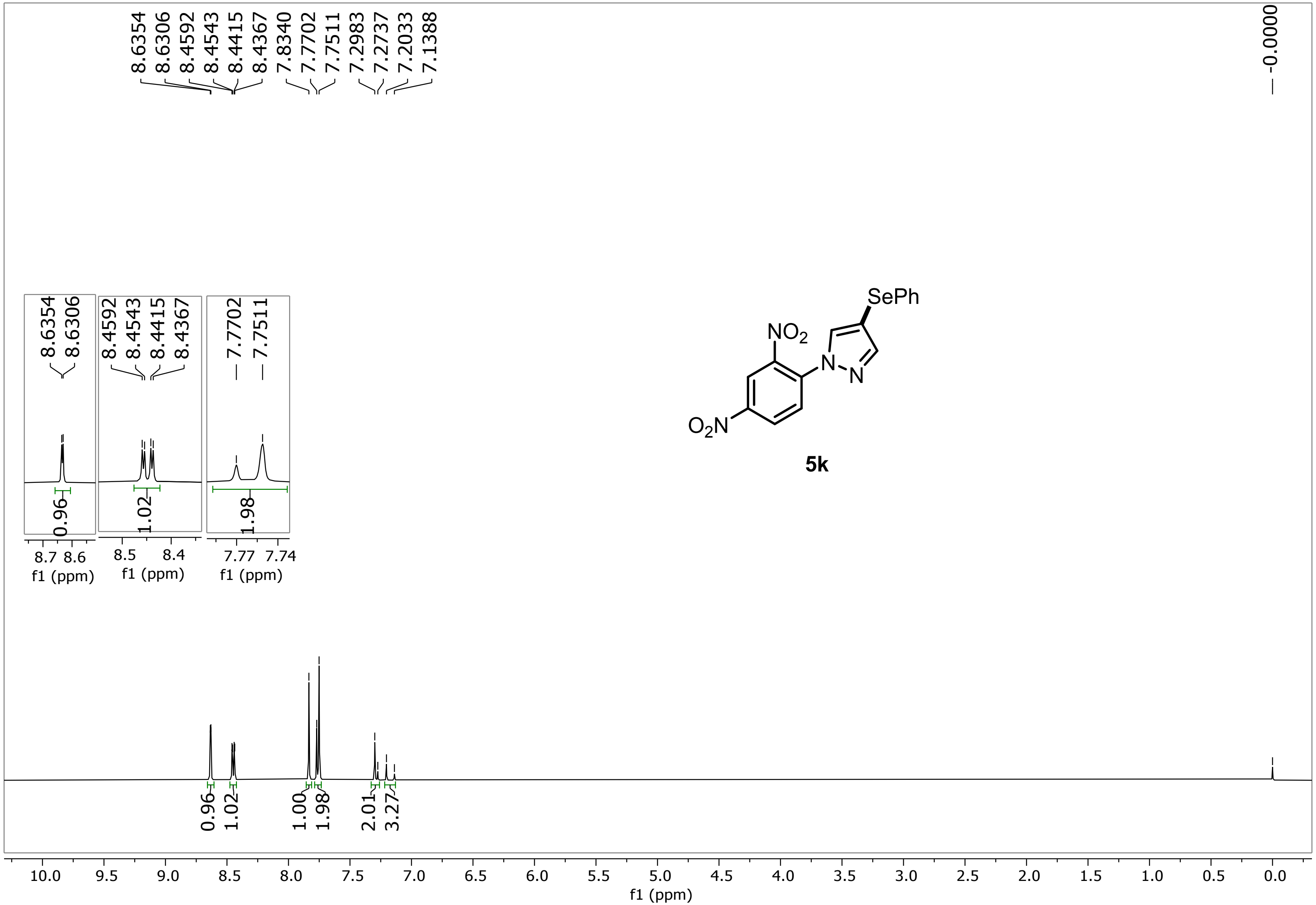

Figure S41: ^{1}H NMR (500 MHz, $CDCl_3$) spectrum of compound **5k**.

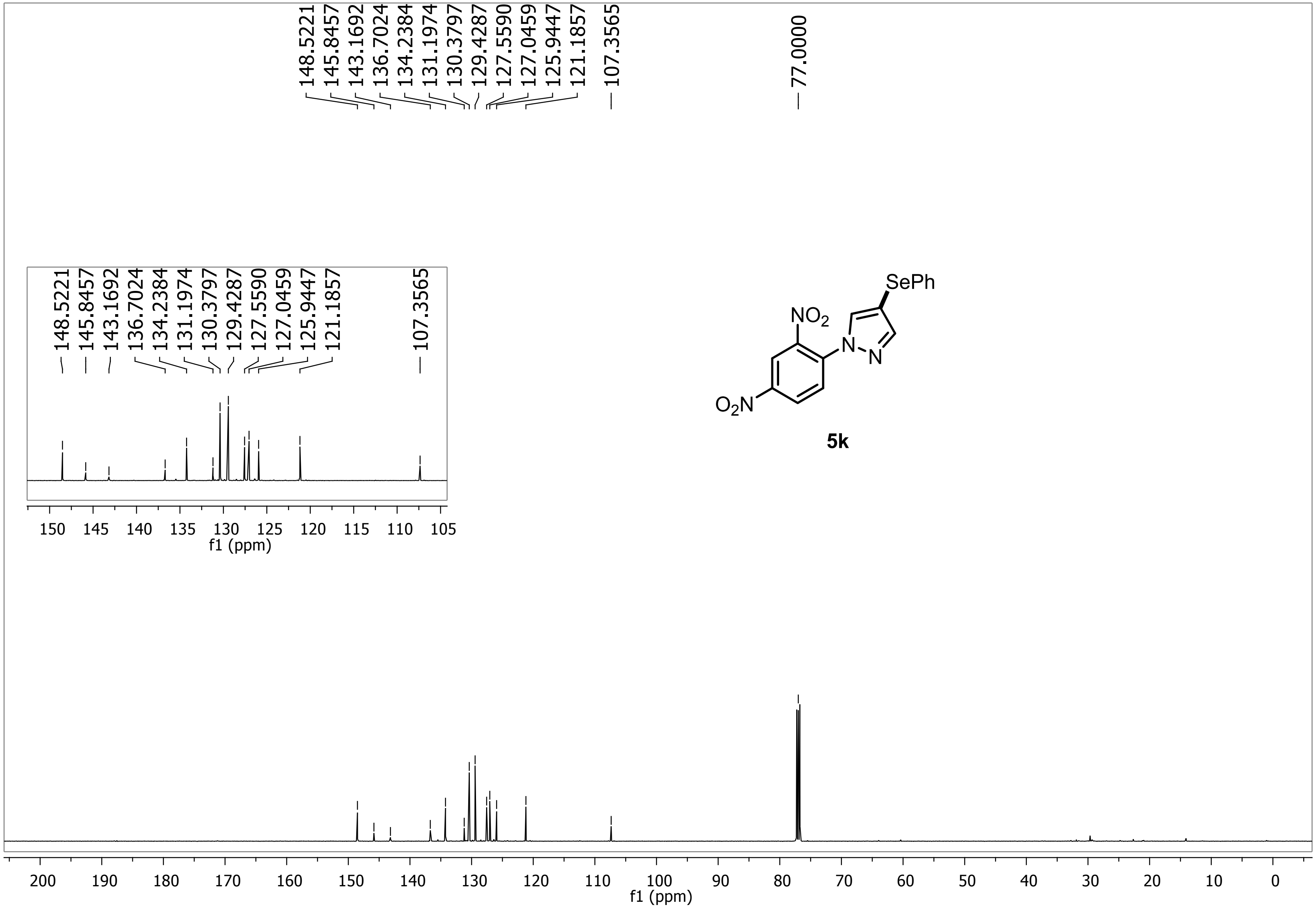

Figure S42: $^{13}C\{^1H\}$ NMR (125 MHz, $CDCl_3$) spectrum of compound **5k**.

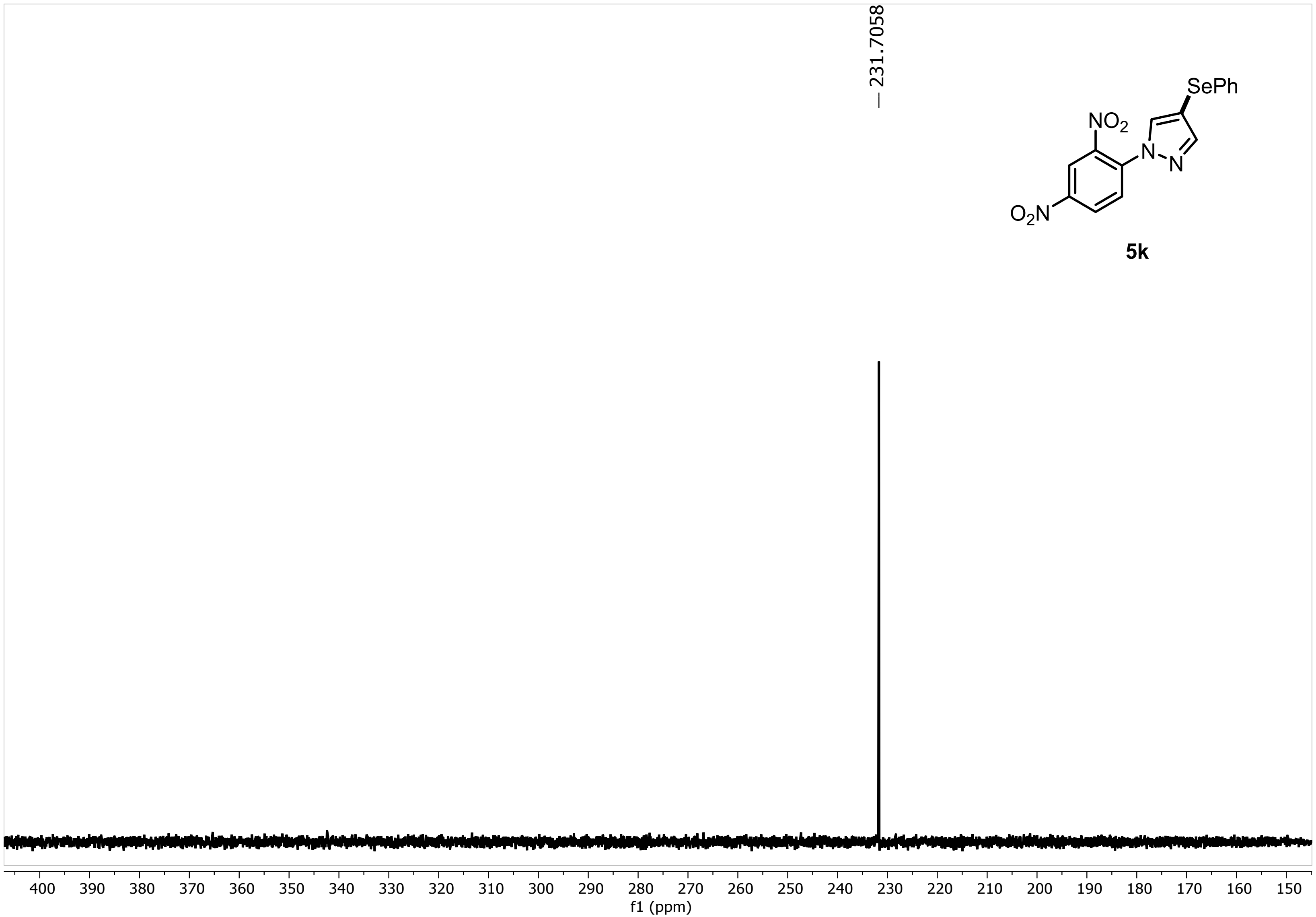

Figure S43: ^{77}Se NMR (95 MHz, $CDCl_3$) spectrum of compound **5k**.

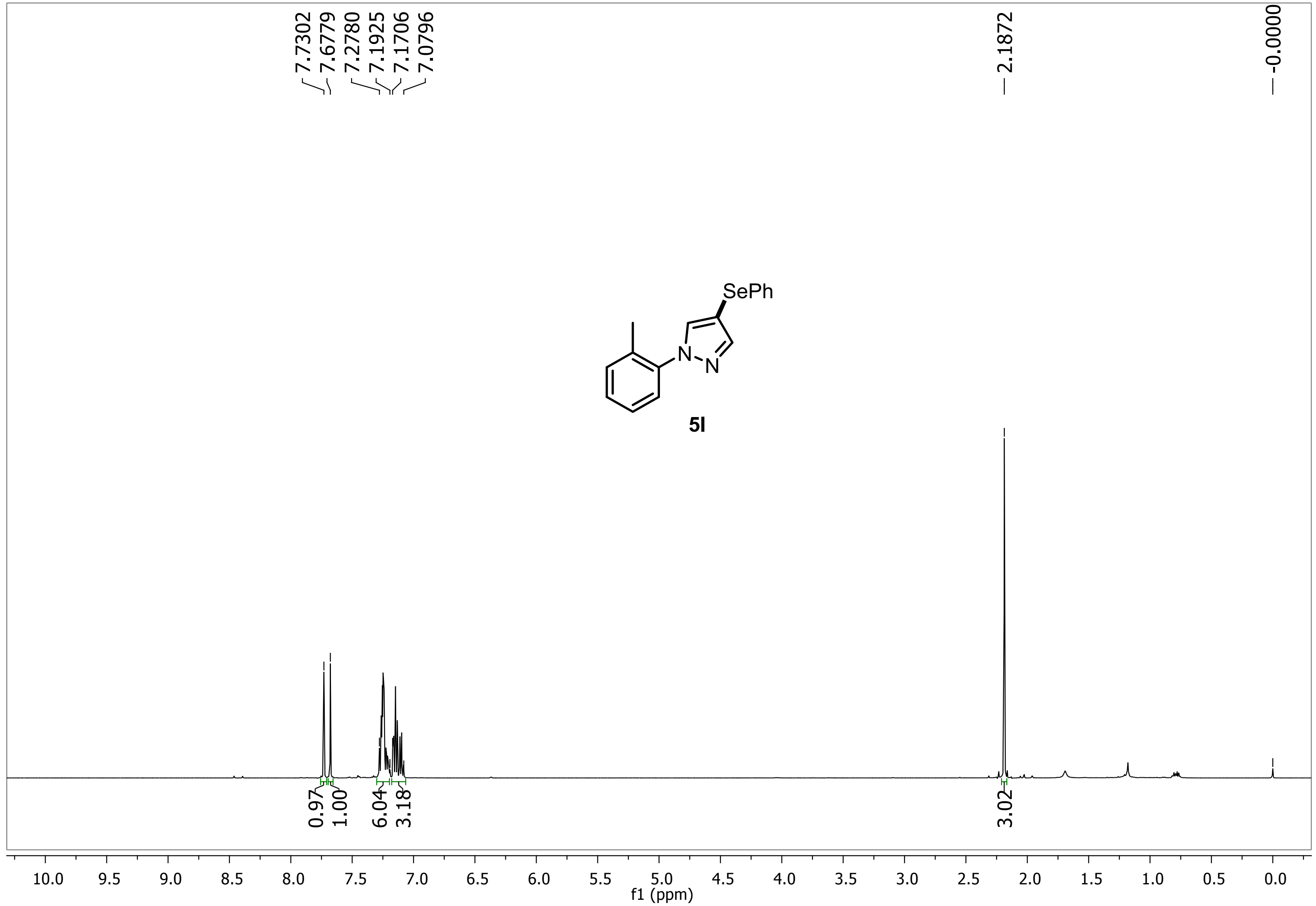

Figure S44: ^{1}H NMR (500 MHz, $CDCl_3$) spectrum of compound **5l**.

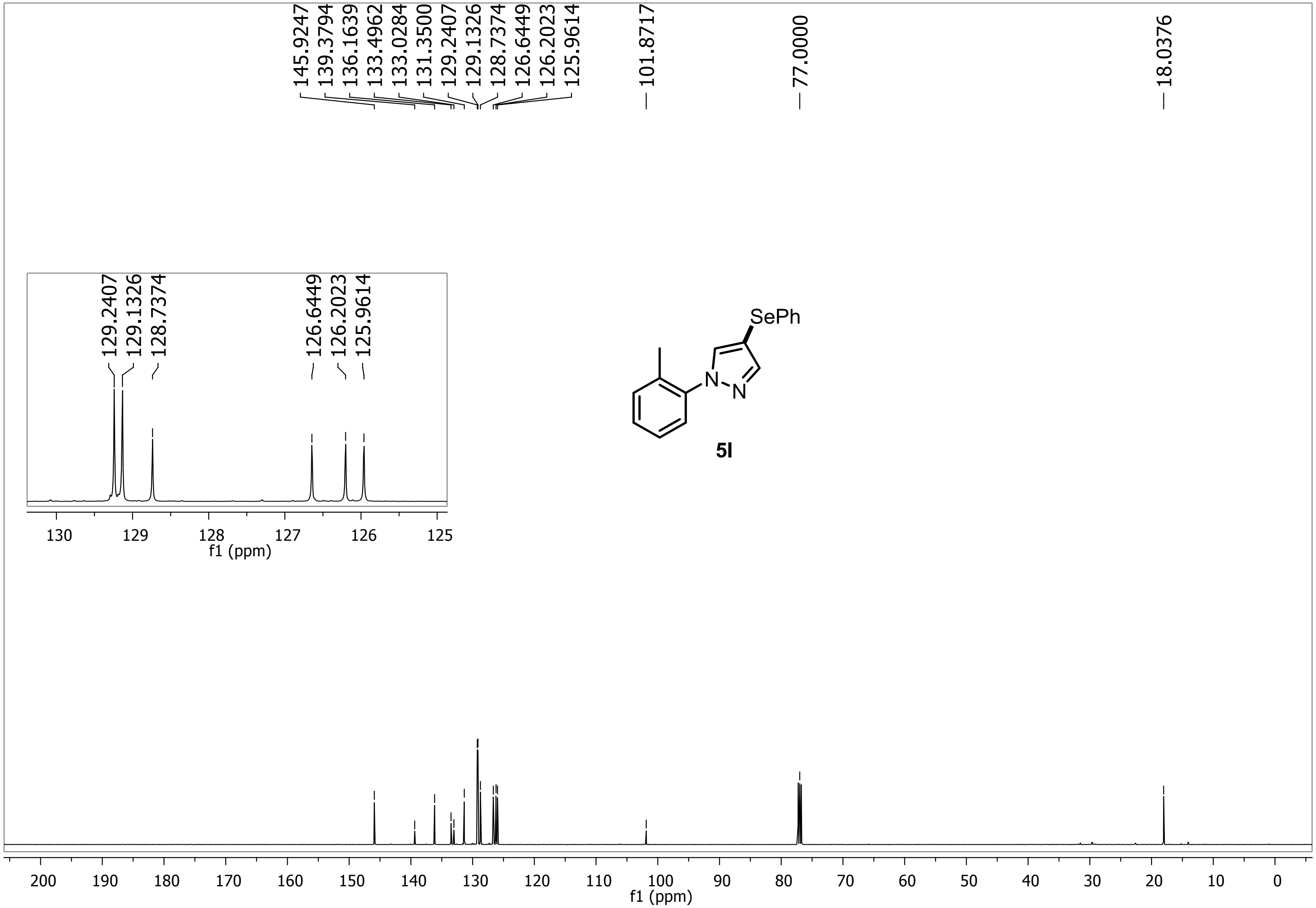

Figure S45: $^{13}C\{^{1}H\}$ NMR (125 MHz, $CDCl_3$) spectrum of compound **5l**.

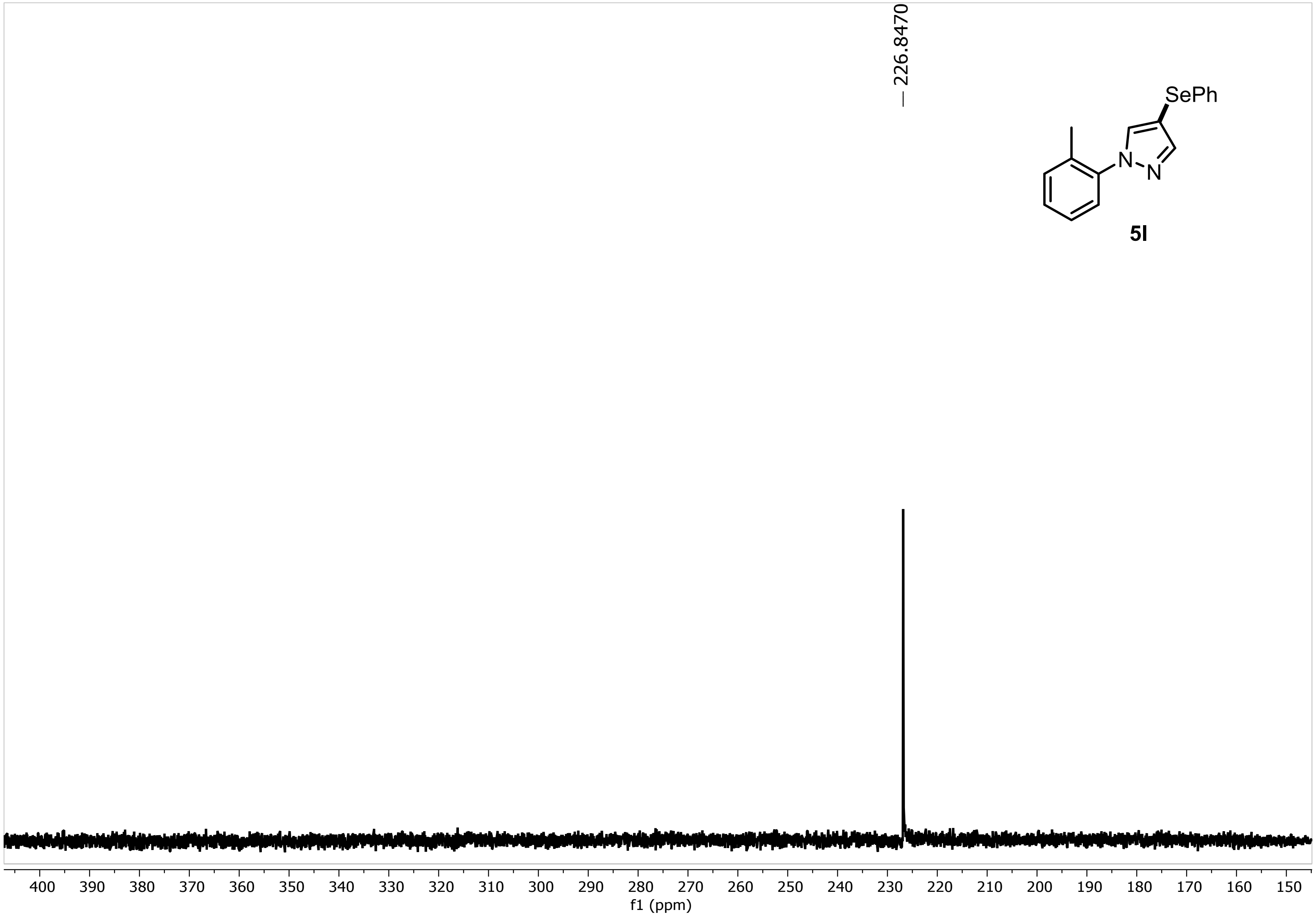

Figure S46: ^{77}Se NMR (95 MHz, $CDCl_3$) spectrum of compound **5l**.

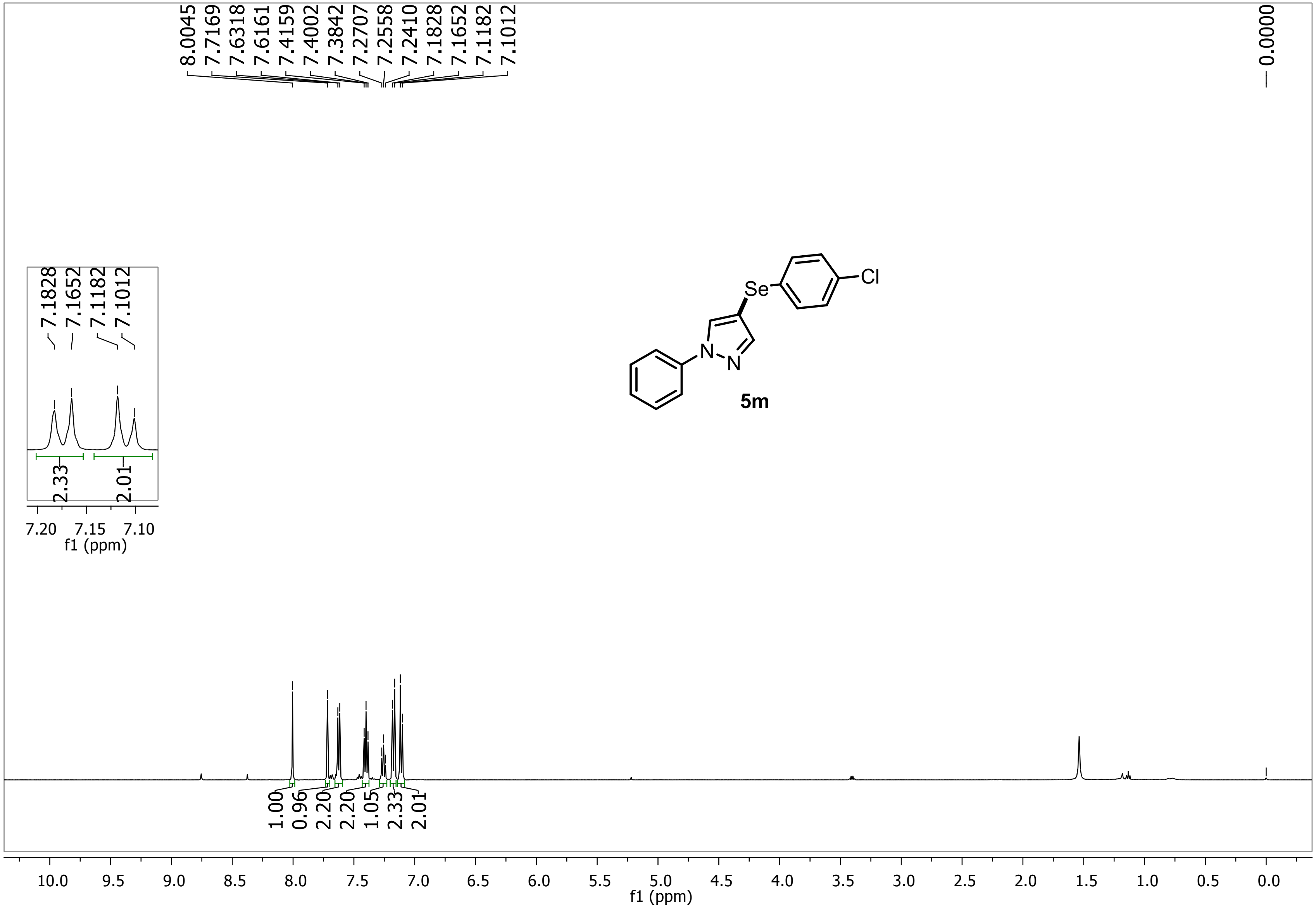

Figure S47: ^{1}H NMR (500 MHz, $CDCl_3$) spectrum of compound **5m**.

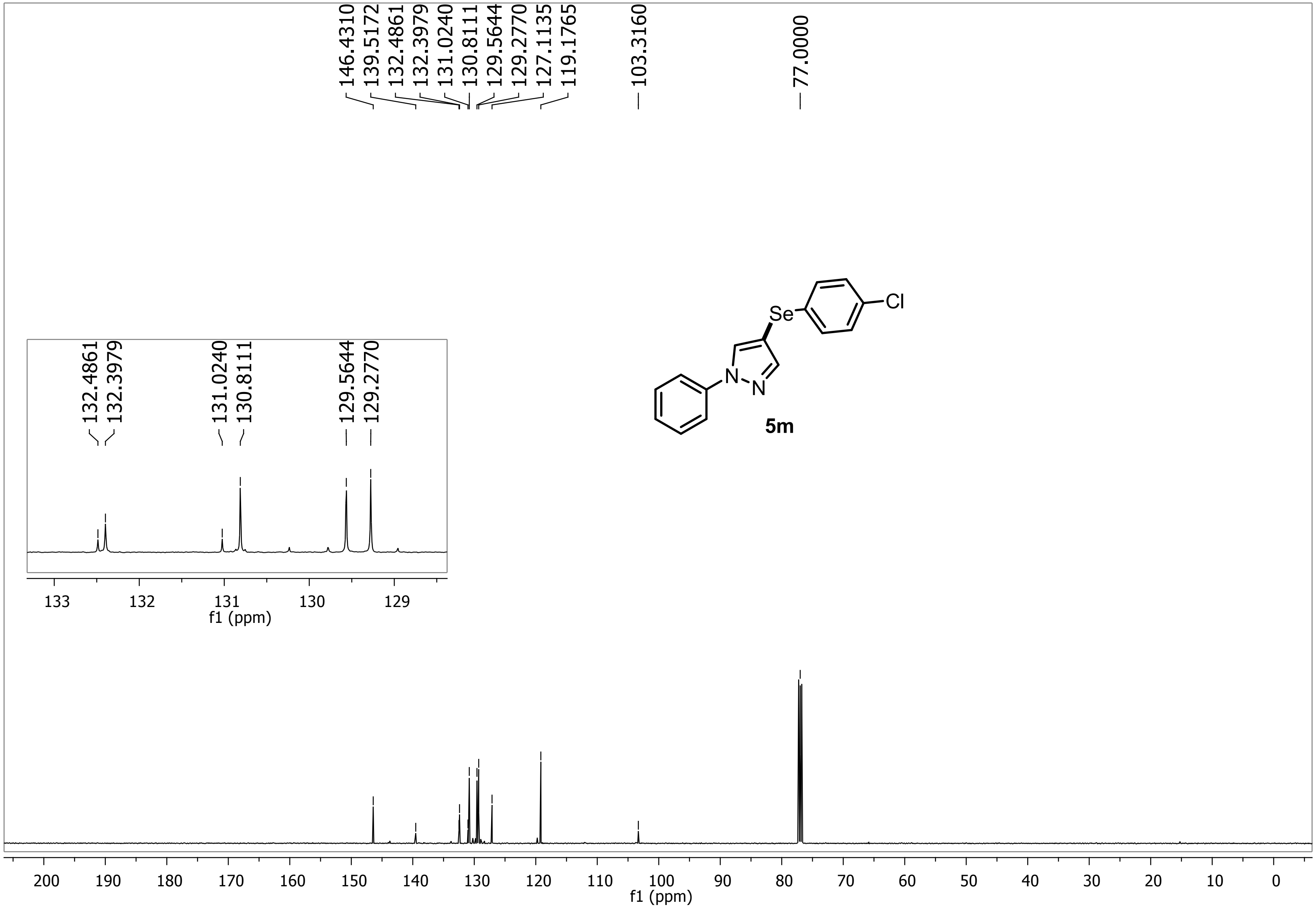

Figure S48: $^{13}C\{^{1}H\}$ NMR (125 MHz, $CDCl_3$) spectrum of compound **5m**.

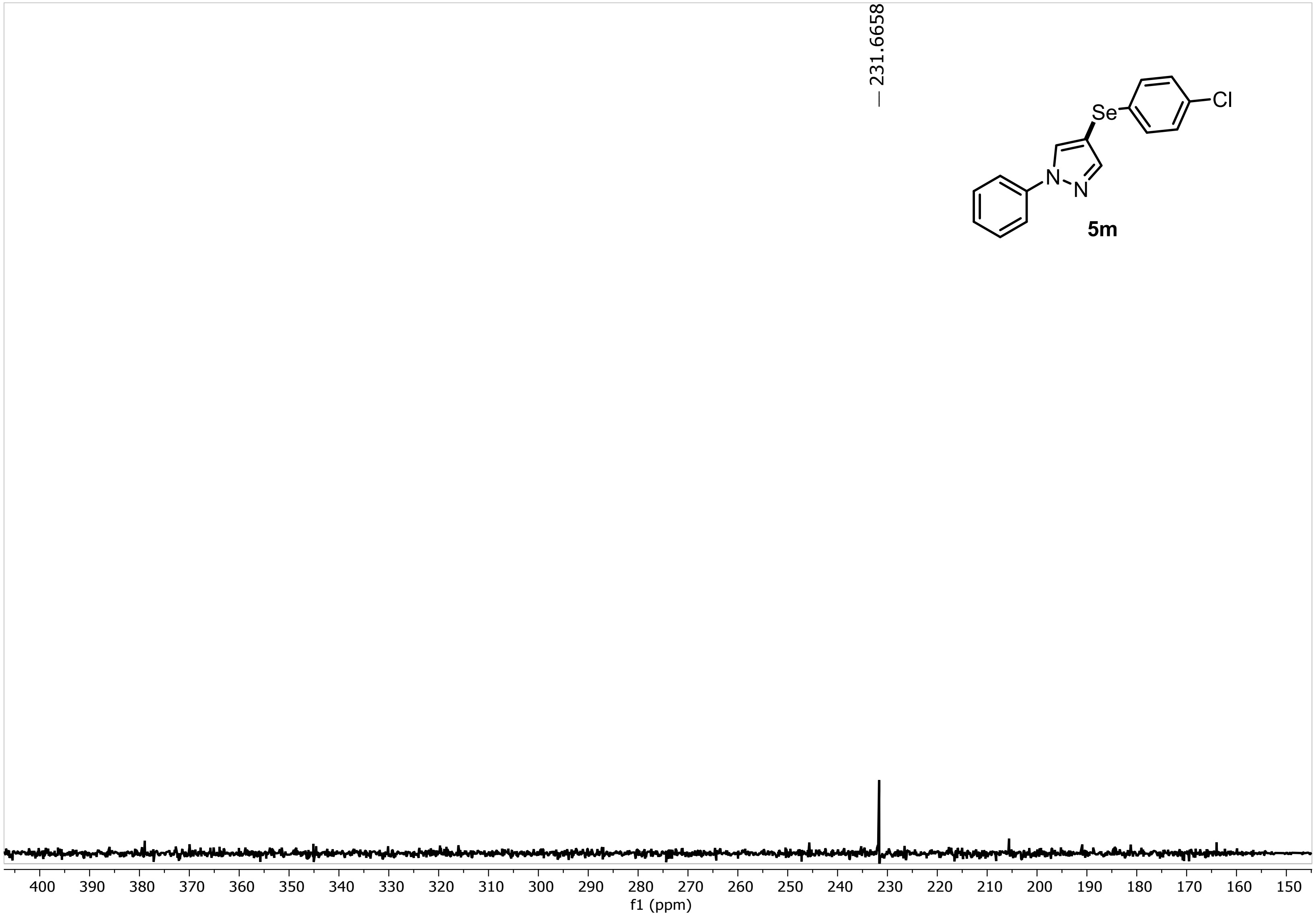

Figure S49: ^{77}Se NMR (95 MHz, $CDCl_3$) spectrum of compound **5m**.

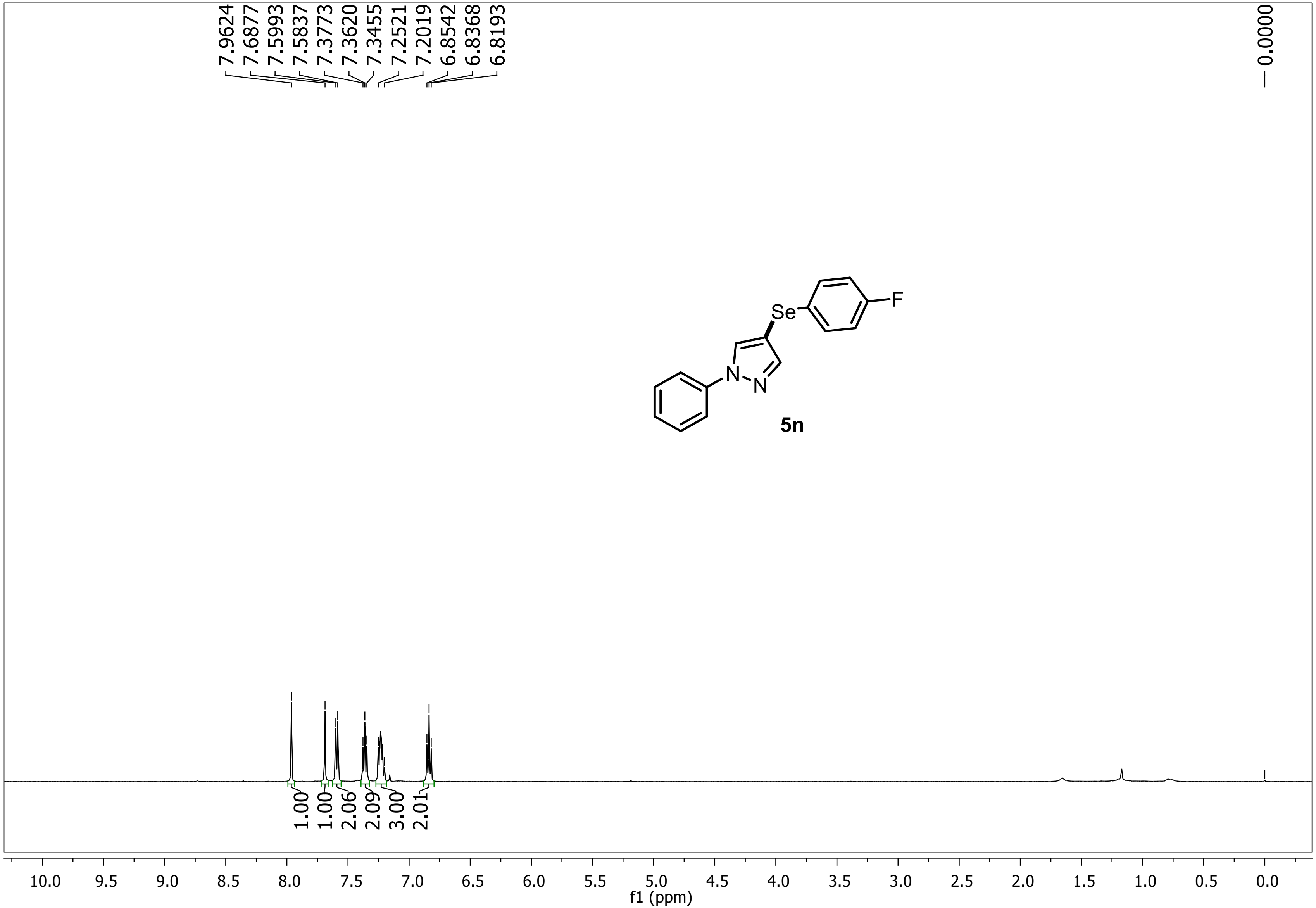

Figure S50: ^{1}H NMR (500 MHz, $CDCl_3$) spectrum of compound **5n**.

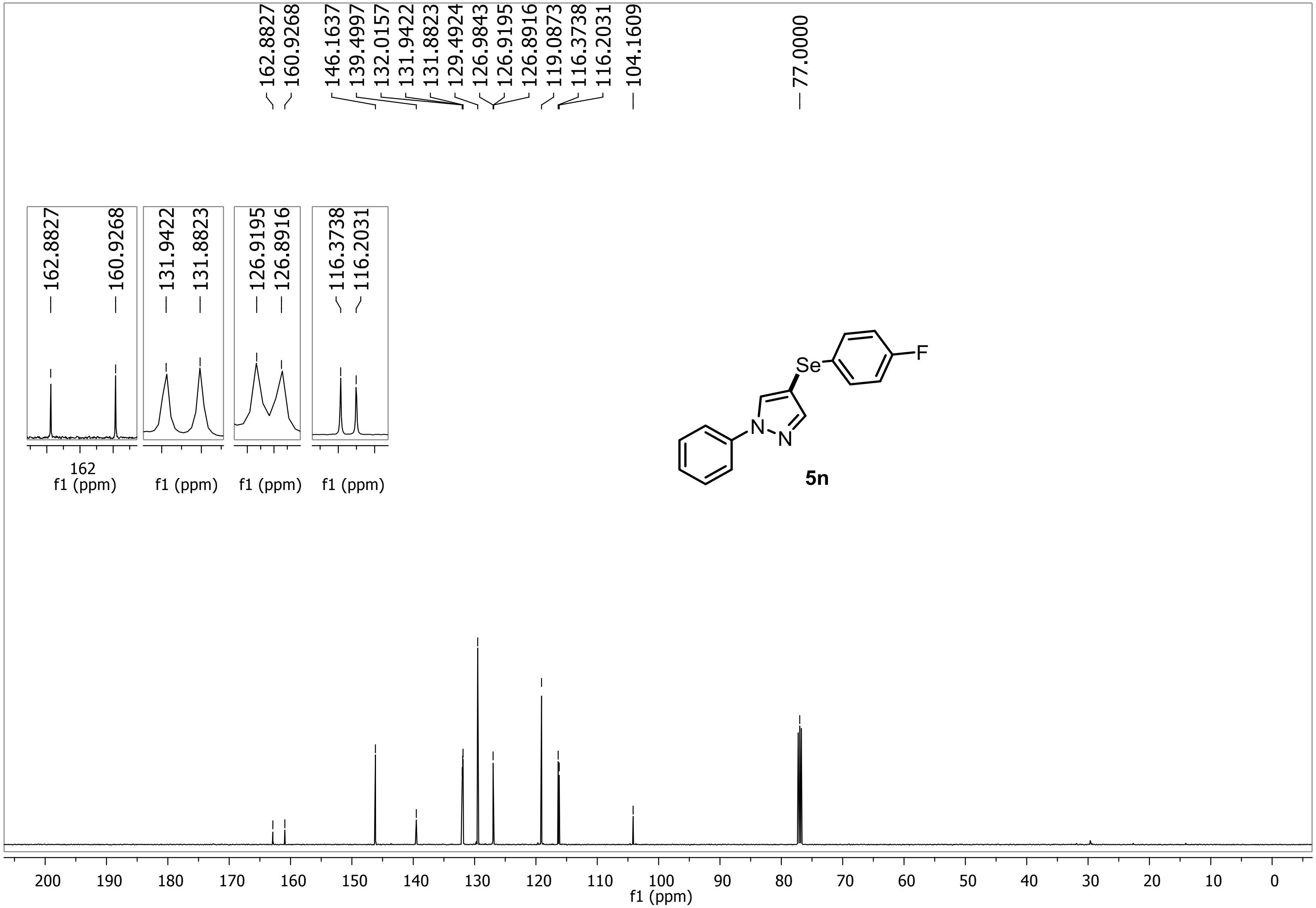

Figure S51: $^{13}C\{^{1}H\}$ NMR (125 MHz, $CDCl_3$) spectrum of compound **5n**.

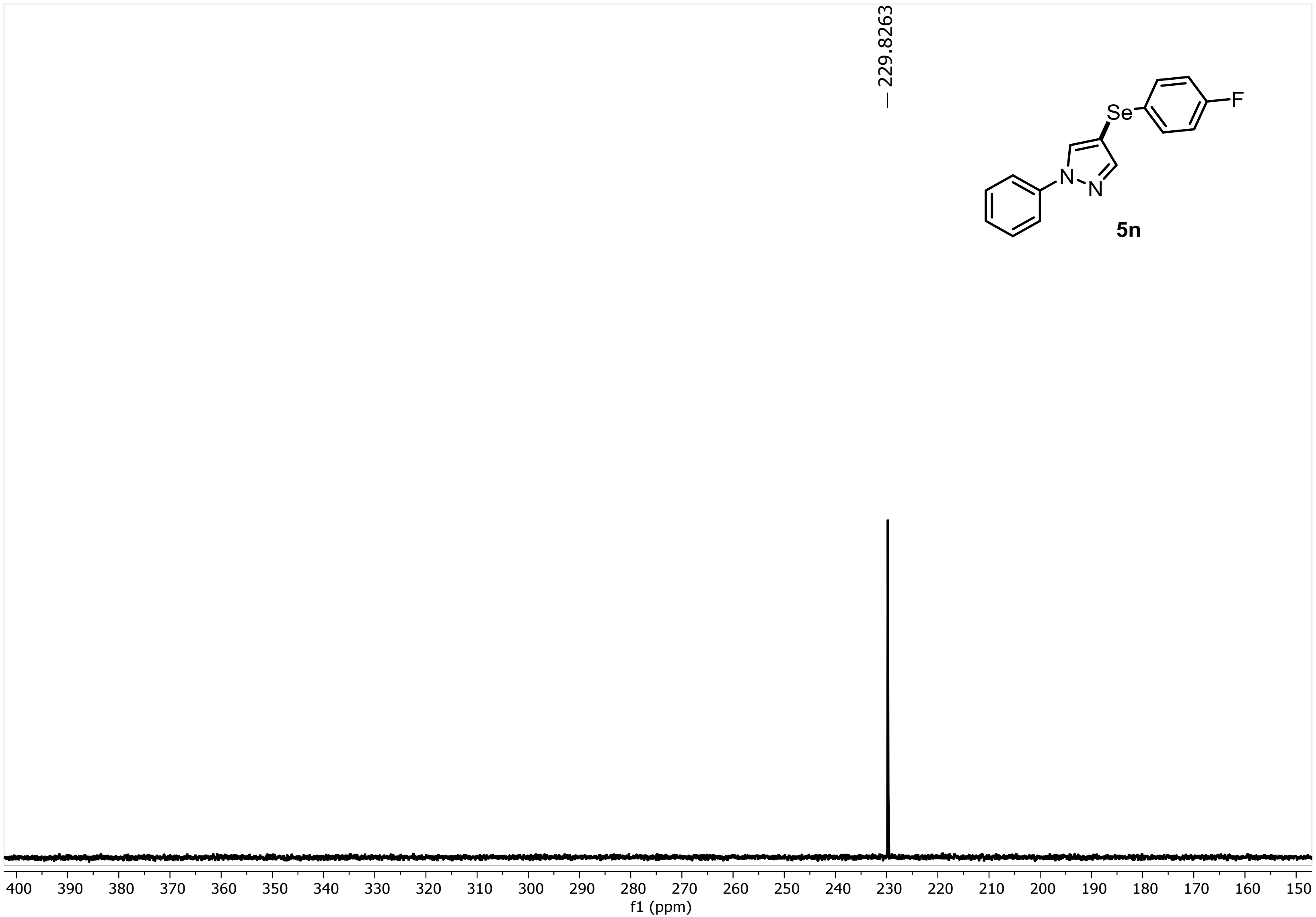

Figure S52: ^{77}Se NMR (95 MHz, $CDCl_3$) spectrum of compound **5n**.

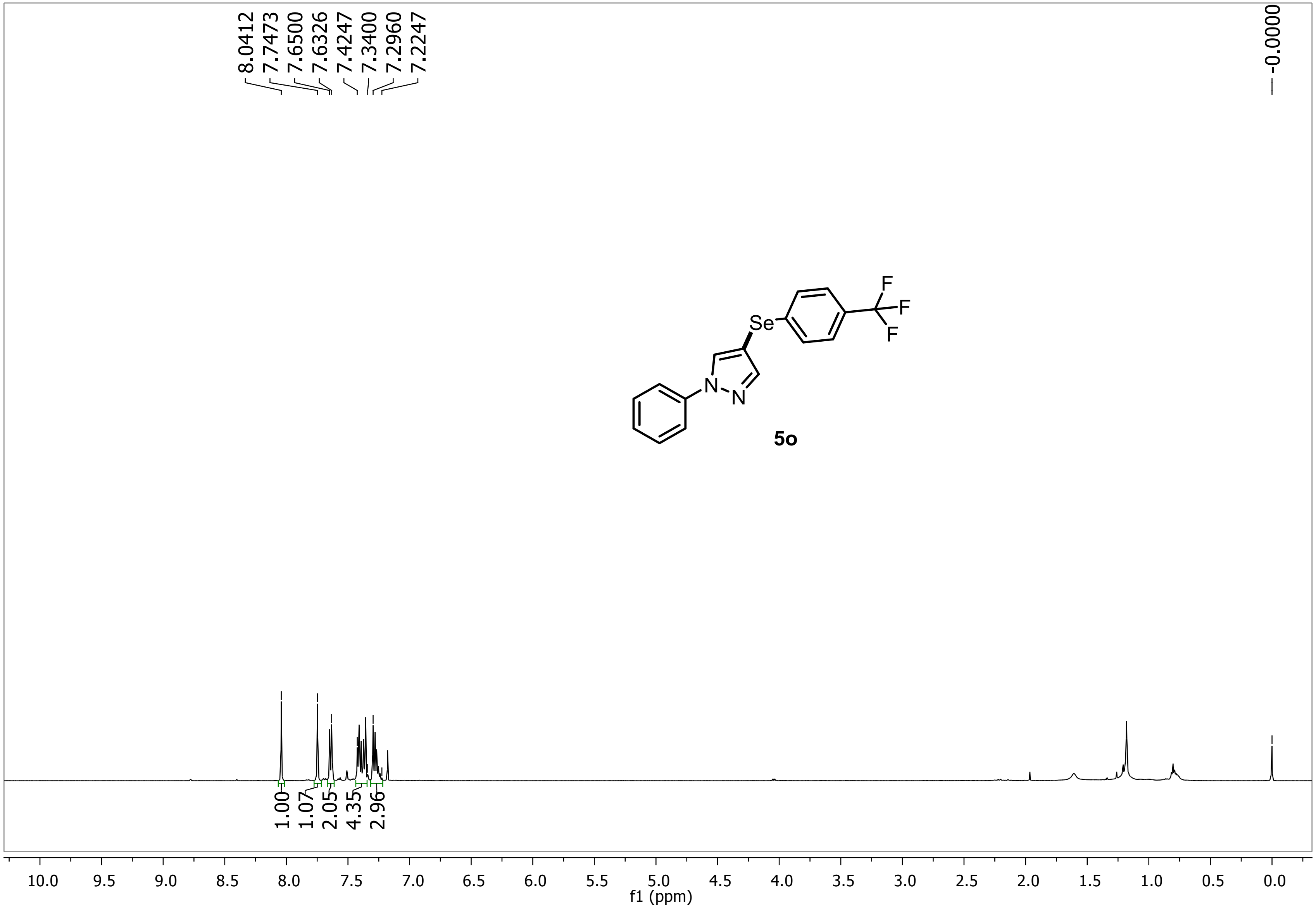

Figure S53: ^{1}H NMR (500 MHz, $CDCl_3$) spectrum of compound **5o**.

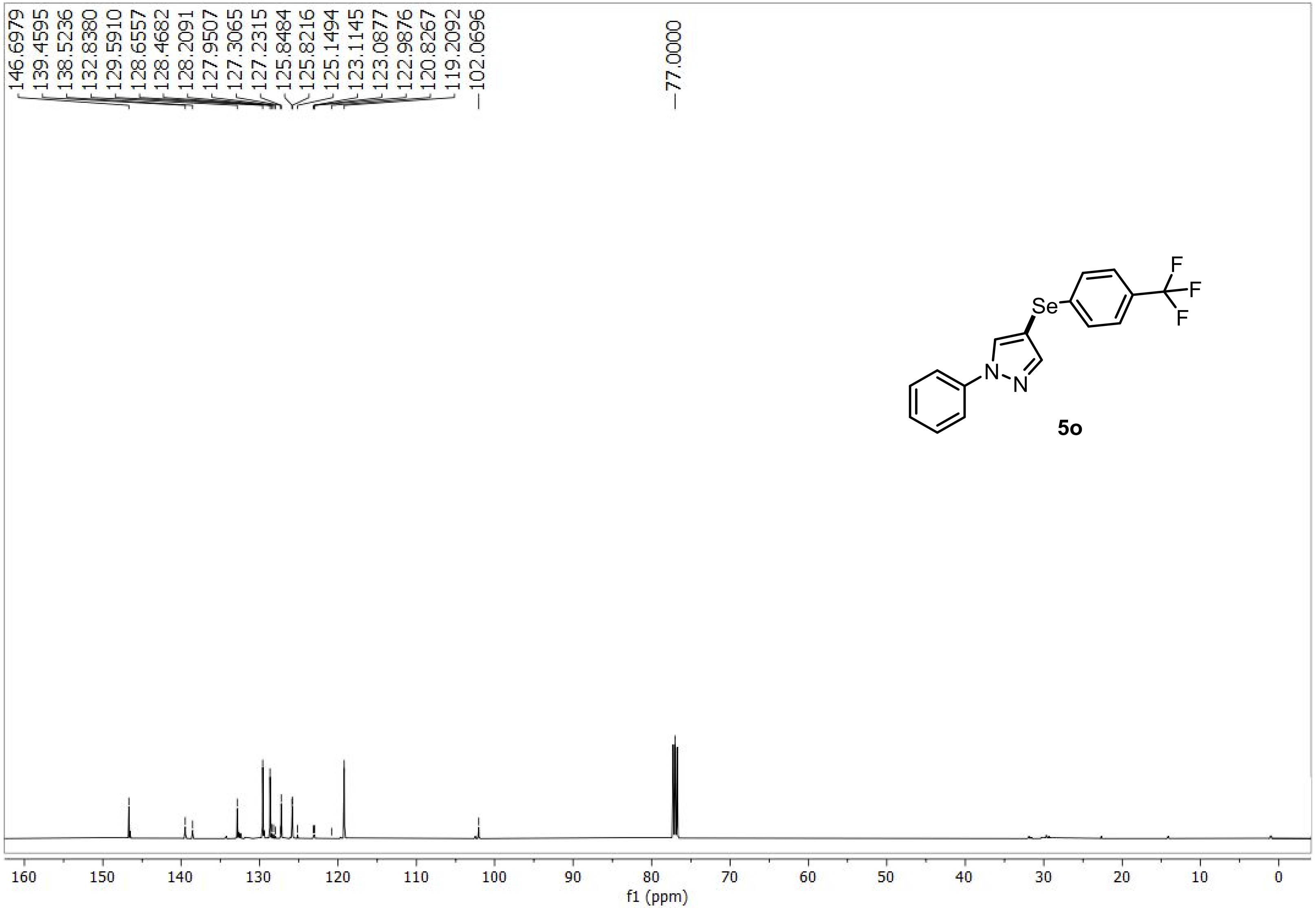

Figure S54: $^{13}C\{^{1}H\}$ NMR (125 MHz, $CDCl_3$) spectrum of compound **5o**.

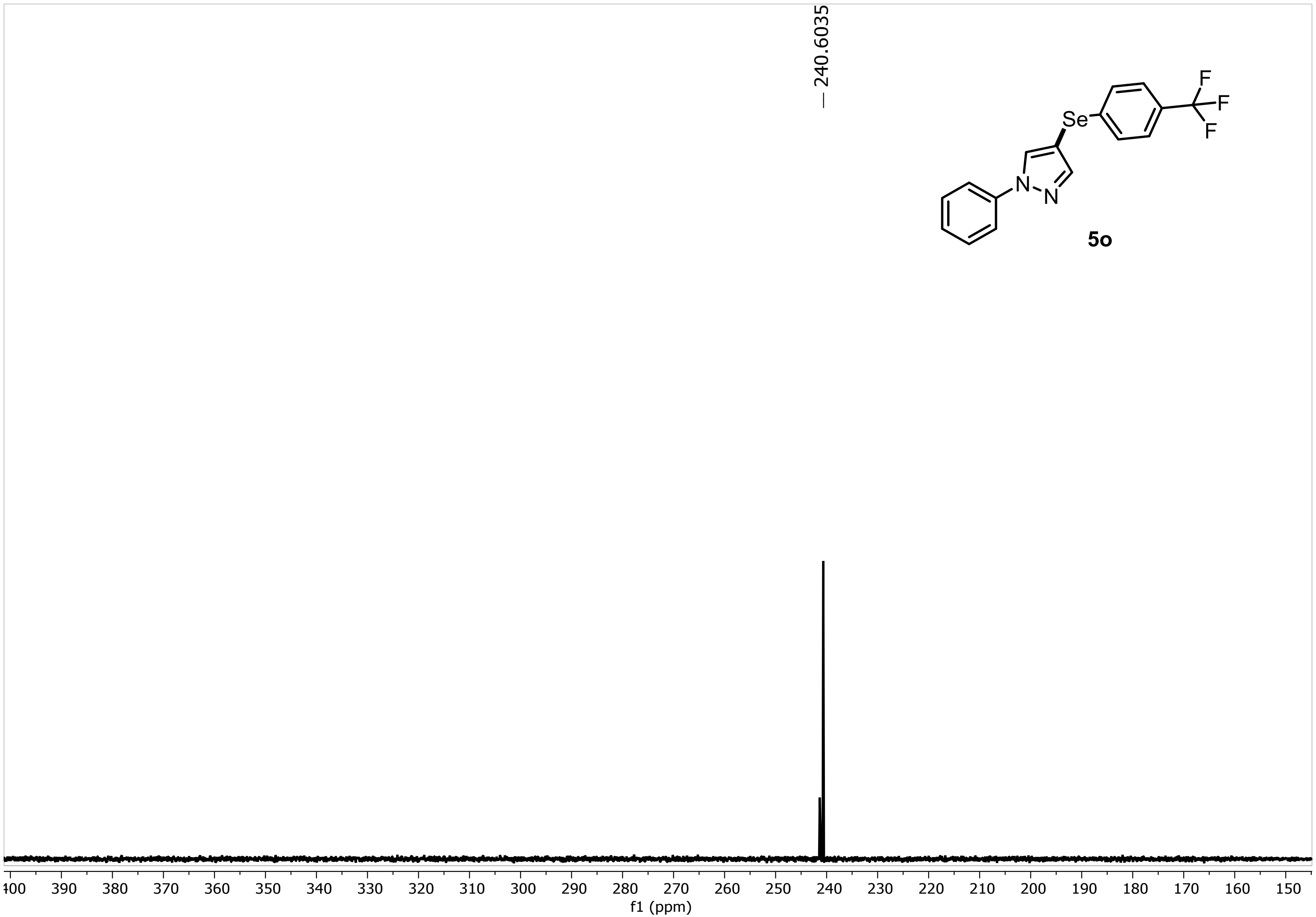

Figure S55: ^{77}Se NMR (95 MHz, $CDCl_3$) spectrum of compound **5o**.

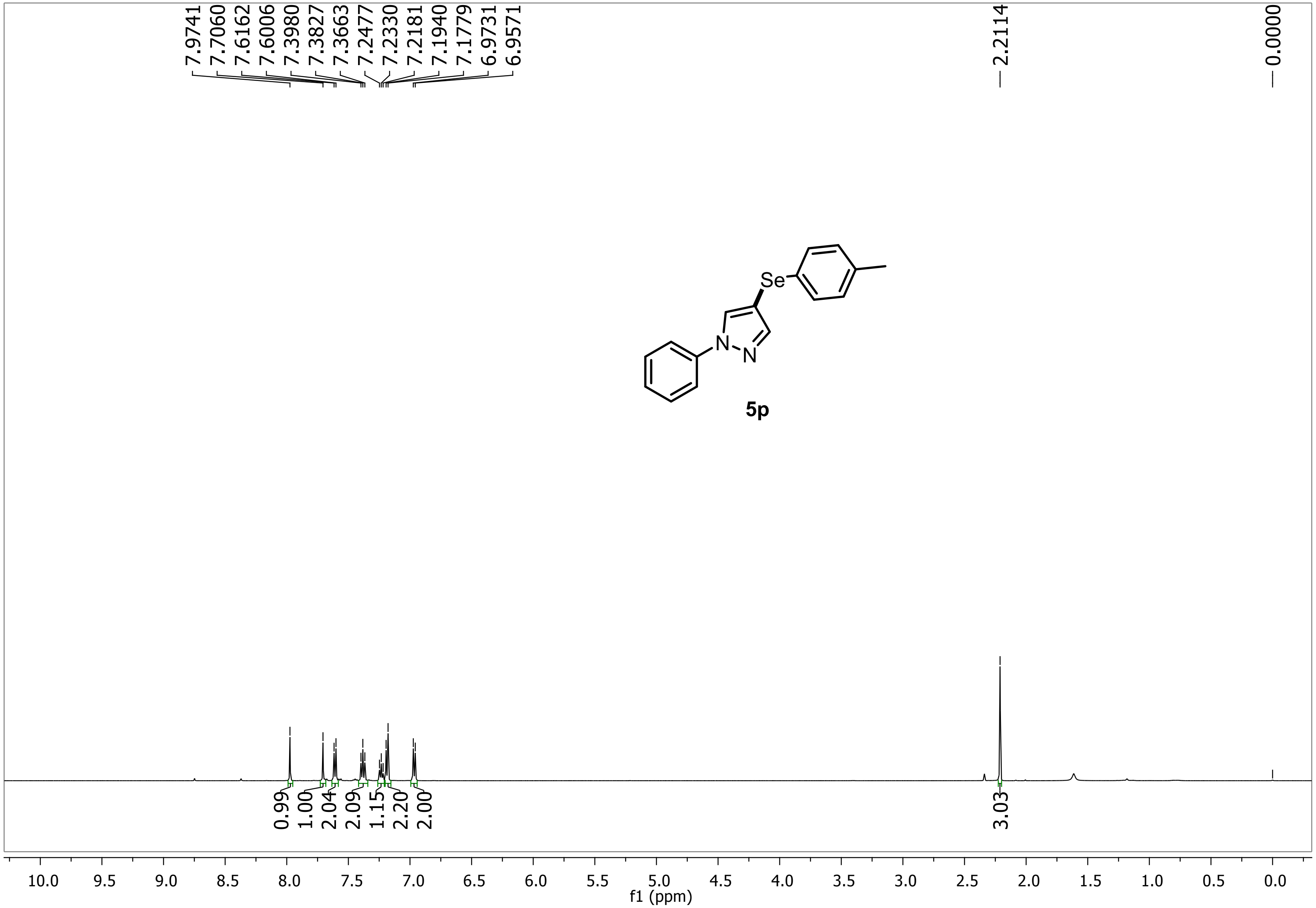

Figure S56: ^{1}H NMR (500 MHz, $CDCl_3$) spectrum of compound **5p**.

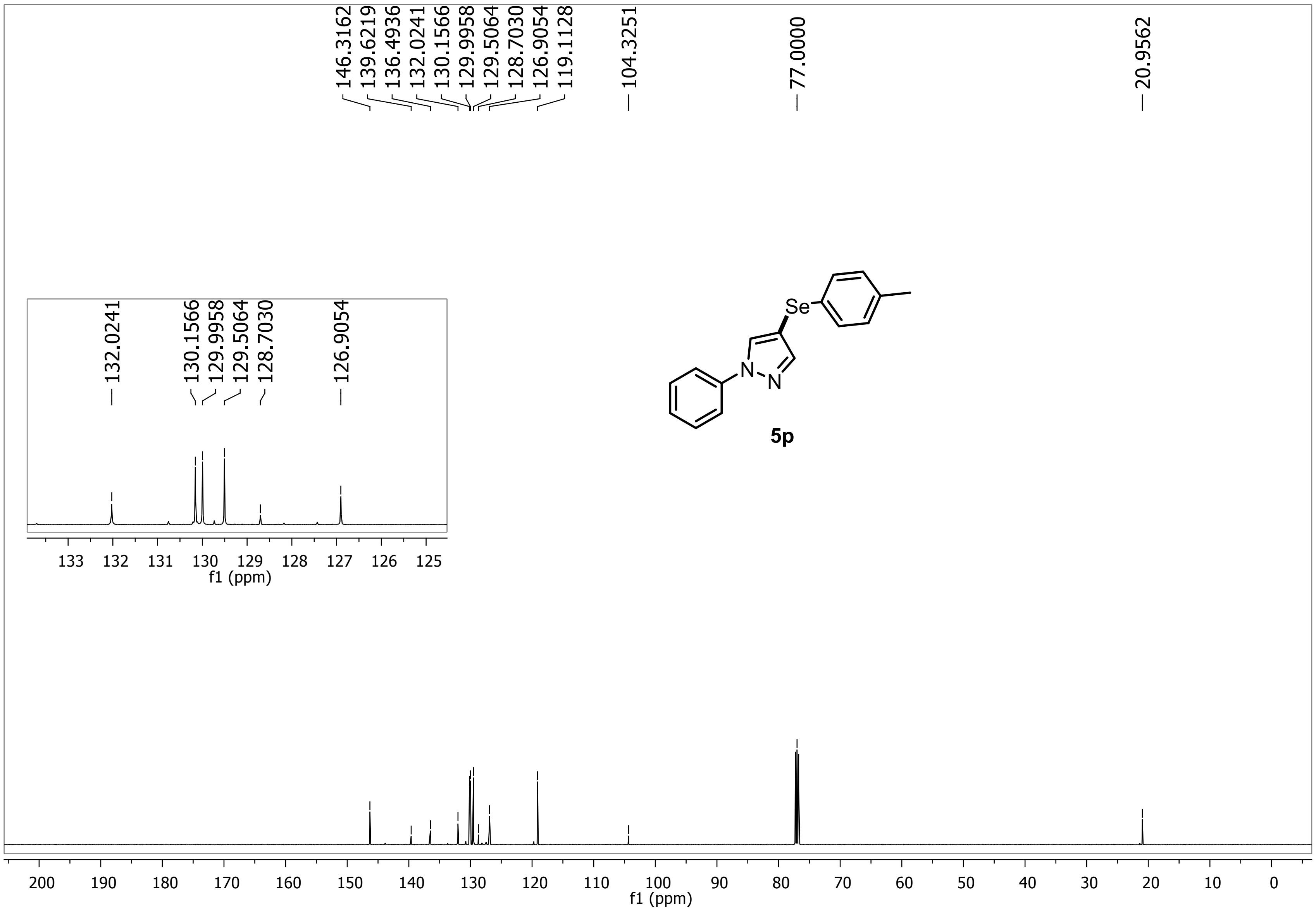

Figure S57: $^{13}C\{^{1}H\}$ NMR (125 MHz, $CDCl_3$) spectrum of compound **5p**.

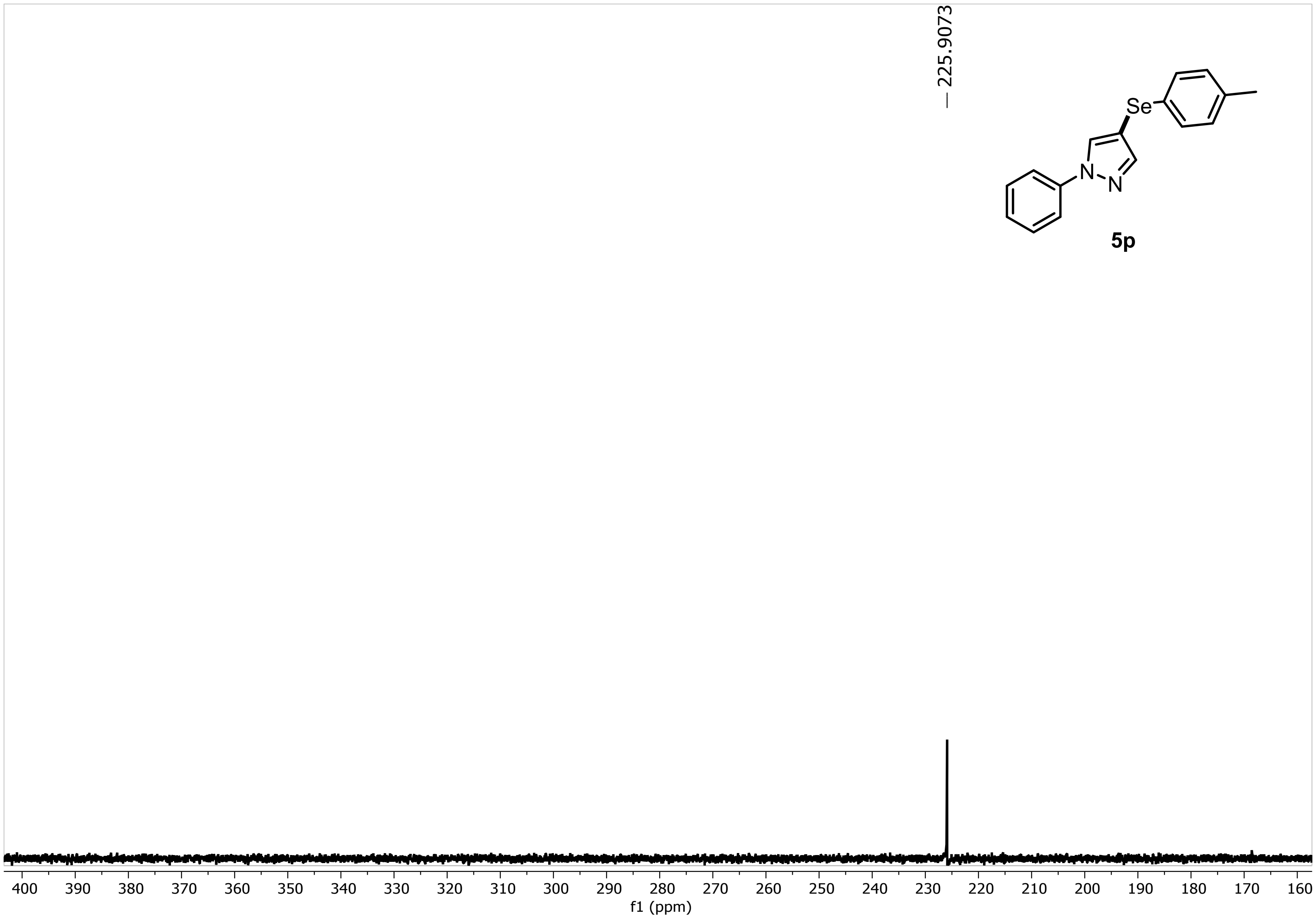

Figure S58: ^{77}Se NMR (95 MHz, $CDCl_3$) spectrum of compound **5p**.

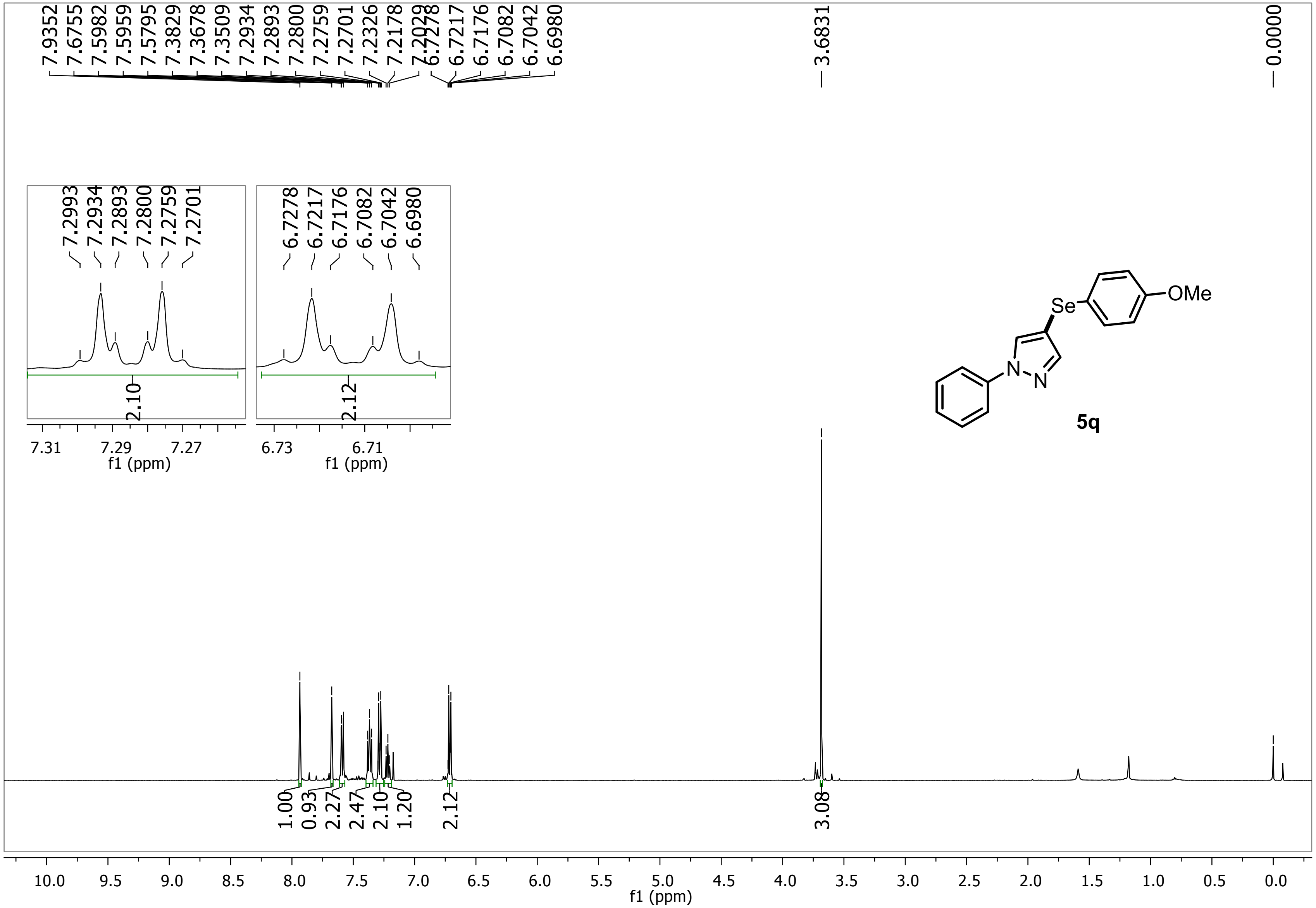

Figure S59: ^{1}H NMR (500 MHz, $CDCl_3$) spectrum of compound **5q**.

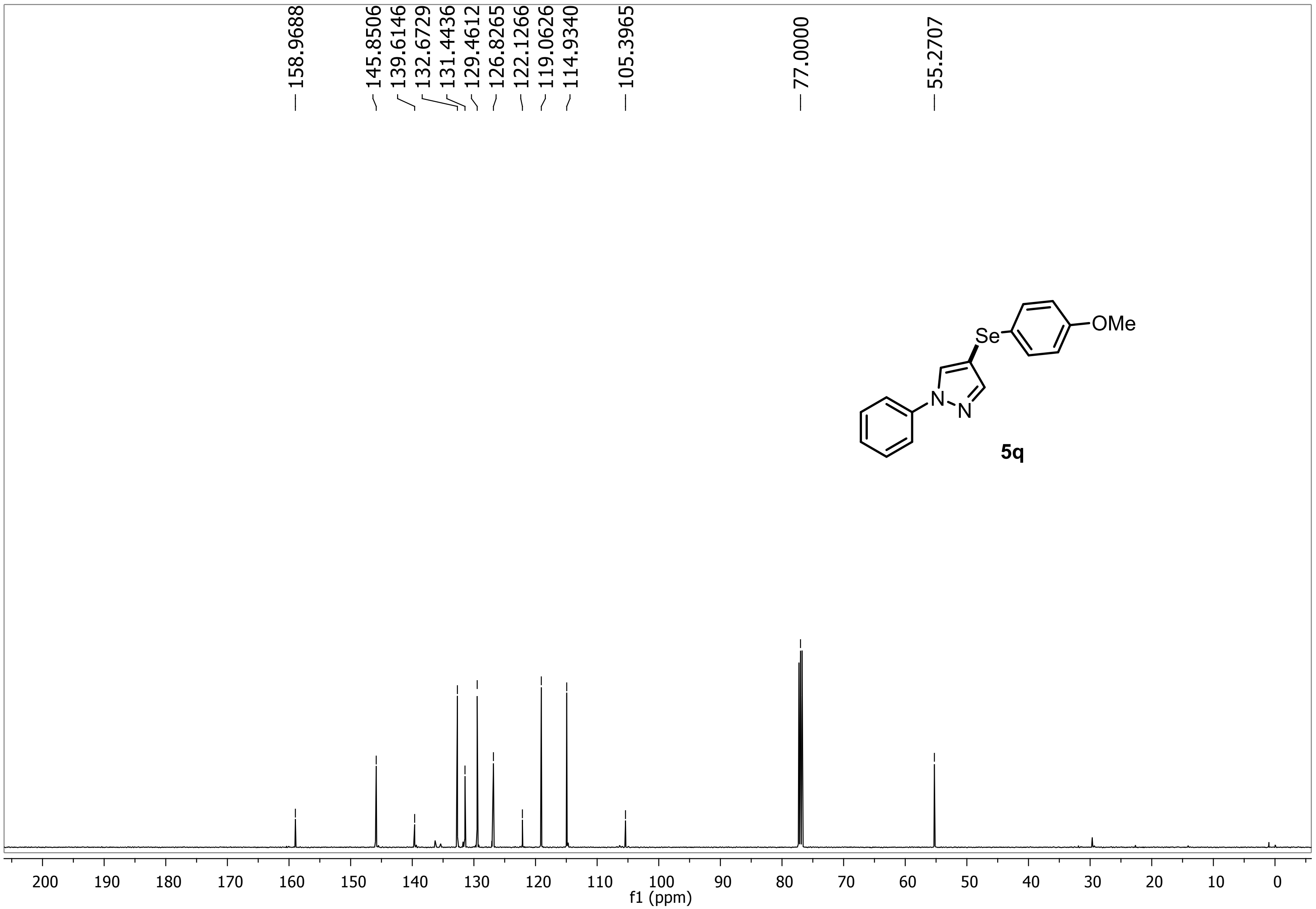

Figure S60: $^{13}C\{^{1}H\}$ NMR (125 MHz, $CDCl_3$) spectrum of compound **5q**.

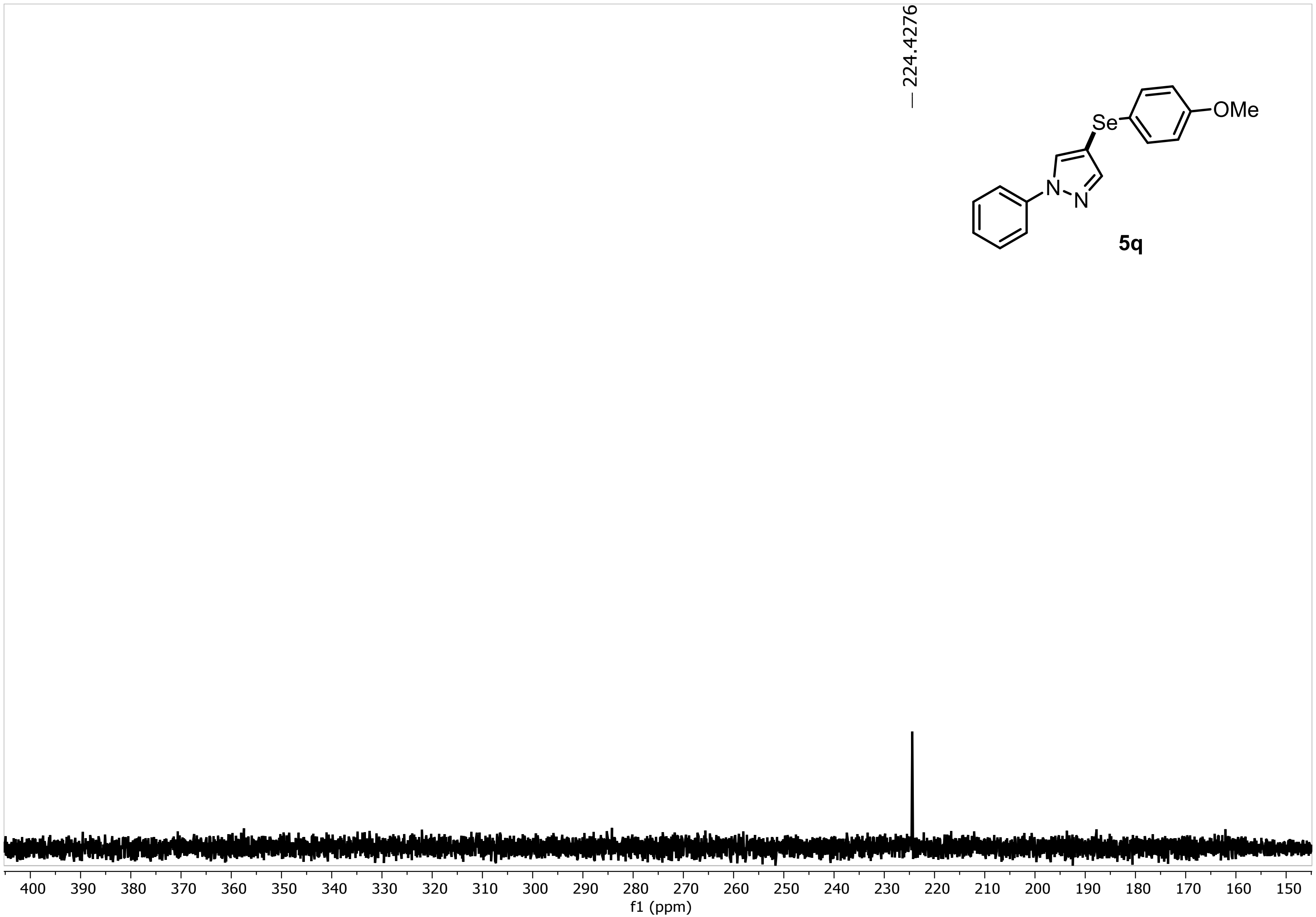

Figure S61: ^{77}Se NMR (95 MHz, $CDCl_3$) spectrum of compound **5q**.

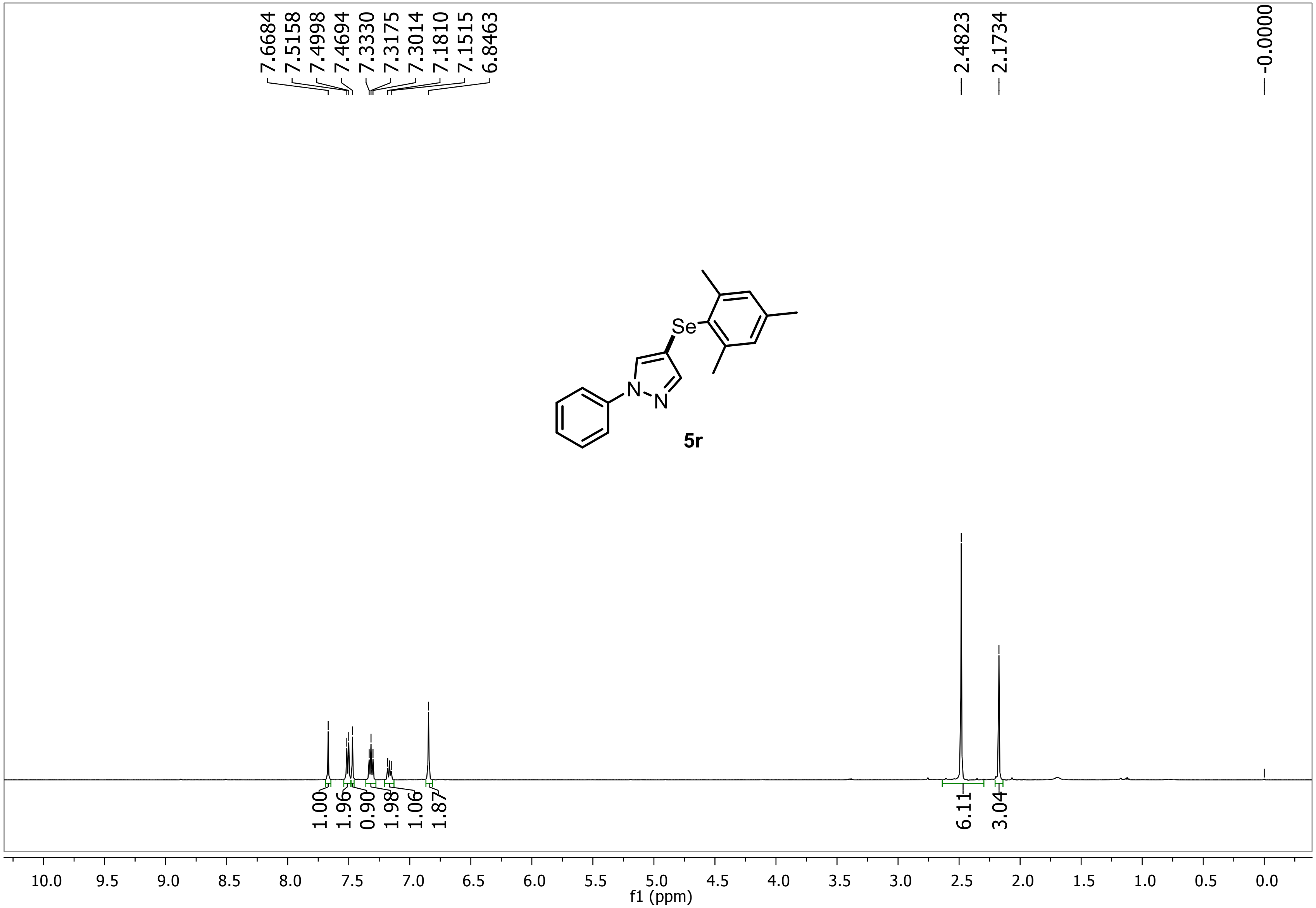

Figure S62: ^{1}H NMR (500 MHz, $CDCl_3$) spectrum of compound **5r**.

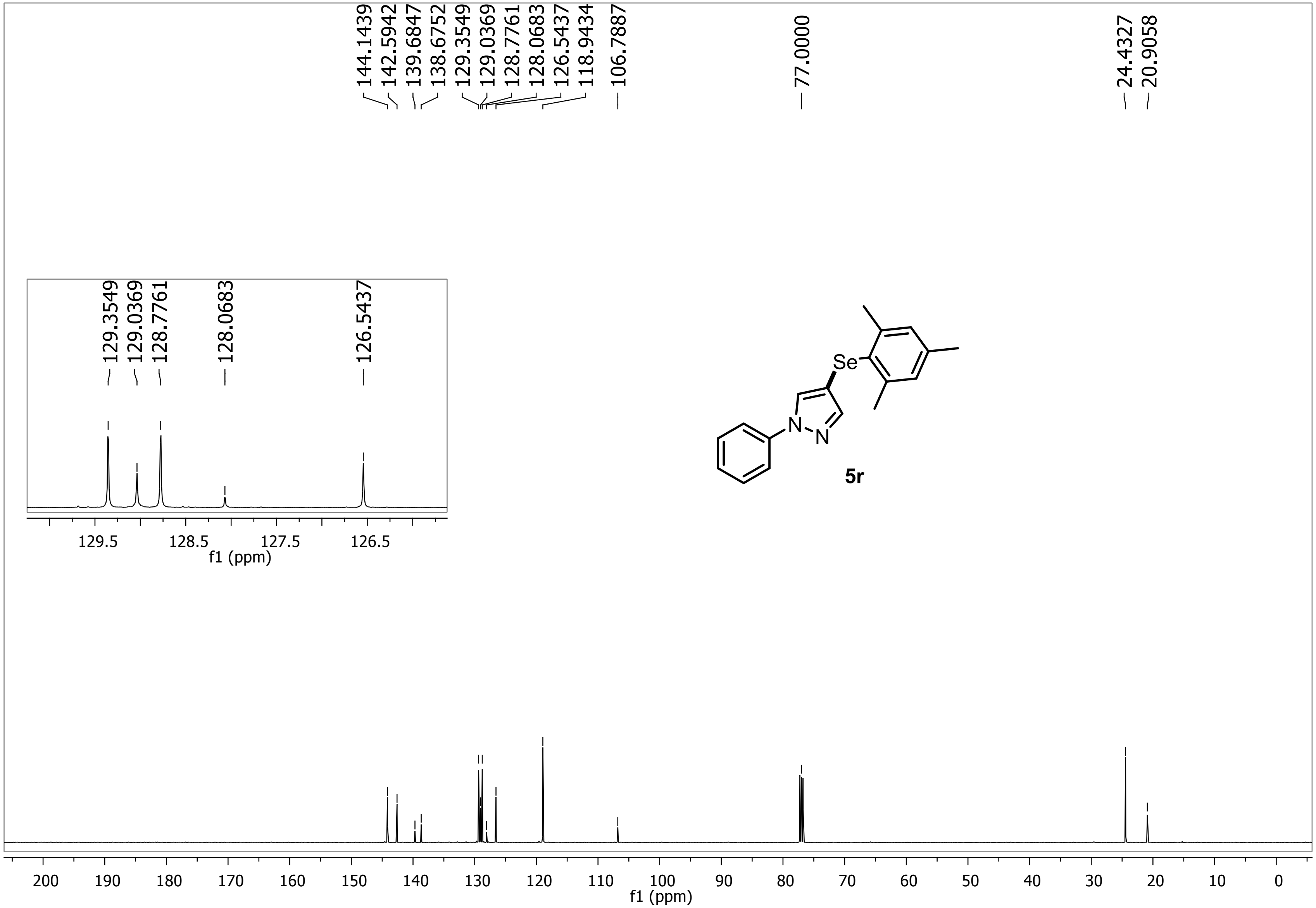

Figure S63: $^{13}C\{^{1}H\}$ NMR (125 MHz, $CDCl_3$) spectrum of compound **5r**.

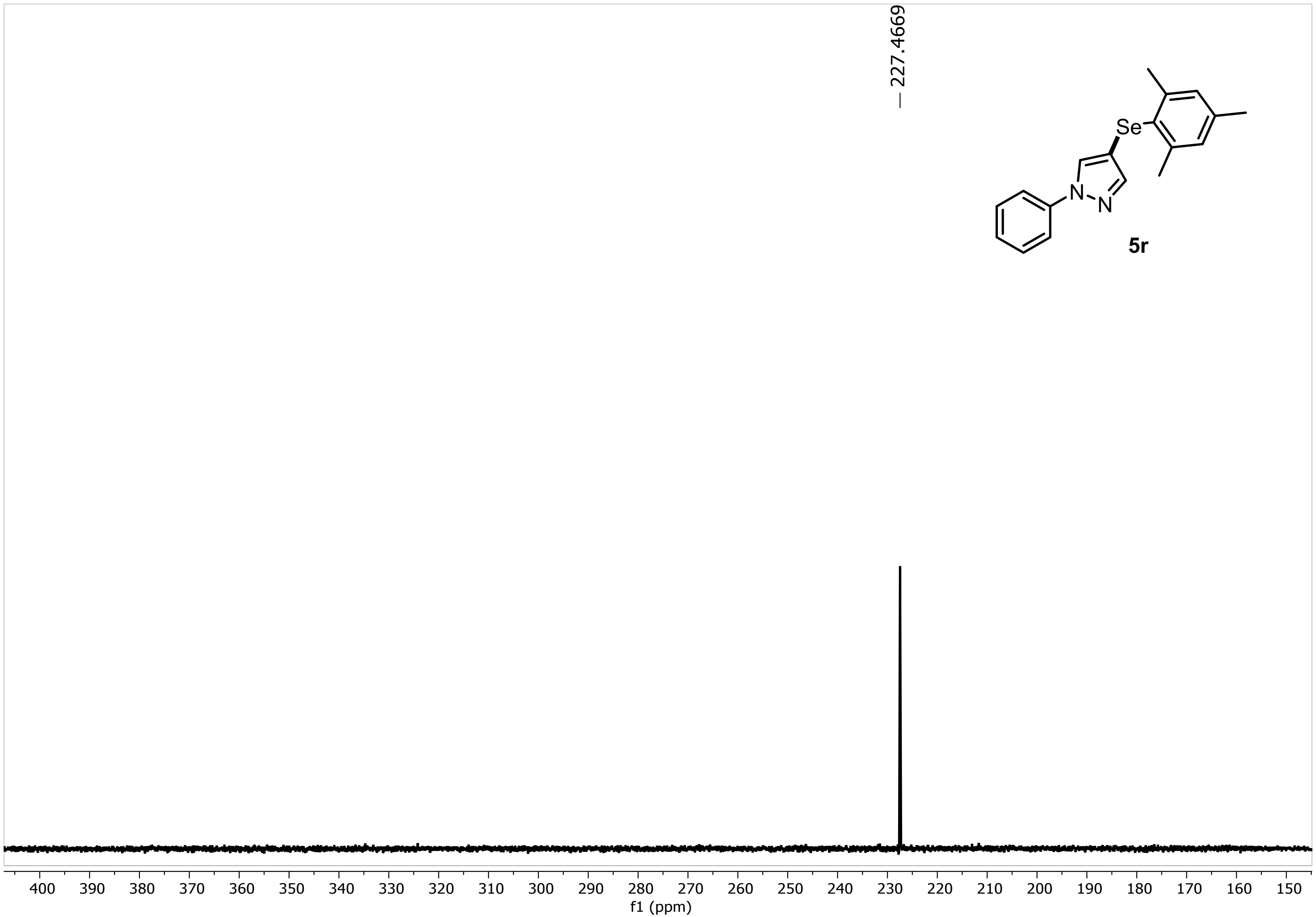

Figure S64: ^{77}Se NMR (95 MHz, $CDCl_3$) spectrum of compound **5r**.

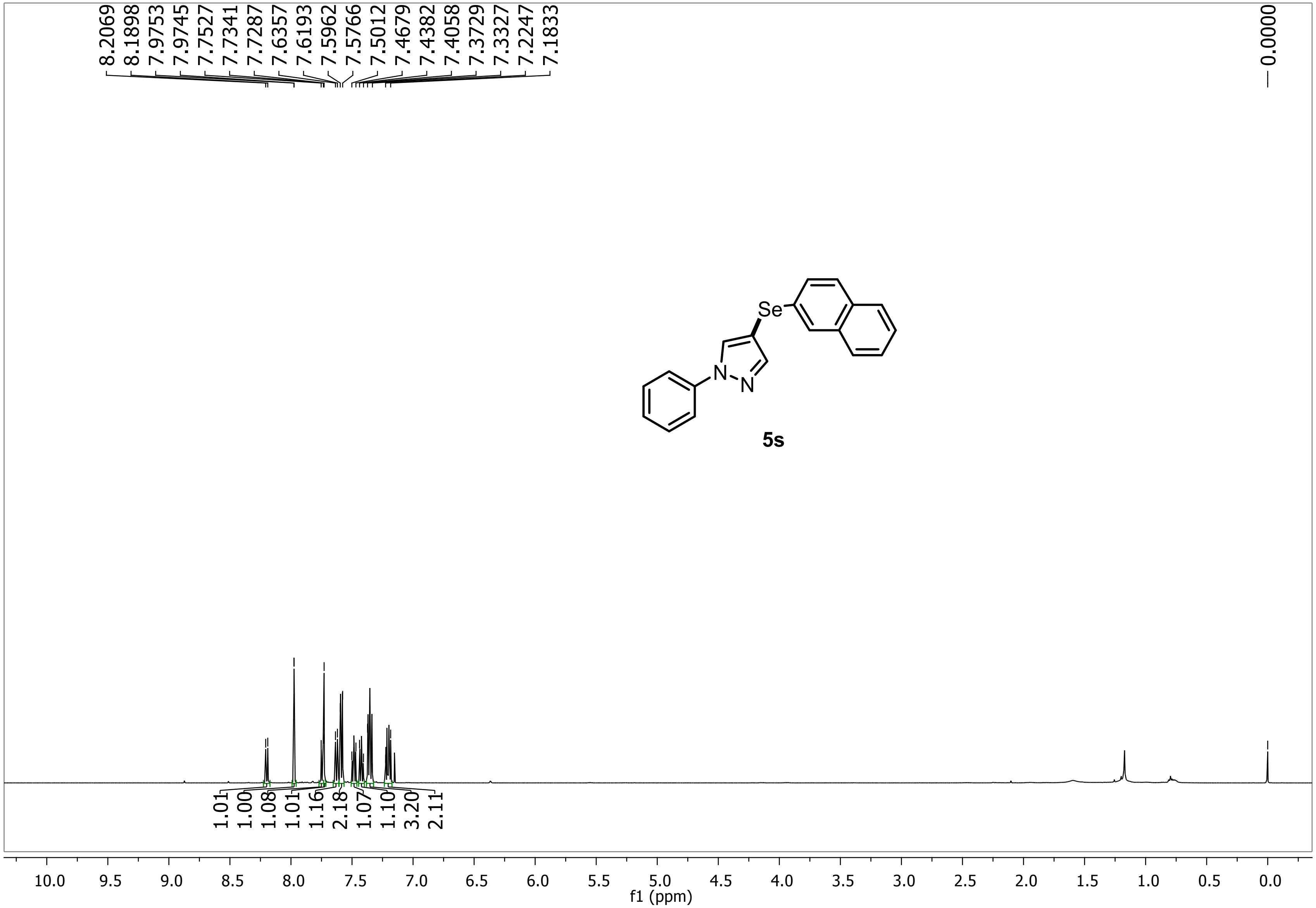

Figure S65: ^{1}H NMR (500 MHz, $CDCl_3$) spectrum of compound **5s**.

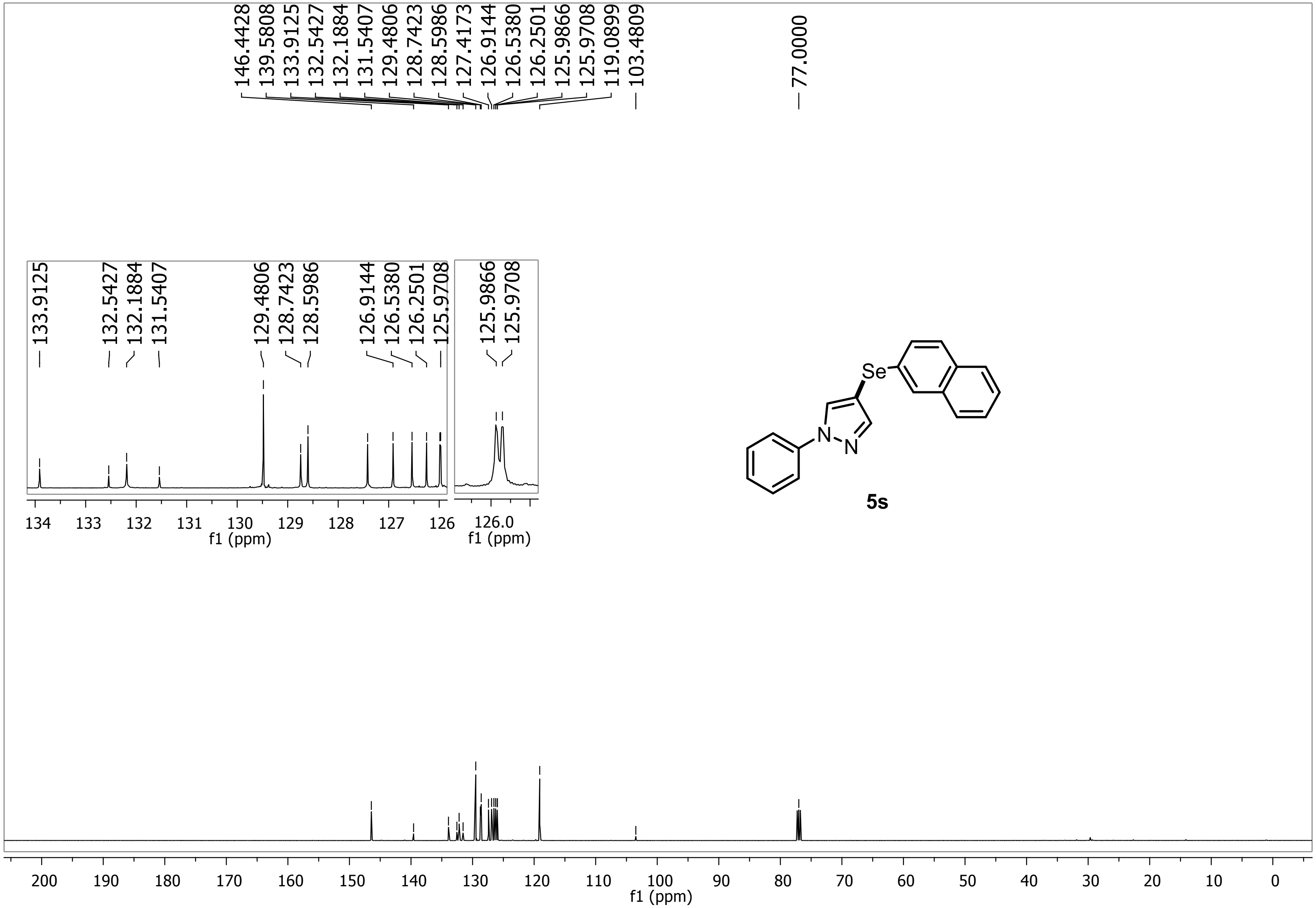

Figure S66: $^{13}C\{^1H\}$ NMR (125 MHz, $CDCl_3$) spectrum of compound **5s**.

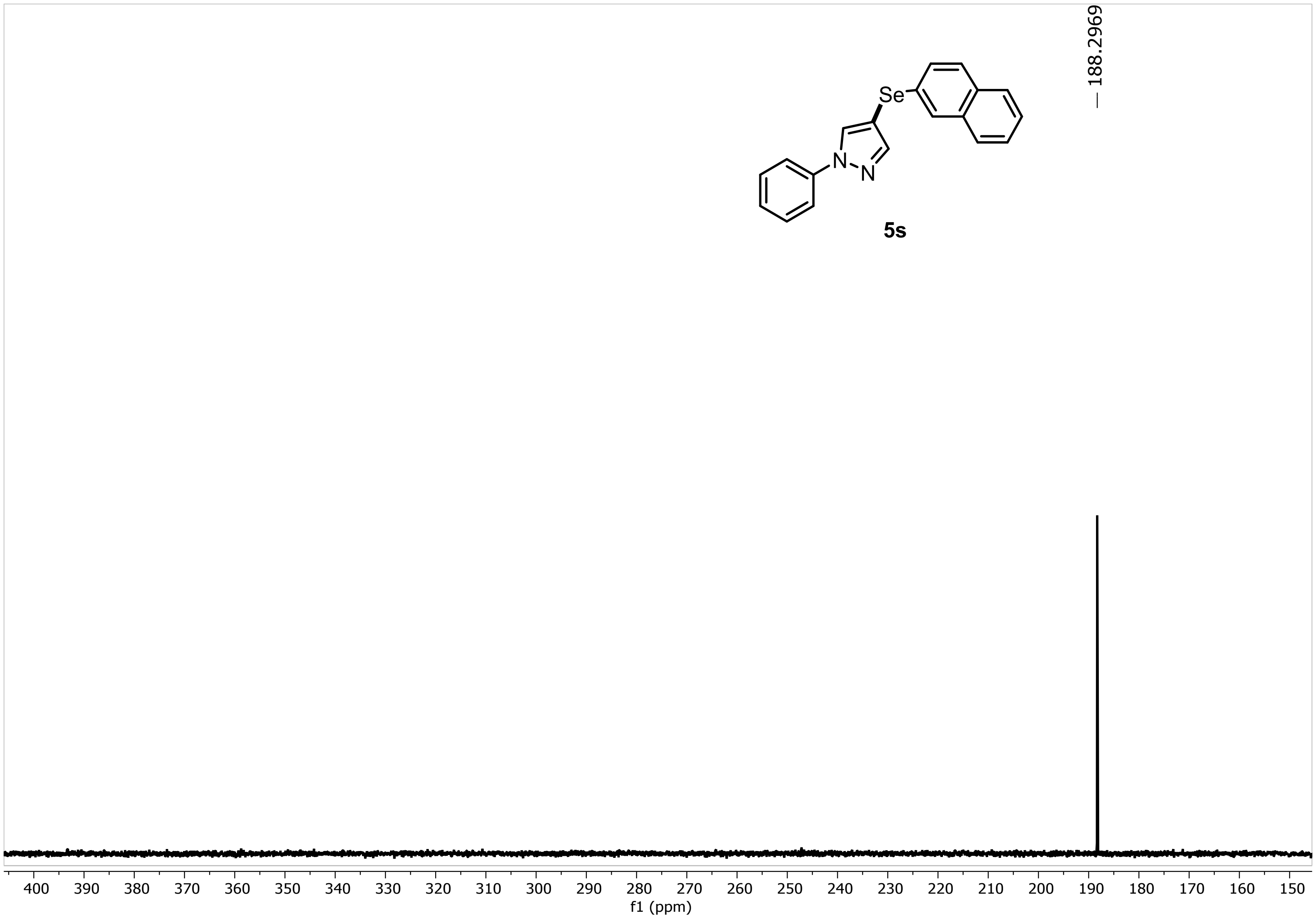

Figure S67: ^{77}Se NMR (95 MHz, $CDCl_3$) spectrum of compound **5s**.

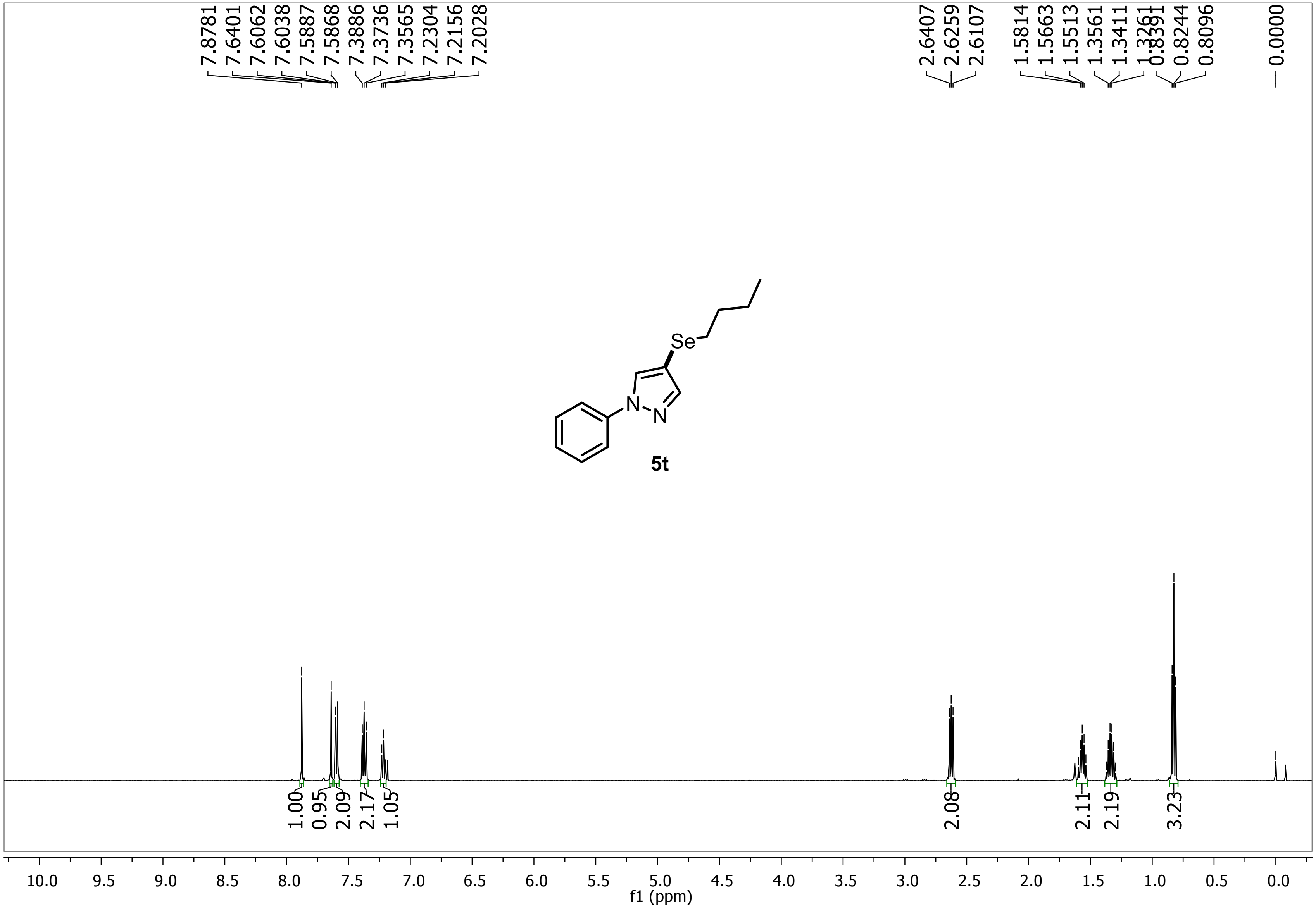

Figure S68: ^{1}H NMR (500 MHz, $CDCl_3$) spectrum of compound **5t**.

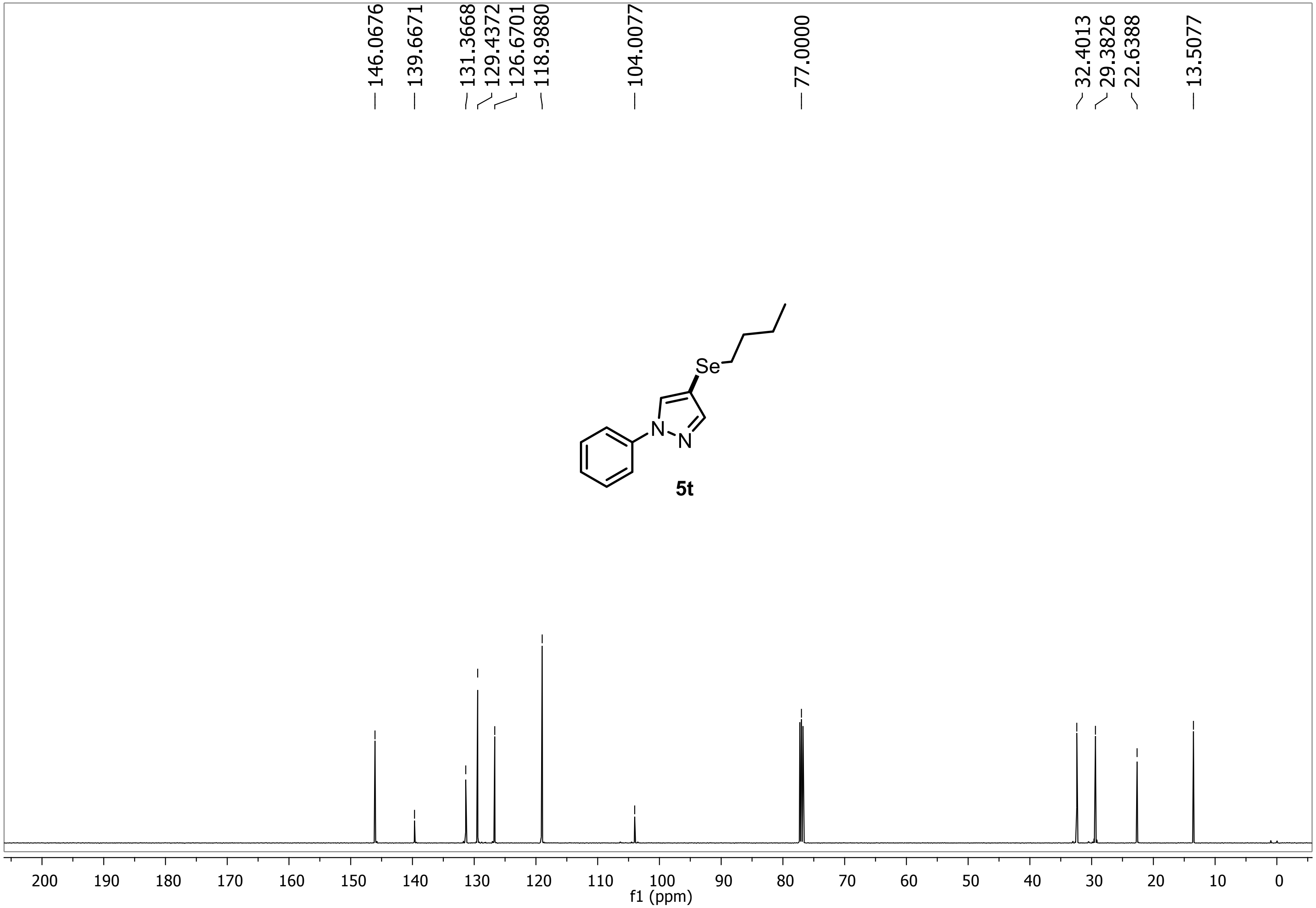

Figure S69: $^{13}C\{^1H\}$ NMR (125 MHz, $CDCl_3$) spectrum of compound **5t**.

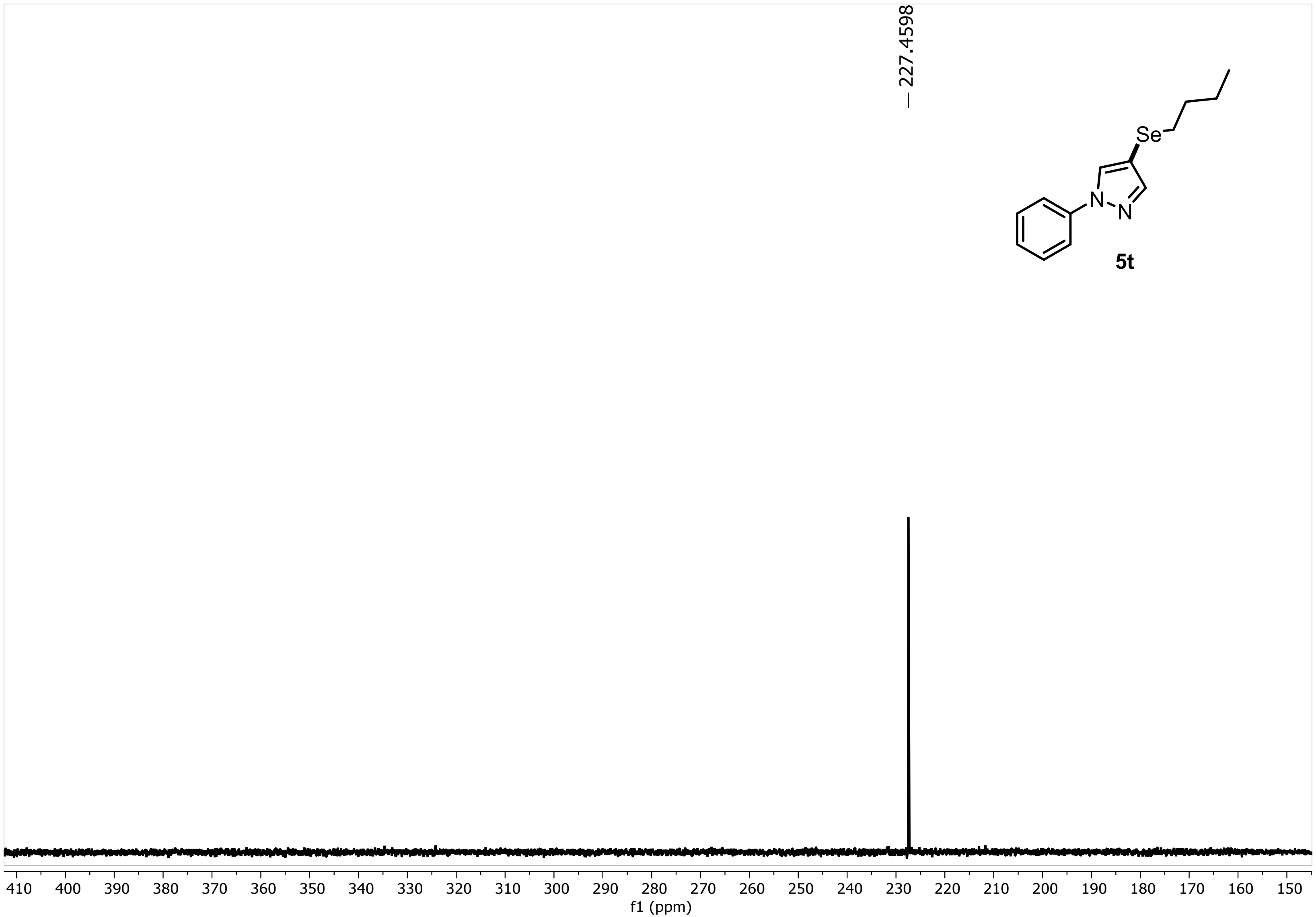

Figure S70: ^{77}Se NMR (95 MHz, $CDCl_3$) spectrum of compound **5t**.
